# Converging Small Ubiquitin-like Modifier (SUMO) and Ubiquitin Signaling: Improved Methodology Identifies Co-modified Target Proteins*[Fn FN2]

**DOI:** 10.1074/mcp.TIR117.000152

**Published:** 2017-09-26

**Authors:** Sabine A. G. Cuijpers, Edwin Willemstein, Alfred C. O. Vertegaal

**Affiliations:** From the ‡Department of Molecular Cell Biology, Leiden University Medical Center, 2300 RC Leiden, the Netherlands

## Abstract

Post-translational protein modifications (PTMs) including small chemical groups and small proteins, belonging to the ubiquitin family, are essential for virtually all cellular processes. In addition to modification by a single PTM, proteins can be modified by a combination of different modifiers, which are able to influence each other. Because little is known about crosstalk among different ubiquitin family members, we developed an improved method enabling identification of co-modified proteins on a system-wide level using mass spectrometry. We focused on the role of crosstalk between SUMO and ubiquitin during proteasomal degradation. Using two complementary approaches, we identified 498 proteins to be significantly co-modified by SUMO and ubiquitin upon MG132 treatment. These targets included many enzymatic components of PTM machinery, involved in SUMOylation and ubiquitylation, but also phosphorylation, methylation and acetylation, revealing a highly complex interconnected network of crosstalk among different PTMs. In addition, various other biological processes were found to be significantly enriched within the group of co-modified proteins, including transcription, DNA repair and the cell cycle. Interestingly, the latter group mostly consisted of proteins involved in mitosis, including a subset of chromosome segregation regulators. We hypothesize that group modification by SUMO-targeted ubiquitin ligases regulates the stability of the identified subset of mitotic proteins, which ensures proper chromosome segregation. The mitotic regulators KIF23 and MIS18BP1 were verified to be co-modified by SUMO and ubiquitin on inhibition of the proteasome and subsequently identified as novel RNF4 targets. Both modifications on MIS18BP1 were observed to increase simultaneously during late mitosis, whereas the total protein level decreased immediately afterward. These results confirm the regulation of MIS18BP1 via SUMO-ubiquitin crosstalk during mitosis. Combined, our work highlights extensive crosstalk between SUMO and ubiquitin, providing a resource for further unraveling of SUMO-ubiquitin crosstalk.

The limited capacity of our genome is compensated for by the processes of alternative splicing and post-translational modification (PTM)^1^. Especially the latter adds an essential additional layer of complexity to our proteome, which is necessary to provide the cell with sufficient functionally different protein states that are needed for efficient regulation of cellular processes and pathways. In addition, PTMs provide the cell with a rapid response mechanism to deal with changing intracellular or environmental conditions. Modification by a PTM can affect the function of a protein in various ways, for example by changing its conformation, localization, binding partners or half-life. Proteins can be modified by chemical groups (including phosphorylation, acetylation and methylation) or by covalent attachment of small proteins (such as ubiquitin, small ubiquitin-like modifier (SUMO), and NEDD8) (1, 2). Ubiquitin and ubiquitin-like proteins have similar modification cascades, consisting of family-member specific activating E1, conjugating E2, and ligating E3 enzymes (3). In addition, each modification can be removed by specific proteases (4).

The interesting phenomenon of crosstalk among post-translational modifications is increasingly receiving more attention (5). Various crosstalk mechanisms are known that provide an additional layer of fine tuning protein functionality. For example, a first modification can influence a second modification on the same target, as is the case for phosphorylation-dependent ubiquitylation (6, 7) and phosphorylation-dependent SUMOylation (8). In addition, modifications can affect the function of the PTM machinery, as exemplified by Neddylation of Cullin components in ubiquitin E3 ligases (9) and acetylation of the SUMO E2 UBC9 (10). Finally, proteins can be modified by specific crosstalk machinery which recognize proteins with a specific PTM and subsequently modify these targets with a second and different PTM, including SUMO-targeted ubiquitin ligases (STUbLs) like RNF4 (11–16). Studying crosstalk among different PTMs can reveal essential information about protein function that would have been missed by focusing on single modifications. Currently, crosstalk between ubiquitin and ubiquitin-like PTMs is mostly studied by using targeted approaches, which for example recently identified an important role for crosstalk between SUMO and ubiquitin in meiotic recombination among chromosomes (17). However, addressing arising questions about crosstalk on an unbiased proteome-wide level is challenging, because proper purification methods are missing due to technical challenges and low stoichiometry of modified proteins (18).

Here, we have developed an improved strategy to purify and identify proteins co-modified by two different small protein PTMs, SUMO, and ubiquitin. This improved method is generic and can be applied to different combinations of these PTMs and will thereby enable us to study the phenomenon of crosstalk on a more comprehensive PTM-wide level.

## EXPERIMENTAL PROCEDURES

### 

#### 

##### Cell Culture and Treatments

U2OS and HEK293T cells were cultured at 5% CO_2_ and 37 °C in DMEM (Thermo Fisher Scientific, Bremen, Germany) including 10% FCS (Thermo Fisher Scientific), 100 U/ml penicillin, and 100 mg/ml streptomycin (Thermo Fisher Scientific). When indicated cells were selected with 2.5 μm puromycin (Calbiochem, Darmstadt, Germany) to obtain stable co-expressing cell lines, treated with 10 μm MG132 (Sigma, Saint Louis, MO) for 6 h to inhibit the proteasome or infected with lentivirus encoding shRNAs at an MOI of 3 to obtain protein knockdown. Cell synchronization was achieved by incubation with 4 mm thymidine (Sigma) or 0.1 μg/ml nocodazole (Sigma) and confirmed by flow cytometry using propidium iodide (Sigma) to visualize cellular DNA content.

##### His_10_-pulldown

Purification of His_10_-SUMO2 conjugates was performed as described before (19). In short, cell lysates were incubated with Ni-NTA beads (Qiagen, Hilden, Germany) overnight at 4 °C, washed and eluted for 30 min at room temperature (RT). When indicated, eluted samples were diluted and treated with the catalytic domain (CD) of USP2 (Boston Biochem, Cambridge, MA) and/or SENP2 (Boston Biochem) for 3 h at RT to deconjugate ubiquitin and/or SUMO respectively from its target proteins.

##### Purification of Co-modified Proteins by His_10_-pulldown and FLAG-immunoprecipitation

Cells were lysed according to our His_10_-pulldown protocol (19) and samples were incubated with Ni-NTA beads, washed and eluted. Upon concentration and stepwise dilution, samples were incubated with anti-FLAG-M2 beads (Sigma). Subsequently, samples were washed and prepared for immunoblotting or mass spectrometry analysis.

##### Electrophoresis and Immunoblotting

Proteins were separated on Novex 4–12% Bis-Tris Plus gradient gels (Life Technologies, Carlsbad, CA) in MOPS buffer for 45 min at 165 Volt and transferred onto Hybond nitrocellulose membranes (GE Healthcare, Chicago, IL) in cold transfer buffer at 25 V for 3 h. Membranes were blocked in PBS containing 0.05% Tween-20 (Merck, Darmstadt, Germany) and 8% milk powder, followed by incubation with primary antibodies. After washing three times in PBS with 0.05% Tween-20 (PBS/T), the membranes were incubated with secondary antibodies and washed another three times in PBS/T. Pierce ECL 2 immunoblotting substrate (Life Technologies) was used to visualize the signal on RX Medical films (Fuji, Tokyo, Japan).

##### Mass Spectrometry Sample Preparation

After digestion with trypsin (Promega, Madison, WI), samples were acidified by trifluoroacetic acid (Sigma). Stage tips containing C18 (Sigma) were activated by passing HPLC-grade methanol (Sigma), washed with 80% acetonitrile (ACN, Sigma) in 0.1% formic acid (FA, Sigma) and equilibrated with 0.1% FA. Upon loading the samples and washing twice with 0.1% FA, the stage tips were dried completely and eluted twice with 80% ACN. The samples were vacuum dried using a SpeedVac RC10.10 (Jouan, Nantes, France), redissolved in 0.1% FA and transferred to autoloader vials before measurement by mass spectrometry.

##### Mass Spectrometry Experimental Design and Statistical Rationale

For each experimental condition at least four biological replicates were performed to allow detection of significant differences, which were all measured in technical triplicate by nanoflow liquid chromatography-tandem mass spectrometry (nanoLC-MS/MS). Samples were measured on an EASY-nLC 1000 system (Proxeon, Odense, Denmark) connected to an Orbitrap Q-Exactive (Thermo Fisher Scientific) through a nano-electrospray ion source. Peptides were separated in a 13 cm analytical column with an inner-diameter of 75 μm, which was packed in-house with 1.8 μm C18 beads (Reprospher, Ammerbuch-Entringen, Germany). A gradient length was used of 60 min from 2% to 95% ACN in 0.1% FA with a flow rate of 200 nl/minute. The data-dependent acquisition mode with a top 10 method was used to operate the mass spectrometer. Full-scan MS spectra were acquired at a target value of 3 × 10^6^ with a resolution of 70,000. The higher-collisional dissociation tandem mass spectra were recorded at a target value of 1 × 10^5^ and a resolution of 17,500 with a normalized collision energy of 25%. The maximum injection times for MS1 and MS2 were respectively 20 ms and 100 ms. For 60 s, the precursor ion masses of scanned ions were dynamically excluded from MS/MS analysis. Ions with a charge of 1 or greater than 6 were excluded from triggering MS2 events.

Subsequently, the raw data analysis was performed using MaxQuant Software version 1.5.3.30 with its integrated search engine Andromeda. A first search was carried out with 20 ppm for precursor ions, followed by a main search using 4.5 ppm. To search against the *in silico* digested proteome containing 92,180 entries of *Homo sapiens* from UniProt (24 March 2016), the mass tolerance of MS/MS spectra were set to 20 ppm. In addition, MS/MS data were searched by Andromeda for potential common mass spectrometry contaminants. Trypsin/P specificity was used to perform database searches, allowing four missed cleavages. In addition, carbamidomethylation of cysteine residues was considered as a fixed modification, whereas oxidation of methionines, N-terminal carbamylation and acetylation, and diGly modification on lysines were considered as variable modifications. Match between runs was performed with a 20 min alignment time window and a 0.7 min match time window, while a minimum peptide length of 7 was used. To consider proteins for quantification, at least two identified peptides were required, including unique and razor peptides. Proteins and peptides were identified using a false discovery rate of 1% (20). Finally, label-free quantification was performed using LFQ settings with fast LFQ disabled to quantify all identified peptides (supplemental Table S1). Because substantial differences among conditions were expected, LFQ normalization by MaxQuant was skipped to prevent undesirable correction among these samples. Proteins identified by the same set of peptides were combined to a single protein group by MaxQuant (supplemental Table S2).

The proteins identified in each sample were further analyzed using Perseus Software version 1.5.2.4. Samples from DMSO and MG132 treated cells were analyzed separately to prevent incorrect imputation. Both data sets were filtered for potentially improper protein identifications by removing proteins that would fit the categories “potential contaminant,” “reverse,” or “only identified by site.” Subsequently, all LFQ intensities were log2 transformed and all experimental replicates for each condition were assigned together in four groups per treatment for the main analysis. Finally, all proteins were removed that were not identified in at least four experimental replicates in at least one of these four groups. For an additional tailored analysis in the supplementary data, the experimental conditions of both approaches were pooled together and assigned to two groups per treatment to increase the statistical power. Proteins that were not identified in at least eight pooled replicates of these two groups were removed. For both analyses missing values were imputed based on the total matrix of each data set, using normally distributed values with a randomized 0.3 width (log2) and a 1.8 down shift (log2).

Two-sample Student T-tests were performed between the SUMO and ubiquitin expressing cell line samples and their corresponding U2OS control samples to obtain *p* values, their FDR corrected q values and differences for each protein. Finally, four volcano plots were created showing these *p* values (as -Log10(p)) on the *y* axis and differences (as Log2FC (fold change)) on the *x* axis for the His_10_-SUMO2/FLAG-ubiquitin purification under DMSO and MG132 conditions, and for the His_10_-ubiquitin/FLAG-SUMO2 purification under DMSO and MG132 conditions.

To identify significantly co-modified proteins, a false discovery rate of 3% was accepted and all proteins with a q value over 0.03 were removed. To increase the reliability of our data set, we overlaid the co-modified proteins identified by both independent purification approaches and thereby obtained two robust lists of proteins co-modified by SUMO and ubiquitin upon DMSO or MG132 treatment. Significance was determined similarly for the additional tailored analysis described above. However, because samples of both approaches were pooled, this analysis directly resulted in one list of proteins co-modified upon DMSO treatment and a second one containing co-modified proteins upon MG132 treatment. Subsequently, the proteins of each list were annotated using the gene ontology annotation of biological processes (GOBP). Enrichment of specific processes was determined by comparison with the Human proteome obtained from Uniprot containing 20577 proteins. Fisher exact tests were performed and the enrichment of a biological process was considered to be significant if its Benjamini-Hochberg FDR value was below 0.03. Additionally, interactions among co-modified proteins were identified using the STRING database version 10.0 with a medium confidence of 0.400. Subsequently this interconnected network and the data from Perseus were imported in Cytoscape version 3.5.0 to visualize the interaction among proteins of specific biological processes and their individual values as a co-modified target.

Although the samples were not specifically enriched for modified peptides, a search was performed by MaxQuant to identify peptides modified by a diGly motif (supplemental Table S3). Subsequently, all peptides modified by a diGly motif that were identified equally or more in the parental control samples, compared with the samples from cell lines expressing both SUMO2 and ubiquitin, were considered as ubiquitylated background binders and therefore removed from the list. In addition, all peptides assigned to the ubiquitin precursor UBA52 instead of to ubiquitin, or with lower quality spectra were removed to obtain a list containing peptides modified by a diGly motif that were specifically identified in the samples containing co-modified proteins. For each peptide the best localization evidence spectrum was retrieved from MaxQuant (supplemental PDF S1). Manual inspection of MS/MS spectra following the Andromeda search was performed to remove potential false positive identifications.

##### GST-RNF4 Binding Experiment

A His_10_-pulldown was performed and the samples were diluted to enable protein renaturing as described above. Samples were incubated with control GST or recombinant GST-RNF4 bound Glutathione Sepharose 4 Fast Flow beads (GE Healthcare) for 2 h at 4 °C while moving. Unbound samples were taken, followed by washing four times with wash buffer containing 50 mm Tris (pH 7.5), 150 mm NaCl, 1% Triton X-100 and protease inhibitors without EDTA. Samples were eluted for 30 min at 1200 rpm in wash buffer supplemented with 20 mm glutathione (Sigma).

## RESULTS

### 

#### 

##### Improved Strategy Enables Purification of Co-modified Proteins

**Fig. 1.**
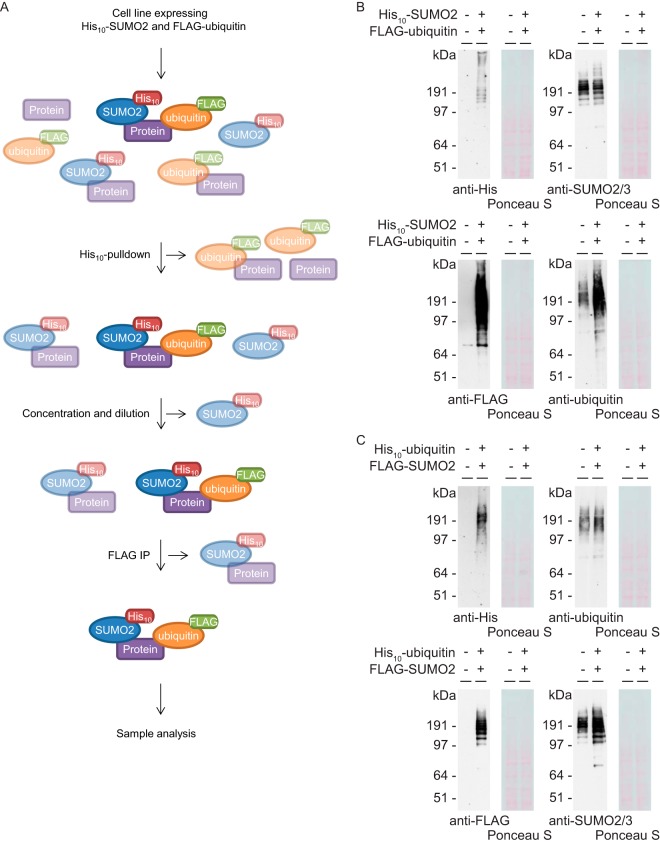
**Improved strategy to purify co-modified target proteins and validation of new cell lines.** *A*, Cartoon depicting the improved strategy to purify proteins simultaneously modified by two different ubiquitin family members, using His_10_-tagged SUMO2 and FLAG-tagged ubiquitin as an example. Cell lines expressing His_10_-SUMO2 and FLAG-ubiquitin are lysed in a denaturing buffer to inactivate proteases and disrupt noncovalent interactions. Upon His_10_-pulldown, the His_10_-SUMO2 and the target proteins covalently attached to this PTM are purified. Samples are concentrated and free His_10_-SUMO2 is removed, followed by a FLAG-IP to enrich for proteins simultaneously modified by SUMO2 and ubiquitin. Co-modified proteins were also purified from cells expressing His_10_-ubiquitin and FLAG-SUMO2, according to a similar strategy. *B*, Parental U2OS cells and U2OS cells expressing His_10_-SUMO2 and FLAG-ubiquitin were lysed and expression levels were analyzed by immunoblotting using antibodies against polyHistidine, SUMO2/3, FLAG and ubiquitin. *C.* Parental U2OS cells and U2OS cells expressing His_10_-ubiquitin and FLAG-SUMO2 were lysed and analyzed by immunoblotting using antibodies against polyHistidine, ubiquitin, FLAG and SUMO2/3.

We have developed an improved method that enables enrichment of proteins simultaneously modified by two different small protein PTMs. Many technical challenges, especially for ubiquitin and ubiquitin-like PTMs, prevented system-wide approaches to uncover novel crosstalk on a comprehensive and proteome-wide level. Our improved method makes use of two consecutive purifications, namely enrichment for a specific His_10_-tagged protein modifier followed by immunoprecipitation (IP) of a different FLAG-tagged protein modifier. As an example, Fig. 1*A* shows the experimental workflow of this method applied on a sample obtained from cells expressing His_10_-SUMO2 and FLAG-ubiquitin. By expressing a differentially tagged version of both protein modifiers of interest at close to endogenous levels, subsequent purifications enabled enrichment of co-modified proteins. In our approach we focused on co-modification of target proteins by two key PTMs, namely ubiquitin and SUMO. However, this method could be used to study crosstalk among many different ubiquitin-like PTMs by simply changing the expressed modifiers.

First, two novel cell lines were created to enable two complementary experimental approaches which would increase the reliability of our data. For the first approach, U2OS cells stably expressing His_10_-SUMO2 (19) were infected with lentivirus encoding a FLAG-ubiquitin construct. Upon selection with puromycin, a stable cell line was created, expressing both His_10_-tagged SUMO2 and FLAG-tagged ubiquitin (Fig. 1*B*). For the complementary approach, an additional cell line was made which expressed both His_10_-tagged ubiquitin and FLAG-tagged SUMO2. To obtain this cell line, U2OS cells stably expressing FLAG-SUMO2 (21) were infected with lentivirus encoding a His_10_-ubiquitin construct and selected with puromycin (Fig. 1*C*). Upon co-purification, the second purification step would enrich co-modified proteins from the pool of SUMOylated target proteins (Approach 1) or from the pool of ubiquitylated target proteins (Approach 2). The overlap between both approaches would be considered as highly reliable co-modified target proteins.

##### Majority of Co-modified Proteins are Directly Modified by SUMO and Ubiquitin

Because our improved method would purify proteins modified directly by SUMO and ubiquitin as well as proteins modified by chains consisting of both SUMO and ubiquitin, an experiment was performed to determine the fraction of proteins modified by such potential mixed chains. A His_10_-pulldown was performed from cells expressing His_10_-SUMO2 and the sample was treated with the catalytic domain (CD) of SENP2 and/or USP2. If both PTMs would be covalently attached directly and independently to their target proteins, the SENP2_CD_ treatment should not affect the ubiquitin signal and the USP2_CD_ treatment should not affect the SUMO2/3 signal (supplemental Fig. S1*A* top). However, if these target proteins would be modified by any form of mixed SUMO-ubiquitin chains, these treatments should co-decrease the SUMO2/3 and/or the ubiquitin signal (supplemental Fig. S1*A* bottom). Immunoblot analysis showed no decrease in the SUMO2/3 signal on USP2_CD_ treatment and no decrease in the ubiquitin signal on SENP2_CD_ treatment, revealing limited purification of target proteins modified by mixed SUMO-ubiquitin and/or mixed ubiquitin-SUMO chains (supplemental Fig. S1*B*). Similar results were obtained from cells treated with MG132, indicating that the role for mixed chains of SUMO and ubiquitin is also limited upon inhibition of the proteasome (supplemental Fig. S1*C*).

##### Identification of Proteins Co-modified by SUMO and Ubiquitin

**Fig. 2.**
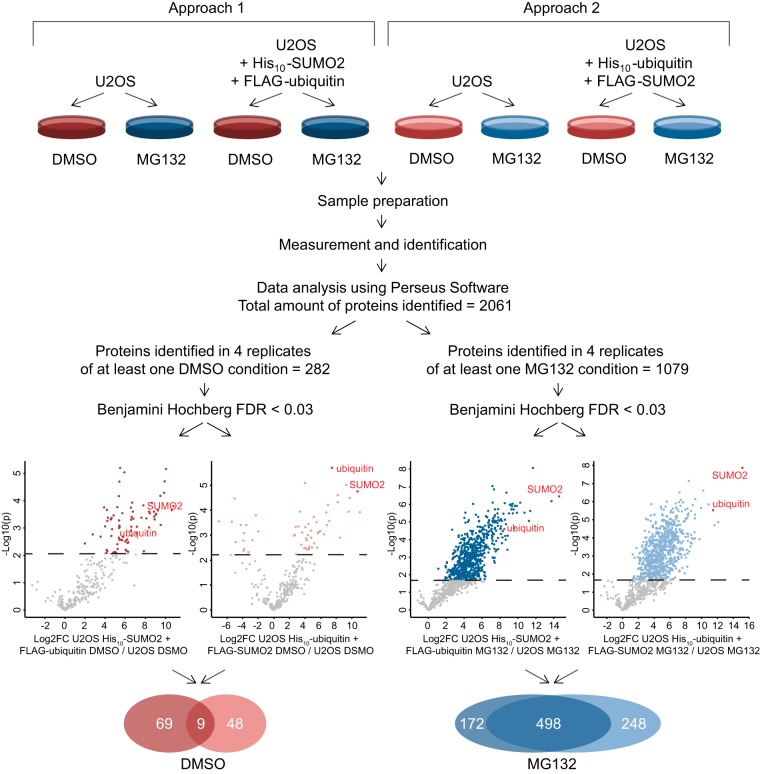
**Schematic overview showing experimental set-up and data analysis.** Two independent approaches were used to identify target proteins co-modified by SUMO and ubiquitin upon DMSO or MG132 treatment. For the first approach, parental U2OS cells and U2OS cells expressing His_10_-SUMO2 and FLAG-ubiquitin were treated with DMSO or MG132 to inhibit the proteasome. Upon performing our improved co-purification strategy, the SUMOylated target proteins were purified first, followed by the enrichment of co-modified proteins by SUMO and ubiquitin. For the complementary second approach, parental U2OS cells and U2OS cells expressing His_10_-ubiquitin and FLAG-SUMO2 were treated with DMSO or MG132. During the co-purification strategy, the ubiquitylated target proteins were purified first, followed by the enrichment of co-modified proteins by SUMO and ubiquitin. Volcano plots for each condition show the *p* value (as -Log10(p)) and difference (as Log2FC (fold change)) for each identified protein, of which the colored targets above the dashed line were significantly enriched with a q value below 0.03. By focusing on the overlap between both approaches, two lists of target proteins were generated consisting of 9 co-modified proteins under control conditions and 498 co-modified proteins upon inhibition of the proteasome.

As shortly mentioned above, two complementary experiments were performed to identify co-modified proteins (Fig. 2 top). For the first approach, we used parental U2OS cells as a negative control and U2OS cells that expressed His_10_-SUMO2 and FLAG-ubiquitin. The second approach made use of parental U2OS cells and U2OS cells expressing His_10_-ubiquitin and FLAG-SUMO2. Because several targeted approaches studying co-modification of single proteins indicated a potential important role for crosstalk between SUMO and ubiquitin in regulating the half-life of proteins by affecting their degradation by the proteasome, we decided to purify co-modified proteins from cells treated with either DMSO as a control or MG132 to inhibit the proteasome. This resulted in four experimental conditions per approach. Upon His_10_-pulldown, SUMOylated targets were purified from the samples in the first approach, followed by enrichment of proteins comodified by both SUMO2 and ubiquitin. Upon His_10_-pulldown from the samples of the second approach, ubiquitylated targets were purified, followed by enrichment of co-modified proteins.

In addition to analysis by mass spectrometry, a fraction of each sample was saved for analysis by immunoblotting to control for purification efficiencies. Equal amounts of starting material were loaded for the samples taken after the first purification (PD) and for the samples taken after double purifications (PD+IP). Immunoblot analysis using an antibody against polyHistidine revealed a decrease in SUMOylated target proteins upon the second purification of the first experimental approach, indicating that only a fraction of the SUMOylated proteins is simultaneously ubiquitylated (supplemental Fig. S2*A*). Analysis of the same samples using an antibody against FLAG revealed limited loss of co-modified targets present among the SUMOylated proteins during the second purification step (supplemental Fig. S2*B*). To assess whether proteins did not crash during dilution and renaturation, equal amounts of starting material were loaded for samples taken after the first purification (PD), of the potential pellet after dilution (pellet) and after both purifications (PD+IP). Immunoblot analysis using an antibody against polyHistidine revealed that most SUMOylated proteins were soluble upon starting the second purification (supplemental Fig. S2*C*). Immunoblot analysis of samples from the second experimental approach indicated that only a limited fraction of the ubiquitylated proteins is also SUMOylated (supplemental Fig. S2*D*), revealed an equally high efficiency of the second purification step (supplemental Fig. S2*E*) and proper renaturation (supplemental Fig. S2*F*).

Samples for mass spectrometry were prepared as described in the experimental procedures section and analyzed using MaxQuant and Perseus Software. In total 2061 proteins were identified among all samples and loaded in Perseus for further analysis. The first selection criterion eliminated any proteins that were not identified in four replicates of at least one of four experimental conditions per treatment, resulting in a decrease to 282 proteins for the DMSO conditions and 1079 proteins for the MG132 conditions. On these lists, four two-sample Student *t*-tests with a Benjamini Hochberg correction were performed between each exogenous SUMO and ubiquitin expressing cell line and their corresponding U2OS control to obtain *p* values, their FDR corrected q values and differences for each protein. Identified proteins were considered co-modified if their q value was below 0.03. Four individual lists of proteins were created containing targets co-modified by SUMO and ubiquitin upon purification from His_10_-SUMO2 and FLAG-ubiquitin or His_10_-ubiquitin and FLAG-SUMO2 expressing cell lines both treated with DMSO or MG132. Volcano plots showing *p* values (as -Log10(p)) on the *y* axis and differences (as Log2FC (fold change)) on the *x* axis confirmed high efficiency of both purifications, because both SUMO2 and ubiquitin were found among the top hits in each of these four lists (Fig. 2 bottom). The 14 “downregulated” hits among the targets enriched from His_10_-ubiquitin and FLAG-SUMO2 expressing cells treated with DMSO are likely to represent background binders, which are more often identified in the empty control samples compared with the less empty positive samples. Both sets of co-modified proteins for each treatment were purified in a complementary order, because the co-modified proteins were enriched from either the purified SUMOylated or ubiquitylated pool of proteins. Therefore, the overlap between both lists provided us with two lists of most highly reliable co-modified target proteins, which identified 9 co-modified proteins under control conditions and 498 proteins modified by SUMO and ubiquitin upon proteasomal inhibition (supplemental Table S4).

Additionally, a tailored analysis was performed by pooling both approaches before checking for significantly co-modified targets and thereby strengthening the statistical power, which revealed 34 co-modified targets under control conditions and identified 699 proteins to be modified by SUMO and ubiquitin after proteasomal inhibition (supplemental Fig. S3*A* and supplemental Table S5). Gene ontology analysis of both lists showed significant enrichment for various biological processes (supplemental Table S6). Interestingly, eight processes were observed to be specifically enriched among the co-modified targets under control conditions and not upon inhibition of proteasomal degradation, indicating a potential role for crosstalk between SUMO and ubiquitin in regulation of DNA modification under control conditions (supplemental Fig. S3*B*/S3*C*).

##### Many Enzymes Involved in the Process of Modification Itself Are Co-modified, Indicating Extensive Crosstalk

**Fig. 3.**
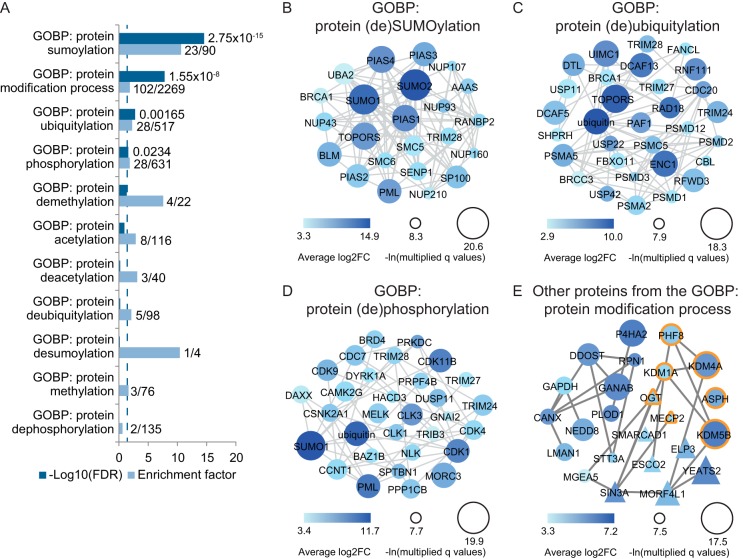
**Detailed analysis of the co-modified targets reveals extensive potential crosstalk among PTMs.** *A*, Significantly enriched co-modified targets upon inhibition of the proteasome were annotated and a gene ontology enrichment analysis was performed. For various biological processes involved in protein modification, their Benjamini Hochberg corrected *p* value (as -Log10(FDR)) and enrichment factor are shown. The dashed line indicates the significance cutoff at an FDR of 0.03. *B*, Co-modified proteins annotated among the biological processes of protein SUMOylation or deSUMOylation were identified and their interactions based on the STRING database as shown. Larger circle size corresponds to a lower q value and darker color indicates a higher difference. *C*, like *B*, except containing co-modified proteins annotated with the biological processes of protein ubiquitylation or deubiquitylation. *D*, like *B*, but showing co-modified proteins annotated to the biological processes of protein phosphorylation and dephosphorylation. *E*, Co-modified proteins annotated to the more general process of protein modification that were not shown in *B–D*, including targets involved in protein (de)methylation (orange border) and (de)acetylation (triangles). Larger shape size corresponds to a lower q value and darker color indicates a higher difference.

Interestingly, within the 498 proteins found by our main and more stringent analysis to be co-modified by SUMO and ubiquitin upon inhibition of the proteasome, many enzymes were identified that are known to be involved in the process of post-translational modification itself. Gene ontology analysis revealed a significant enrichment of proteins involved in the general process of *protein modification*, but also the more specific processes of *protein SUMOylation* and *protein ubiquitylation* (Fig. 3*A* and supplemental Table S6). Analyzing these data in more detail, we identified exactly which proteins involved in (de)SUMOylation and (de)ubiquitylation were co-modified upon inhibition of the proteasome (Fig. 3*B*/3*C* and supplemental Table S7). Although the modifications on these PTM machinery components should be studied in more detail, it could be the result of auto-modification and have limited effect on the function of these proteins. However, to our surprise, we did not only identify co-modification by SUMO and ubiquitin on SUMOylation and ubiquitylation machinery, but also on many enzymes involved in other PTMs. Among the targets identified to be SUMOylated and ubiquitylated upon MG132 treatment, a significant enrichment was observed for proteins involved in the biological process of protein phosphorylation (Fig. 3*D*). Additionally, proteins involved in modification by other PTMs, such as acetylation and methylation, were identified (Fig. 3*E*). These data indicate a potential large network of crosstalk among different PTMs, which might regulate each other's machinery and thereby highlight the complexity and interconnectivity of post-translational protein modifications.

##### Enrichment of Functionally Distinct Protein Groups Including Mitotic Regulators

**Fig. 4.**
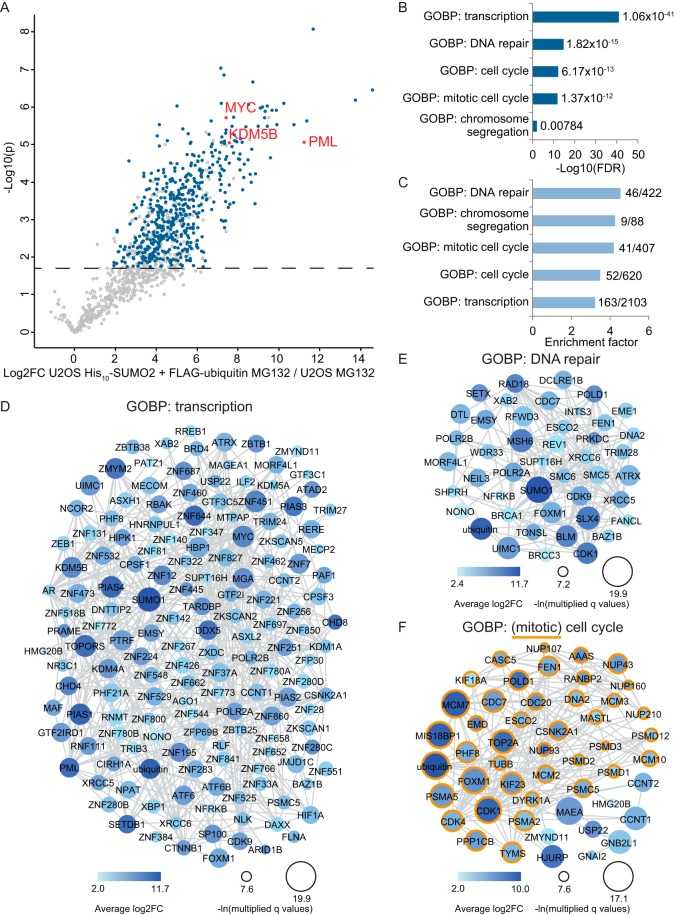
**Enriched protein groups within co-modified targets upon proteasome inhibition.** *A*, Volcano plot showing a group of known STUbL targets in red identified among the significantly co-modified proteins upon MG132 treatment, which are highlighted in blue. *B*, Gene ontology analysis revealed significant enrichment for various biological processes among the identified co-modified targets upon MG132 treatment. For a selection of these processes the FDR value of their enrichment is shown as a -Log10(FDR). *C*, like *B*, but showing the enrichment factor for each of the selected biological processes. *D*, Co-modified proteins annotated among the biological process of transcription were identified and their interactions based on the STRING database as shown. Larger circle size corresponds to a lower q value and darker color indicates a higher difference. *E*, like *D*, but showing co-modified proteins annotated to the biological process of DNA repair. *F*, Co-modified proteins annotated to the biological process of the cell cycle, including targets involved in the more specific process of mitotic cell cycle (orange outline). Larger shape size corresponds to a lower q value and darker color indicates a higher difference.

Although more details about crosstalk between SUMO and ubiquitin remain to be discovered, two human SUMO-targeted ubiquitin ligases (STUbLs) are currently known (11). RNF4 and RNF111 contain SUMO interaction motifs (SIMs), which enable them to bind SUMOylated proteins and subsequently covalently attach ubiquitin to these targets. Until now only a handful of target proteins has been identified for these STUbLs, mostly through targeted approaches. Using our improved system-wide method many more proteins were found to be co-modified by SUMO and ubiquitin specifically upon inhibition of the proteasome, which could also include STUbL targets. This was verified by the identification of the known RNF4 targets PML, MYC, and KDM5B (22–24) among our 498 hits (Fig. 4*A*). In addition to these known STUbL targets, many more proteins were identified whose degradation is potentially regulated through crosstalk between SUMO and ubiquitin. To analyze these potential STUBL targets, a gene ontology enrichment analysis was performed (supplemental Table S6). A selection of various significantly enriched biological processes is shown in Fig. 4*B*/4*C*. More detailed analysis revealed which proteins involved in transcription and DNA repair were identified to be SUMOylated and ubiquitylated upon inhibition of the proteasome (Fig. 4*D*/4*E*). Interestingly, the significantly enriched group of proteins involved in the biological process of the cell cycle mostly consisted of proteins with important roles in mitosis (Fig. 4*F*).

**Fig. 5.**
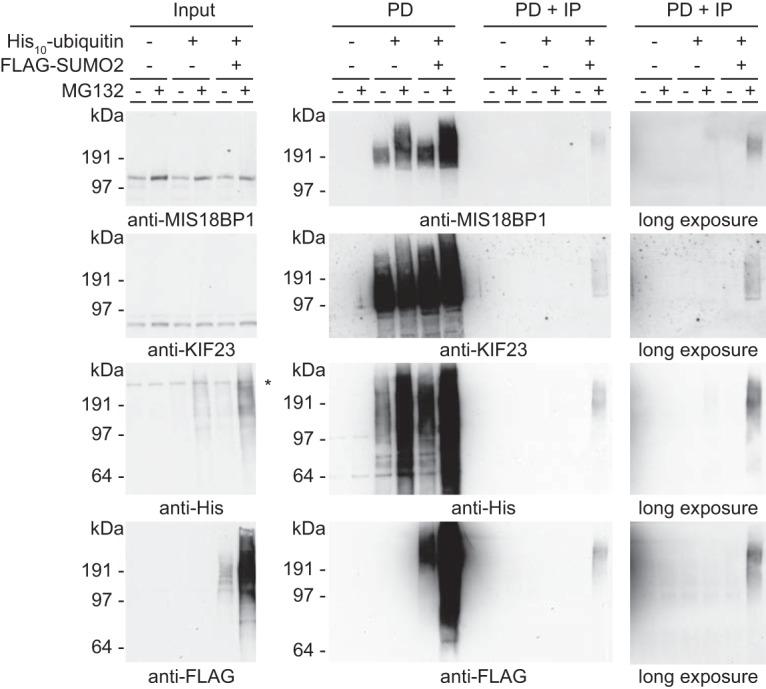
**Verification of MIS18BP1 and KIF23 as co-modified targets upon inhibition of the proteasome.** Parental U2OS cells, U2OS cells expressing His_10_-ubiquitin and U2OS cells expressing His_10_-ubiquitin and FLAG-SUMO2 were treated with DMSO or MG132 to inhibit the proteasome. Samples were taken before the His_10_-pulldown (input), after the His_10_-pulldown (PD) and after the FLAG-IP (PD+IP), and analyzed by immunoblotting with antibodies against MIS18BP1, KIF23, polyHistidine and FLAG. An equal percentage of the sample was loaded for the PD and PD+IP samples to enable comparison. The asterisk represents an a-specific band.

The striking identification of this interesting group of mitotic regulators was also confirmed by the identification of a significant enrichment for the more specific group of proteins involved in chromosome segregation. We therefore decided to verify the mass spectrometry data by analyzing two newly identified proteins with important roles during mitosis via immunoblotting. Co-modification upon inhibition of the proteasome of both MIS18BP1 and KIF23 was confirmed by immunoblot analysis using both complementary purification approaches (supplemental Fig. S4 and S5; Fig 5).

##### Follow-up Reveals KIF23 and MIS18BP1 as Novel RNF4 Targets

**Fig. 6.**
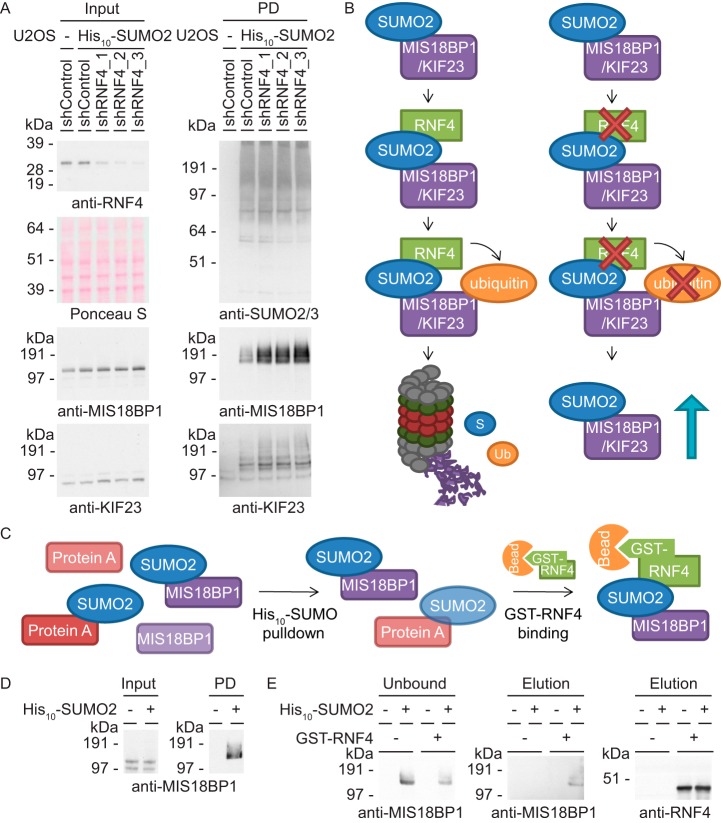
**Identification of MIS18BP1 and KIF23 as novel RNF4 targets.** *A*, U2OS cells expressing His_10_-SUMO2 were infected with lentivirus encoding control shRNAs or three independent shRNAs against RNF4. Input and PD samples were analyzed by immunoblotting using antibodies against RNF4, MIS18BP1, KIF23, and SUMO2/3. *B*, Cartoon depicting the role of RNF4 in the regulation of MIS18BP1 and KIF23. Both important mitotic regulators are SUMOylated and thereby recognized by RNF4, which subsequently ubiquitylates these proteins and targets them for degradation by the proteasome. Upon knockdown of endogenous RNF4, the SUMOylated fraction of MIS18BP1 and KIF23 can no longer be ubiquitylated, which results in stabilization of their SUMOylated fraction. *C*, Cartoon showing the experimental set-up used to study binding of MIS18BP1 to RNF4. After enrichment for SUMOylated proteins by His_10_-pulldown, the samples were incubated with GST or GST-RNF4 bound to beads to purify RNF4 binders. *D*, U2OS cells without or with stable expression of His_10_-SUMO2 were synchronized into mitosis and lysed for pulldown. Input and His_10_-SUMO2 pulldown samples were analyzed by immunoblotting with an antibody against MIS18BP1. *E*, After incubation of the pulldown samples with GST or GST-RNF4 bound to beads, unbound samples were taken and analyzed by immunoblotting against MIS18BP1. Upon washing, the samples were eluted and analyzed by immunoblotting with antibodies against MIS18BP1 and RNF4.

Knockdown of the STUbL RNF4 is known to result in chromosome segregation errors (25), indicating a regulatory role for this STUbL during mitotic progression. However, we are limited in our understanding of the relevant target proteins. Interestingly, gene ontology analysis revealed a significant enrichment for proteins involved in the biological process of chromosome segregation among the targets identified in our screen as co-modified proteins upon inhibition of the proteasome and consequently as potential novel RNF4 targets. To test this hypothesis, U2OS cells expressing His_10_-SUMO2 were infected with lentiviruses encoding three independent shRNAs directed against RNF4. As negative controls, both parental U2OS cells and His_10_-SUMO2 expressing U2OS cells were infected with a lentivirus encoding a nontargeting control shRNA. After His_10_-SUMO pulldown, immunoblot analysis showed increased SUMOylation levels of MIS18BP1 and KIF23 upon knockdown of RNF4 and thereby revealed both mitotic regulators as novel targets of the STUbL RNF4 (Fig. 6*A*). Under control conditions, SUMOylated MIS18BP1 and KIF23 are recognized by RNF4 and subsequently ubiquitylated, resulting in proteasomal degradation of the co-modified protein fraction (Fig. 6*B*). Upon knockdown of RNF4, the SUMOylated fraction of MIS18BP1 and KIF23 is no longer co-modified and degraded, resulting in a stabilization of this specific fraction.

To confirm direct regulation by RNF4, an *in vitro* binding assay was performed (Fig. 6*C*). U2OS cell lines without and with stable expression of His_10_-SUMO2 were synchronized into mitosis by releasing them for 16 h from a thymidine block. Input samples were taken, a His_10_-SUMO pulldown was performed to enrich for SUMOylated proteins and samples were diluted to allow protein renaturation. Immunoblot analysis showed the presence of SUMOylated MIS18BP1 in the pulldown sample from His_10_-SUMO2 expressing cells (Fig. 6*D*). Incubation of both pulldown samples with either control or GST-RNF4 bound beads enabled purification of RNF4 interacting proteins. Although we have been unable to detect direct binding of KIF23 to RNF4, immunoblot analysis did reveal binding of SUMOylated MIS18BP1 to this STUbL (Fig. 6*E*) and thereby provided additional evidence for regulation of SUMOylated MIS18BP1 by RNF4.

##### Dynamic Modification of MIS18BP1 Indicates Regulation of Mitosis by RNF4

**Fig. 7.**
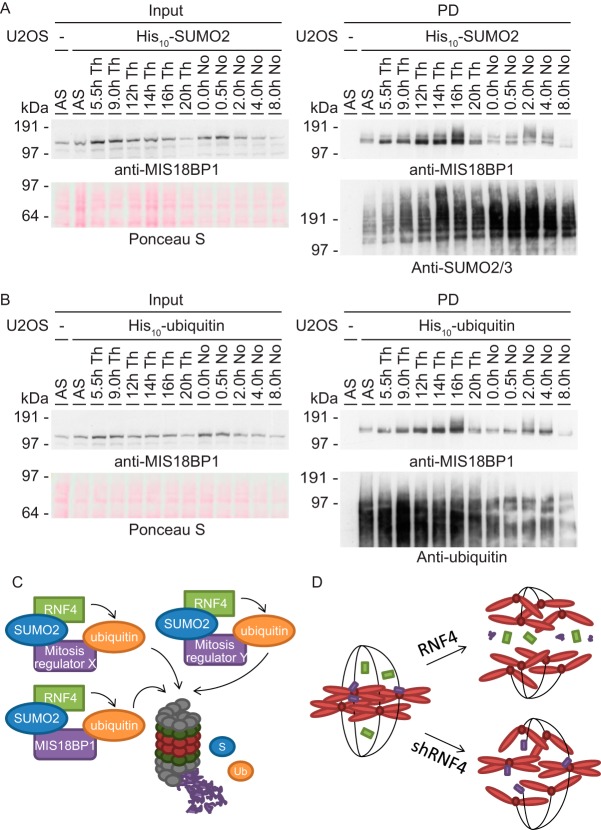
**Regulation of MIS18BP1 during mitosis and the role of RNF4.** *A*, U2OS cells expressing His_10_-SUMO2 were synchronized using two independent cell cycle blocking agents and released for the indicated time periods to reach various cell cycle phases. A His_10_-pulldown was performed and the samples were analyzed by immunoblotting using antibodies against MIS18BP1 and SUMO2/3. *B*, U2OS cells expressing His_10_-ubiquitin were synchronized as described above. Input and pulldown samples were analyzed by immunoblotting with antibodies against MIS18BP1 and ubiquitin. *C*, Cartoon depicting the regulation of various proteins with important roles during mitosis, including MIS18BP1, by the SUMO-targeted ubiquitin ligase RNF4. A subset of mitotic regulators is recognized by RNF4 through its SUMOylation and subsequently ubiquitylated and degraded by the proteasome. *D.* Cartoon showing regulation of chromosome segregation by RNF4. Knockdown of RNF4 results in mitotic problems including chromosome segregation errors. We hypothesize that this phenotype is caused by deregulation of a group of mitotic regulators because of the absence of RNF4.

Because the effect of RNF4 knockdown and binding assay was most prominent in the fraction of SUMOylated MIS18BP1, the dynamics of the modifications on this important regulator of chromosome segregation were studied in more detail. U2OS cells expressing His_10_-SUMO2 were synchronized using two independent blocking agents into various stages of the cell cycle. After His_10_-pulldown and immunoblot analysis, SUMOylation of MIS18BP1 was observed to increase during late mitosis (Fig. 7*A*), which is represented by the samples released 16 h or 2 to 4 h after respectively thymidine or nocodazole block (supplemental Fig. S6*A*). Upon analysis of samples obtained similarly from cells expressing His_10_-ubiquitin, also the ubiquitylation of MIS18BP1 was shown to be increased in samples enriched for cells in late mitosis (Fig. 7*B* and supplemental Fig. S6*B*). Interestingly, the total protein level of MIS18BP1 was observed to decrease in the input samples directly following the time points showing increased modification levels, namely at 20 h and 8 h after release from respectively thymidine and nocodazole.

The observed decrease in protein stability just after modification by SUMO and ubiquitin indicated that RNF4 regulates MIS18BP1 protein half-life by crosstalk between SUMO and ubiquitin during mitosis. In addition, we hypothesize that many of the other mitotic regulators identified to be co-modified by SUMO and ubiquitin, including KIF23, are regulated by RNF4 during mitosis in a similar fashion (Fig. 7*C*). Finally, group modification by RNF4 of these important mitotic regulators might be essential for proper chromosome segregation (Fig. 7*D*). In the absence of RNF4, crosstalk between SUMO and ubiquitin is blocked, resulting in a stabilization of the SUMOylated form of the identified group of mitotic proteins and subsequently chromosome segregation errors. Thereby, this project did not only discover the extent and complexity of crosstalk among different PTMs, it also revealed a large network of mitotic proteins modified by SUMO and ubiquitin to regulate error-free chromosome segregation.

## DISCUSSION

### 

#### 

##### Improved Methodology Enables Identification of Co-modified Proteins by SUMO and Ubiquitin

Crosstalk among different ubiquitin-like modifiers was previously investigated using single target protein approaches, uncovering PML (12), PML-RARα (13), MDC1 (26–29), HIF2α (30), Tax (31), XPC (32), PARP1 (33), CENPI (34), KDM5B (23, 24), c-Myc (22, 35), and TRIM28 (36) as target proteins. Here, we report on a system-wide proteomic analysis of crosstalk between SUMO and ubiquitin. In this project, we have overcome technical challenges by designing an improved and efficient method to sequentially purify proteins modified simultaneously by two different PTMs. Because this improved strategy can be applied to different combinations of various ubiquitin-like modifiers, it will enable to enhance our knowledge about crosstalk on a PTM-wide level.

For the current project, we focused on the identification of crosstalk between SUMO and ubiquitin involved in proteasomal degradation. Switching the tags on each modifier and thereby performing two independent approaches enabled stringent filtering and more reliable identification of co-modified proteins by mass spectrometry. An interesting significant enrichment for biological processes involved in DNA modification was observed specifically among the co-modified proteins identified upon DMSO treatment, indicating the existence of crosstalk under control conditions and thereby revealing a novel area of potential research. However, the extent of co-modified proteins greatly increased upon inhibition of the proteasome, indicating a prominent role for crosstalk between SUMO and ubiquitin in protein degradation. A set of 498 targets was found to be co-modified upon MG132 treatment, including protein groups involved in transcription, DNA repair and the cell cycle. We identified most targets that are known to be co-modified by SUMO and ubiquitin, including PML, KDM5B, c-Myc and TRIM28, which confirms the reliability of our method. Other known targets, such as PML-RARα and Tax, are not expressed in U2OS cells and could therefore not be identified. Finally, the remaining set of known targets could be only co-modified on specific stimuli of the cell such as DNA damage, hypoxia or enrichment for a specific cell cycle phase.

Co-modification of the identified targets can be the result of various mechanisms, which could subsequently result in different effects on protein function. Our improved method will purify proteins directly modified by both SUMO and ubiquitin as well as proteins modified by a chain consisting of both SUMO and ubiquitin (12). Because the role of these potential mixed chains is mostly unknown, it would be interesting to study their possible involvement in protein signaling. However, our experimental results showed that the abundance of these chains is limited, indicating that most of the identified proteins are actually directly and independently modified by SUMO and ubiquitin via different acceptor lysines in these target proteins.

We decided to focus our search for co-modified targets at the protein level to identify the extent of crosstalk. Additionally, we identified a set of ubiquitin modification sites. However, due to low stoichiometry of co-modified proteins and especially of the specific modified peptides, it is challenging to identify a large set of modification sites on these proteins. Therefore, a less complete overview of the co-modified proteins would probably be identified by focusing on the level of modified peptides. Moreover, the use of wild-type SUMO2 largely precluded the identification of SUMO acceptor lysines (37). A search for diGly motifs by MaxQuant identified 28 peptides modified by ubiquitin that were specifically enriched in the samples containing co-modified proteins (supplemental Table S8). Interestingly, the recent rise in identification of ubiquitylation as well as SUMOylation sites has provided us with a great resource of known modified amino acids in many proteins. By searching both the PhosphoSitePlus database (38) and a recent review which compared all studies identifying SUMOylation sites (37), we found that one or more SUMOylation or ubiquitylation sites were already identified for respectively 68% or 90% of all 498 co-modified proteins enriched upon inhibition of the proteasome (supplemental Table S9). This analysis shows the rich amount of site information that is already available and indicates that most of our identified proteins have previously been confirmed at the site-specific level.

Interestingly, the nature of the ubiquitin-ubiquitin linkages on the co-modified proteins is rather diverse, with virtually all internal ubiquitin lysines involved in chain formation (supplemental Table S8). Surprisingly, we did not detect the lysine 48 ubiquitin linkage, despite being a hallmark for targeting proteins to the proteasome for degradation. However, other ubiquitin-ubiquitin linkages are also involved in targeting proteins to the proteasome (39). Of note, the employed N-terminal tagging of ubiquitin and SUMO does preclude the formation of linear chains.

While our manuscript was in preparation, an alternative approach was published to identify proteins co-modified by SUMO and ubiquitin (40). Thibault and co-workers employed a His_6_-SUMO3 purification and a sequential ubiquitin remnant immunoaffinity purification from lysates of HEK293 cells treated with the proteasome inhibitor MG132. The flow-through of the ubiquitin remnant immunoaffinity purification was subjected to SUMO remnant immunoaffinity purification. They found a total of 194 proteins containing ubiquitylation sites in their pool of His_6_-SUMO-enriched proteins. However, for 113 of these proteins no SUMOylation sites were identified. The authors hypothesize that these 113 proteins could represent proteins modified by SUMO on their conjugated ubiquitin chains. Our experiments revealed that although mixed SUMO-ubiquitin chains are formed, most of co-modified proteins contain SUMO and ubiquitin on separate lysine residues of the target proteins in U2OS cells. Whether the limited role of mixed SUMO-ubiquitin chains also holds true for other cell types, including HEK293 cells, remains to be addressed. Additionally, the identification of ubiquitylated proteins without SUMOylation sites could represent background binders due to the limited stringency of the His_6_-approach. The identification of ubiquitylation and SUMOylation sites on 81 targets is an impressive step forward into the technically challenging direction of studying crosstalk on a site-specific level. By focusing on the protein level and employing a His_10_-approach, we have identified a substantial larger pool of 498 co-modified proteins by SUMO and ubiquitin upon inhibition of the proteasome. Therefore, we believe that both approaches complement each other and provide us with state-of-the-art knowledge on crosstalk between SUMO and ubiquitin on the sites as well as protein level.

##### Identification of Functionally Distinct Protein Groups Among the Co-modified Targets

Interestingly, we identified an enrichment for proteins involved in the biological process of protein modification itself among the targets co-modified by SUMO and ubiquitin, showing the great extent of interconnectivity among different modifications. We were expecting to identify various SUMO E3 ligases to be co-modified on proteasomal inhibition, due to their auto-SUMOylation and the role of ubiquitylation in their degradation. However, the great extent of identified PTM machinery, not only involved in the process of SUMOylation but also in many other modifications including ubiquitylation, phosphorylation, methylation and acetylation, let us to believe that we have revealed a complex network that is regulated by PTM crosstalk. Future studies will have to unravel this extra layer of crosstalk and will potentially identify additional layers by other PTM combinations, revealing the interesting phenomenon of modified modifiers.

In addition to regulating PTM machinery, our results show that crosstalk can regulate many other proteins involved in a wide variety of gene ontology annotated biological processes. These target proteins can be co-modified by various crosstalk mechanisms. The best studied mechanism of crosstalk between SUMO and ubiquitin is modification by SUMO-targeted ubiquitin ligases. Two STUbL enzymes are currently known in humans, namely RNF4 and RNF111, which can recognize SUMOylated target proteins and subsequently ubiquitylate them. Upon inhibition of the proteasome, these co-modified proteins are not able to be degraded and could therefore be identified by mass spectrometry upon enrichment using our improved strategy. Because this is currently the main mechanism of crosstalk between SUMO and ubiquitin involved in proteasomal degradation, we are expecting a large fraction of our identified proteins to be STUbL targets. Interestingly, not all proteins identified to be co-modified upon inhibition of the proteasome have to be targets of RNF4 or RNF111. Given the wealth of ubiquitin E3 ligases, additional STUbLs might be identified.

##### Regulation of Mitotic Proteins Via RNF4 During Cell Cycle Progression

Various functionally different protein groups were found among the co-modified targets, including many mitotic regulators. Interestingly, gene ontology analysis revealed significant enrichment of proteins involved in the specific process of chromosome segregation. Because knockdown of RNF4 is known to result in chromosome segregation errors (25), we were wondering whether this co-modified group could be novel targets of the STUbL and thereby explain the observed phenotype. The co-modification of two mitotic regulators was verified and both KIF23 and MIS18BP1 were indeed identified as novel RNF4 targets. In addition, the modification dynamics of MIS18BP1 showed an increase during late mitosis, with equal timing for its SUMOylated as well as its ubiquitylated fraction. These results indicate that MIS18BP1 is regulated by crosstalk between SUMO and ubiquitin via RNF4 during mitosis, which results in its degradation by the proteasome. The latter hypothesis was confirmed by the observation that total MIS18BP1 protein levels were decreased in the first time-points after the increase in modifications was observed. These results suggest that SUMOylated MIS18BP1 is recognized by RNF4, which results in its ubiquitylation and subsequent degradation during mitosis.

Given the important role of RNF4 in chromosome segregation, this STUbL could coregulate a group of targets relevant for this process. By regulating the half-life of this set of proteins, RNF4 uses its group modification ability and thereby has become an essential player in chromosome segregation. Upon knockdown of RNF4, these proteins are not degraded at the right time during mitosis, resulting in their stabilization and consequently, chromosome segregation errors. Interestingly, similar problems during chromosome segregation were observed upon knockdown of the SUMO E3 ligase PIAS4 (41), indicating a potential collaboration between PIAS4 and RNF4 during the process of chromosome segregation. According to our hypothesis, knockdown of PIAS4 would prevent SUMOylation of the identified protein group and thereby inhibit their recognition by RNF4, which would prevent their degradation and therefore result in a similar phenotype as was observed for the knockdown of RNF4 itself. This hypothesis needs to be verified, which could lead to more detailed knowledge about this crosstalk between SUMO and ubiquitin and its role in mitotic regulation.

##### Concluding Remarks

We have developed improved methodology that will help to increase our knowledge about crosstalk among various PTMs. By applying this improved methodology, proteins co-modified by SUMO and ubiquitin upon inhibition of the proteasome were identified in a system-wide manner. Interestingly, this revealed the co-modification of important mitotic regulators, including a subset involved in chromosome segregation. Subsequently, MIS18BP1 was identified as a novel RNF4 target, which regulates its stability during mitosis. We hypothesize that RNF4 is a key mitotic regulator for the identified group of proteins, including MIS18BP1, thereby timing their destabilization during chromosome segregation. Finally, the application of this improved method has revealed the extent of interconnectivity among different PTMs and will help to increase our knowledge and discover more details about crosstalk among protein modifications in the future.

## DATA AVAILABILITY

The mass spectrometry data have been deposited to the ProteomeXchange Consortium via the PRIDE (42) partner repository with the data set identifier PXD007733.

## Supplementary Material

Supplemental Data

## Footnotes

^1^ The abbreviations used are:

PTM: post-translational modification
ABC: ammonium bicarbonate
ACN: acetonitrile
CAA: chloroacetamide
CD: catalytic domain
DMEM: dulbecco's modified eagle's medium
DMSO: dimethyl sulphoxide
DTT: dithiothreitol
FA: formic acid
FC: fold change
FCS: fetal calf serum
GOBP: gene ontology based biological process
HEK293T: human embryonic kidney 293 cell line
IP: immunoprecipitation
LDS: Lithium Dodecyl Sulfate
NanoLC-MS/MS: nanoflow liquid chromatography-tandem mass spectrometry
P/S: penicillin/streptomycin
PBS/T: PBS with 0.05% Tween-20
PD: pulldown
PEI: polyethyleneimine
RT: room temperature
SIM: SUMO interaction motif
STUbL: SUMO-targeted ubiquitin ligase
SUMO: small ubiquitin-like modifier
TFA: trifluoroacetic acid
U2OS: human bone osteosarcoma cell line.
