# Supplementary material for: Converging Small Ubiquitin-like Modifier (SUMO) and Ubiquitin Signaling: Improved Methodology Identifies Co-modified Target Proteins
Source: Mol Cell Proteomics. 2017 Sep 26;16(12):2281–95. doi: 10.1074/mcp.TIR117.000152 (PMC5724187; doi:10.1074/mcp.TIR117.000152)
Supplement: Supplemental Data [file supp_16_12_2281__index.html]

Converging SUMO and ubiquitin signaling: improved methodology identifies co-modified target proteins — Converging SUMO and ubiquitin signaling — Converging Small Ubiquitin-like Modifier (SUMO) and Ubiquitin Signaling: Improved Methodology Identifies Co-modified Target Proteins — Converging SUMO and Ubiquitin Signaling — Supplemental Data 

# Converging Small Ubiquitin-like Modifier (SUMO) and Ubiquitin Signaling: Improved Methodology Identifies Co-modified Target Proteins

## Supplemental Data

- PDF\_S1 - MS/MS Spectra
- Supplement - Supplemental methods, results and figures
- Table S3 - All identified diGly (K) sites. A list of all peptides identified by MaxQuant to be modified by a diGly motif on the RAW GlyGly (K) Sites sheet. The second sheet explains which sample names belongs to each exact sample measured.
- Table S4 - Co-modified targets under control conditions and upon inhibition of the proteasome identified by overlapping both approaches. Proteins with a q value below 0.03 for both approaches upon MG132 treatment can be found on the co-modified targets upon MG132 sheet, while those identified under DMSO conditions are shown on the co-modified targets upon DMSO sheet. For both lists, additional columns are included summarizing both approaches into one q value (- log10(x\*y)) and one difference value ((x+y)/2) used for shape size and color in the visualized networks. The third sheet explains which sample names belongs to each exact sample measured.
- Table S5 - Co-modified targets under control conditions and upon inhibition of the proteasome identified by pooling both approaches. Proteins with a q value below 0.03 amongst the pooled samples upon MG132 treatment can be found on the Co-modified upon MG132 pooled sheet, while those identified under DMSO conditions are shown on the Co-modified targets upon DMSO pooled sheet. The third sheet explains which sample names belongs to each exact sample measured.
- Table S6 - Enrichment analysis of gene ontology based biological processes. For each biological process the enrichment factor, FDR and -Log10(FDR) obtained from Fisher Exact testing amongst the co-modified targets upon MG132 are shown on the GOBP enrichment targets MG132 sheet, while those obtained from testing amongst the co-modified targets upon DMSO are shown on the GOBP enrichment targets DMSO sheet. On the third sheet all biological processes tested amongst the comodified targets obtained from the pooled approach analysis are shown for the DMSO and MG132 conditions. Rows marked in orange represent the biological processes that were significantly enriched amongst the co-modified targets upon DMSO (with a FDR value below 0.03), while not showing a significant enrichment amongst the targets upon inhibition of the proteasome.
- Table S7 - Identification of the co-modified proteins belonging to each selected biological process. For each co-modified target upon MG132 treatment, their gene ontology annotated biological processes were studied and proteins annotated to the selected processes were marker by a plus sign.
- Table S8 - DiGly sites enriched amongst co-modified target proteins. A list of peptides modified by a diGly motif enriched in the samples purified for proteins modified by both SUMO and ubiquitin. For each peptide, the location of the diGly motif within the protein is shown as well as the best localization spectrum evidence ID, which corresponds to the spectra shown in PDF S1.
- Table S9 - Co-modified targets and their published sites. A list of the proteins identified to be significantly co-modified by SUMO and ubiquitin, marked with a plus sign if one or more SUMOylation or ubiquitylation sites are published.
- Table S1 - All identified peptides. A list of all peptides identified by MaxQuant on the RAW Peptides sheet. The second sheet explains which sample names belongs to each exact sample measured.
- Table S2 - All identified protein groups. A list of all protein groups for which peptides were identified by MaxQuant on the RAW ProteinGroups sheet. The second sheet explains which sample names belongs to each exact sample measured.
