# Supplementary material for: Converging Small Ubiquitin-like Modifier (SUMO) and Ubiquitin Signaling: Improved Methodology Identifies Co-modified Target Proteins
Source: Mol Cell Proteomics. 2017 Sep 26;16(12):2281–95. doi: 10.1074/mcp.TIR117.000152 (PMC5724187; doi:10.1074/mcp.TIR117.000152)

Raw file	Spectrum Evidence ID 3894	Scan	Method	Score	m/z	Gene names
QE_SC_double_US_U2OS12221099_MG132_BR1_TR3		2914	FTMS; HCD	104.22	419.55	SUMO2

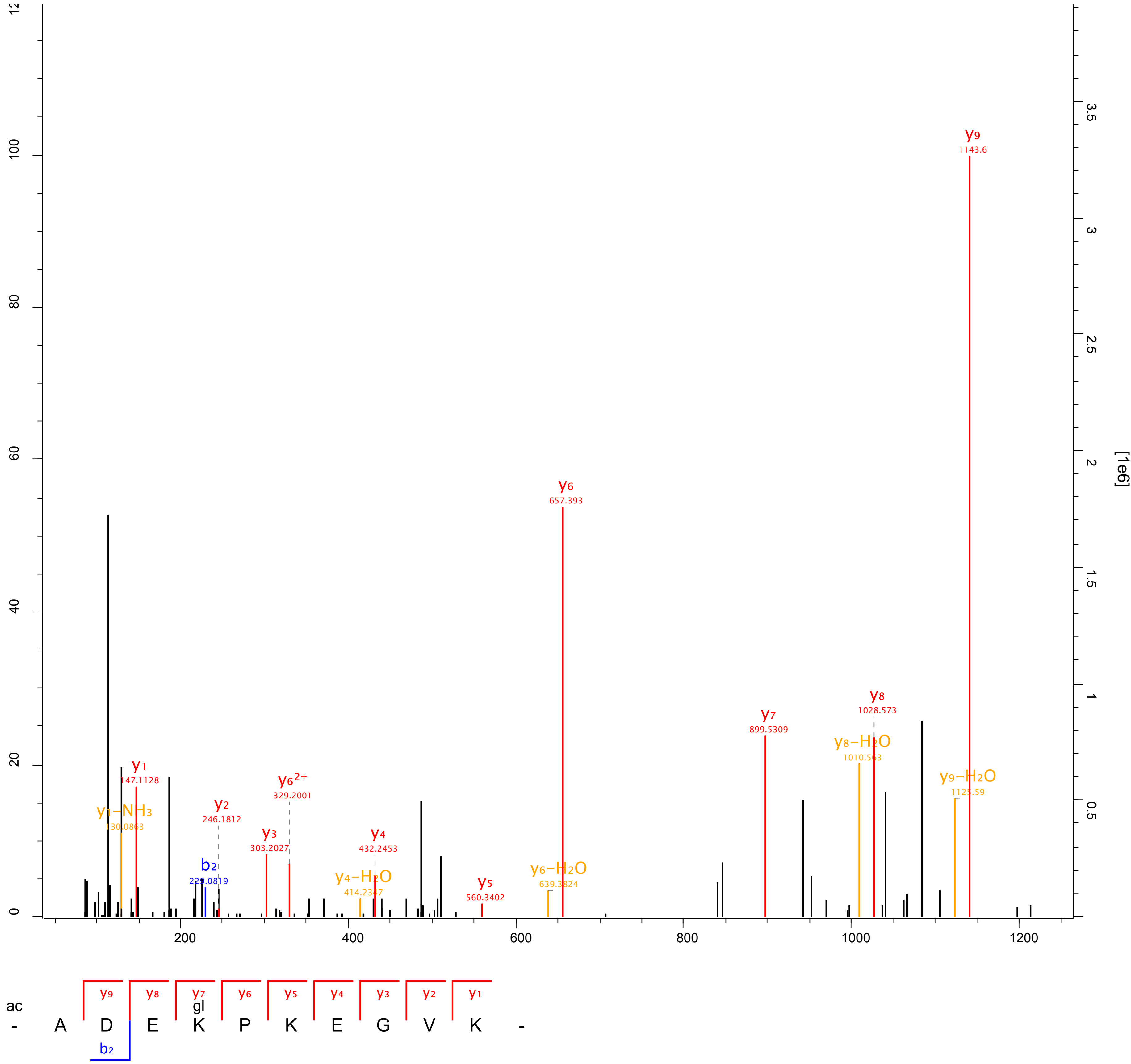

Raw file Spectrum Evidence ID 4060	Scan	Method	Score	m/z	Gene names
QE_SC_double_US_U2OS12221099_MG132_BR1_TR1	4871	FTMS; HCD	66.64	609.81	SUMO2

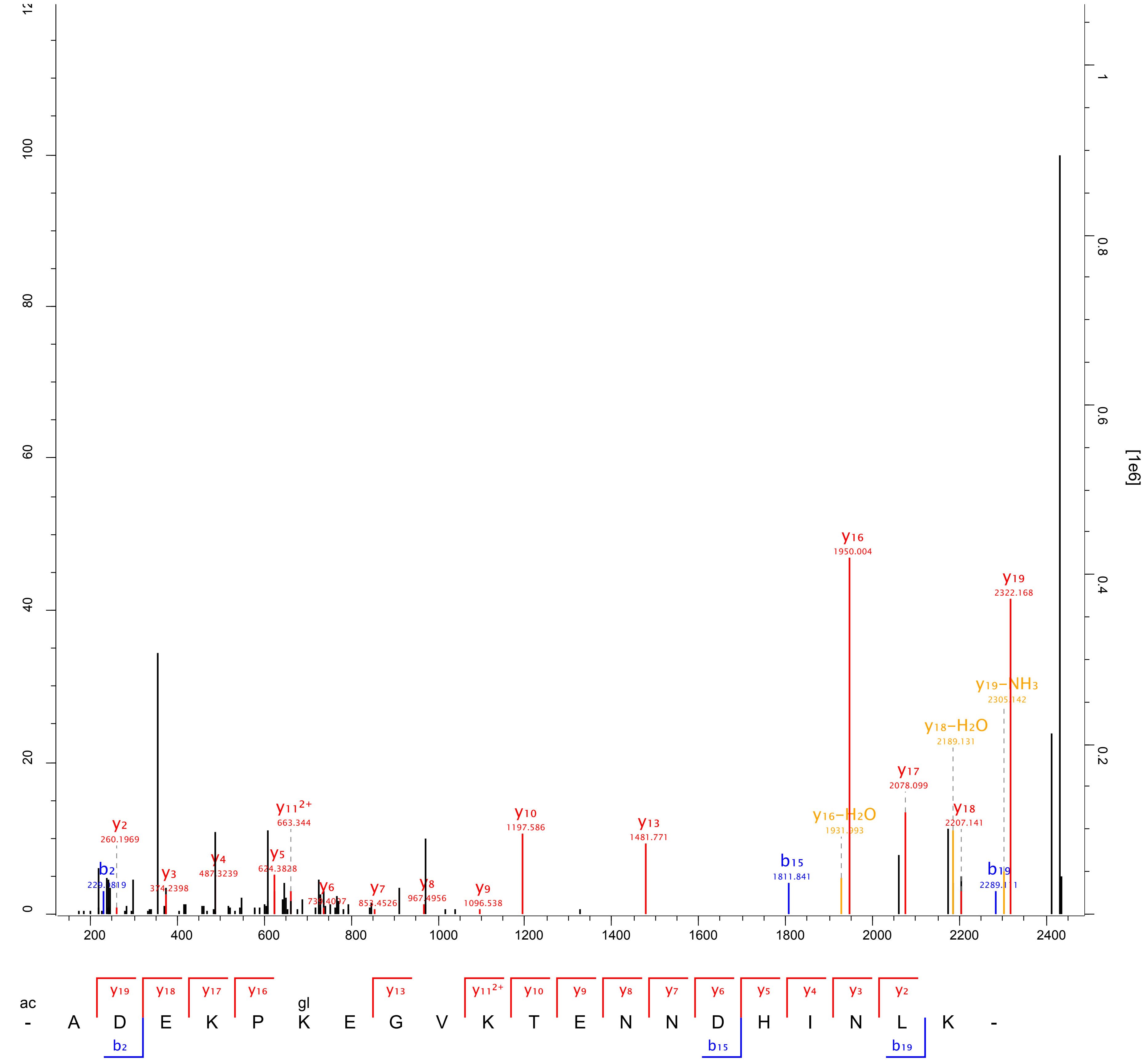

Raw file	Spectrum Evidence ID 16243	Scan	Method	Score	m/z	Gene names
QE_SC_double_US_U2OS12221099_MG132_BR1_TR3		4134	FTMS; HCD	112.68	613.32	UBB;UBC;RPS27A;UBA52

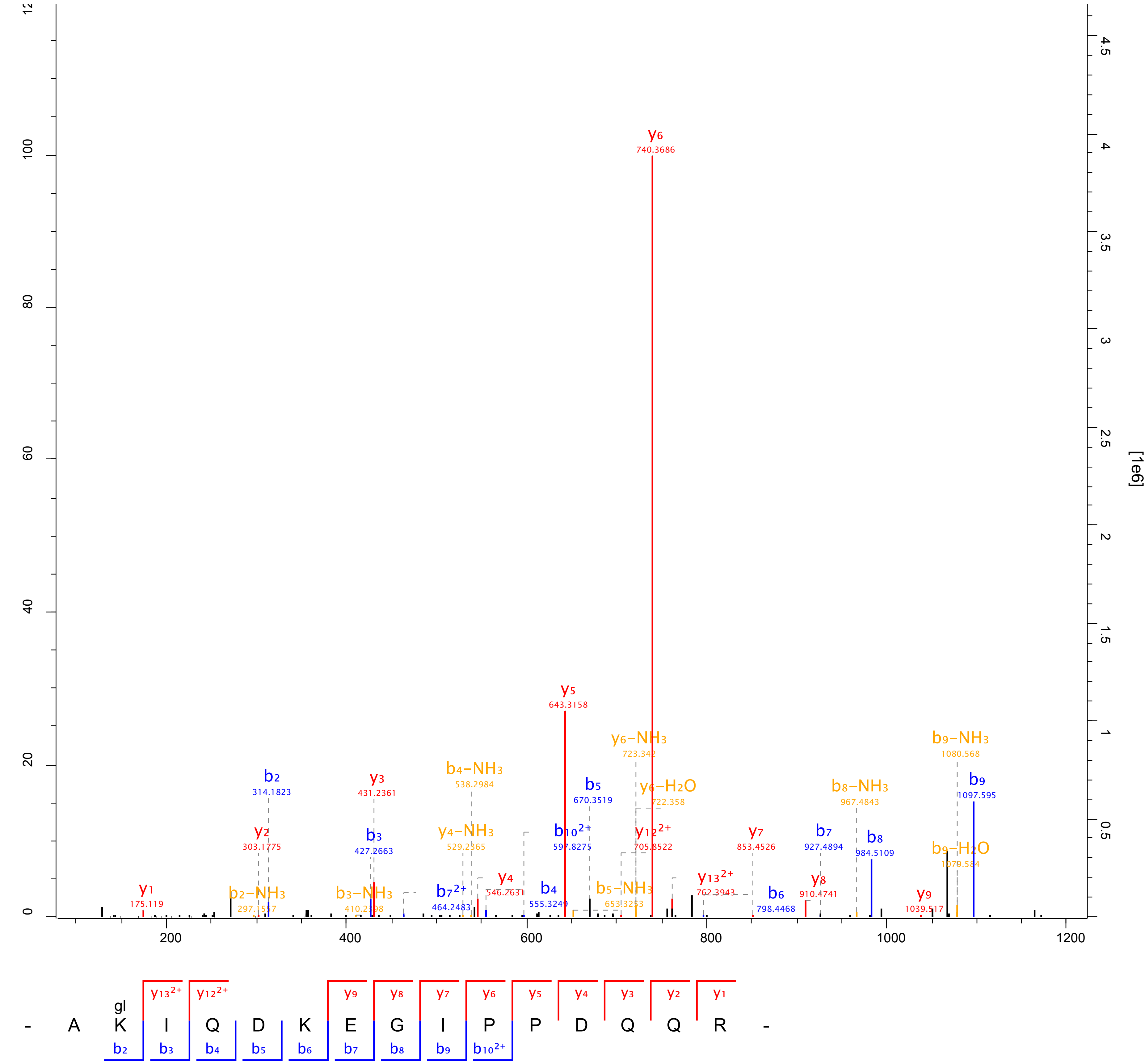

Raw file	Spectrum	Evidence ID	Scan	Method	Score	m/z	Gene names
QE_SC_double_US_U2OS12221099_MG132_BR1_TR3		49305	4035	FTMS; HCD	54.77	489.57	ACTB;ACTG1;ACTA1;ACTC1;ACTG2;ACTA2

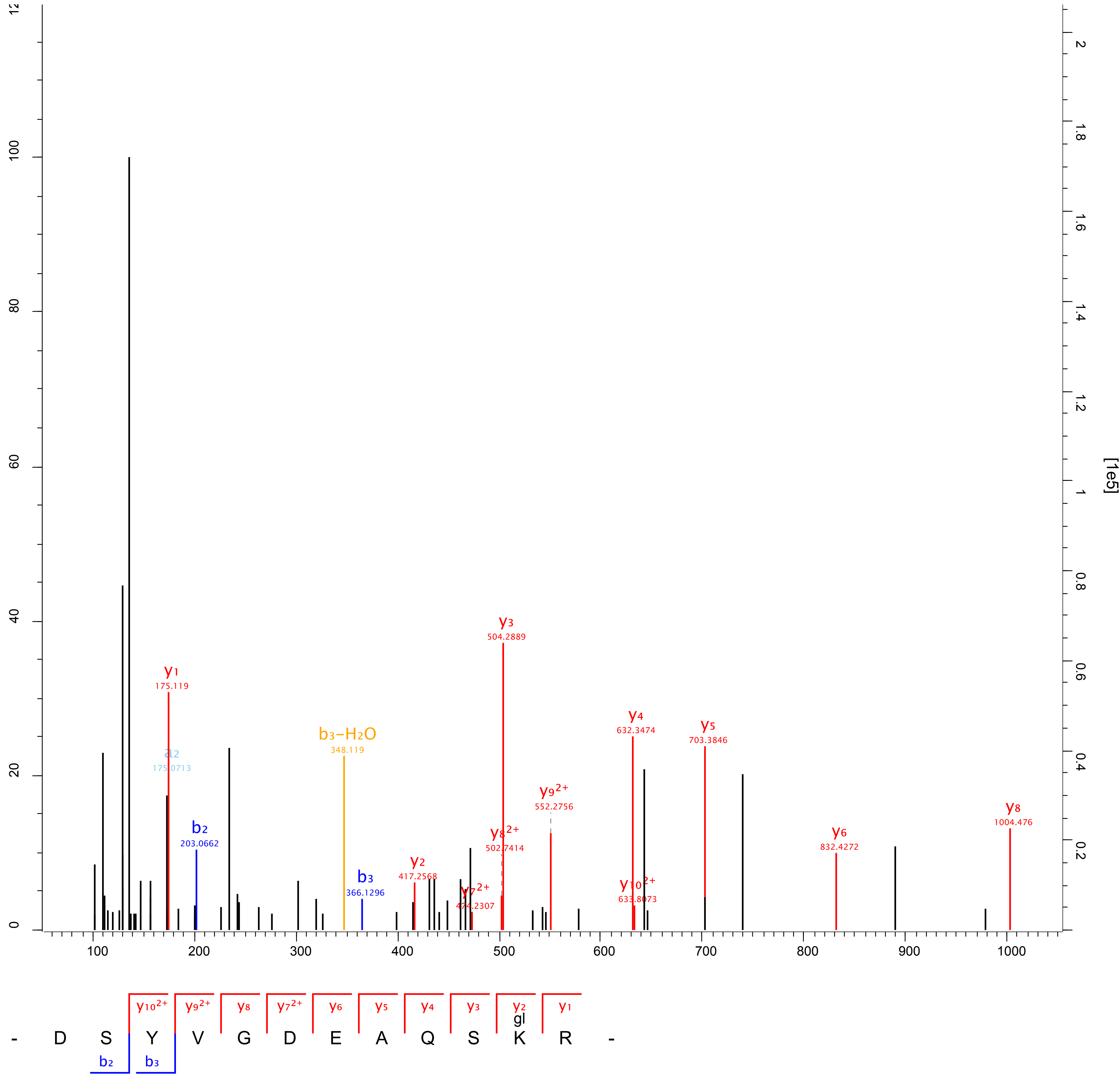

Raw file Spectrum Evidence ID 59527	Scan	Method	Score	m/z	Gene names
QE_SC_double_US_U2OS12221099_MG132_BR2_TR3	4604	FTMS; HCD	86.77	403.46	SUMO3

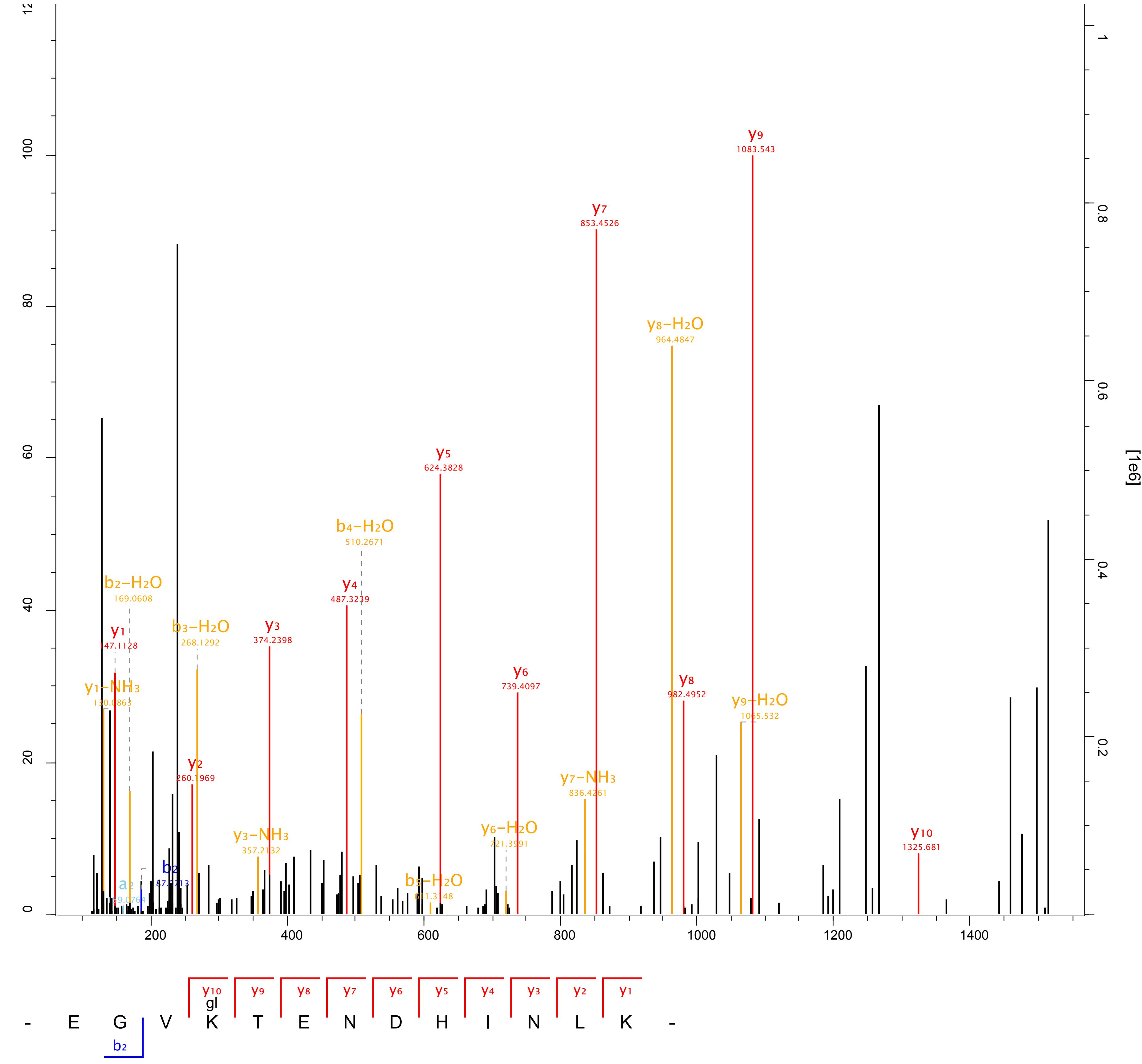

Raw file	Spectrum	Evidence ID 59729	Scan	Method	Score	m/z	Gene names
QE_SC_double_SU_U2OS12221099_MG132_BR5_TR3			4438	FTMS; HCD	64.1	575.62	SUMO2

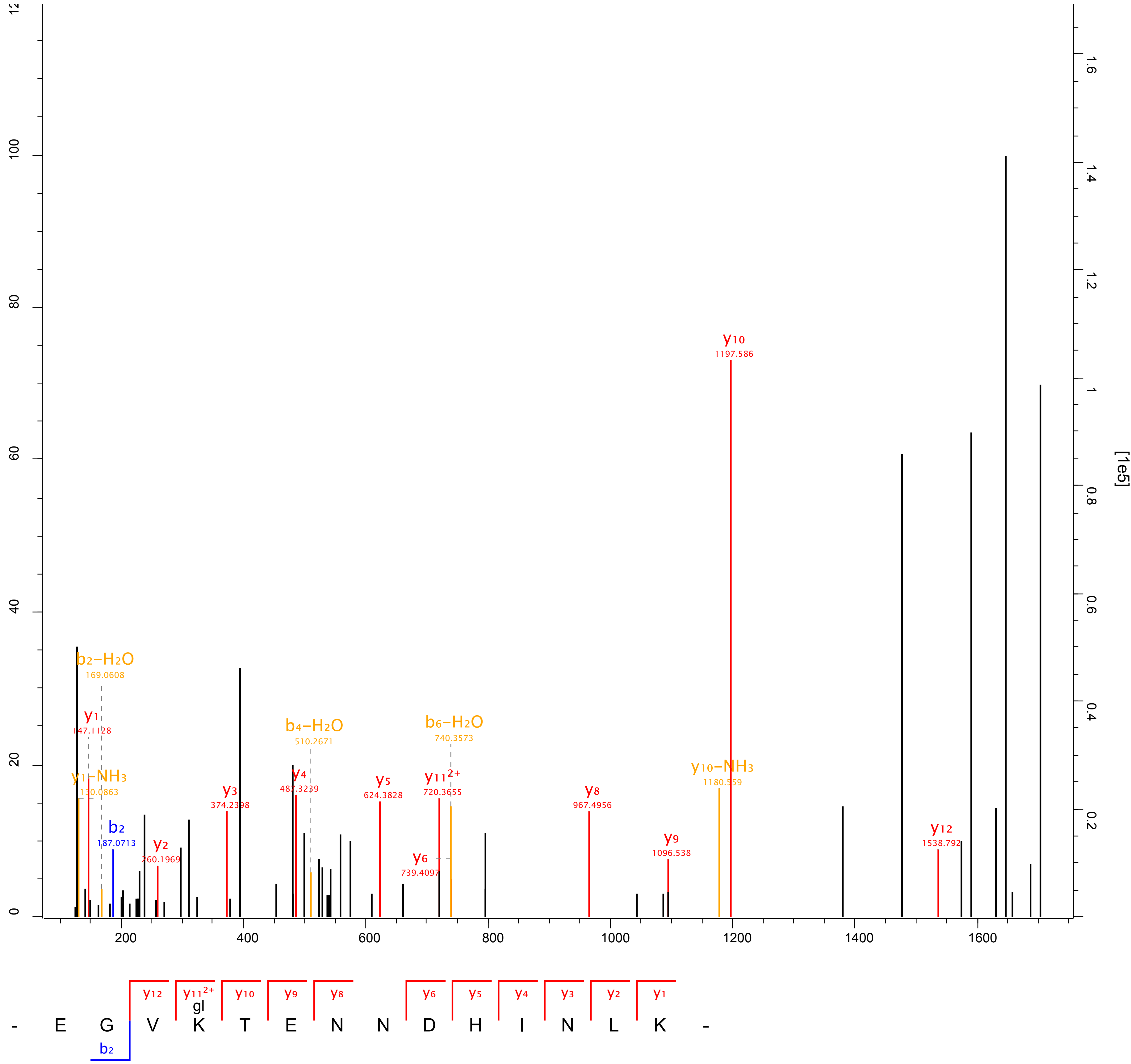

Raw file	Spectrum Evidence ID 78760	Scan	Method	Score	m/z	Gene names
QE_SC_double_US_U2OS12221099_MG132_BR1_TR3		14540	FTMS; HCD	57.43	490.78	RPL15

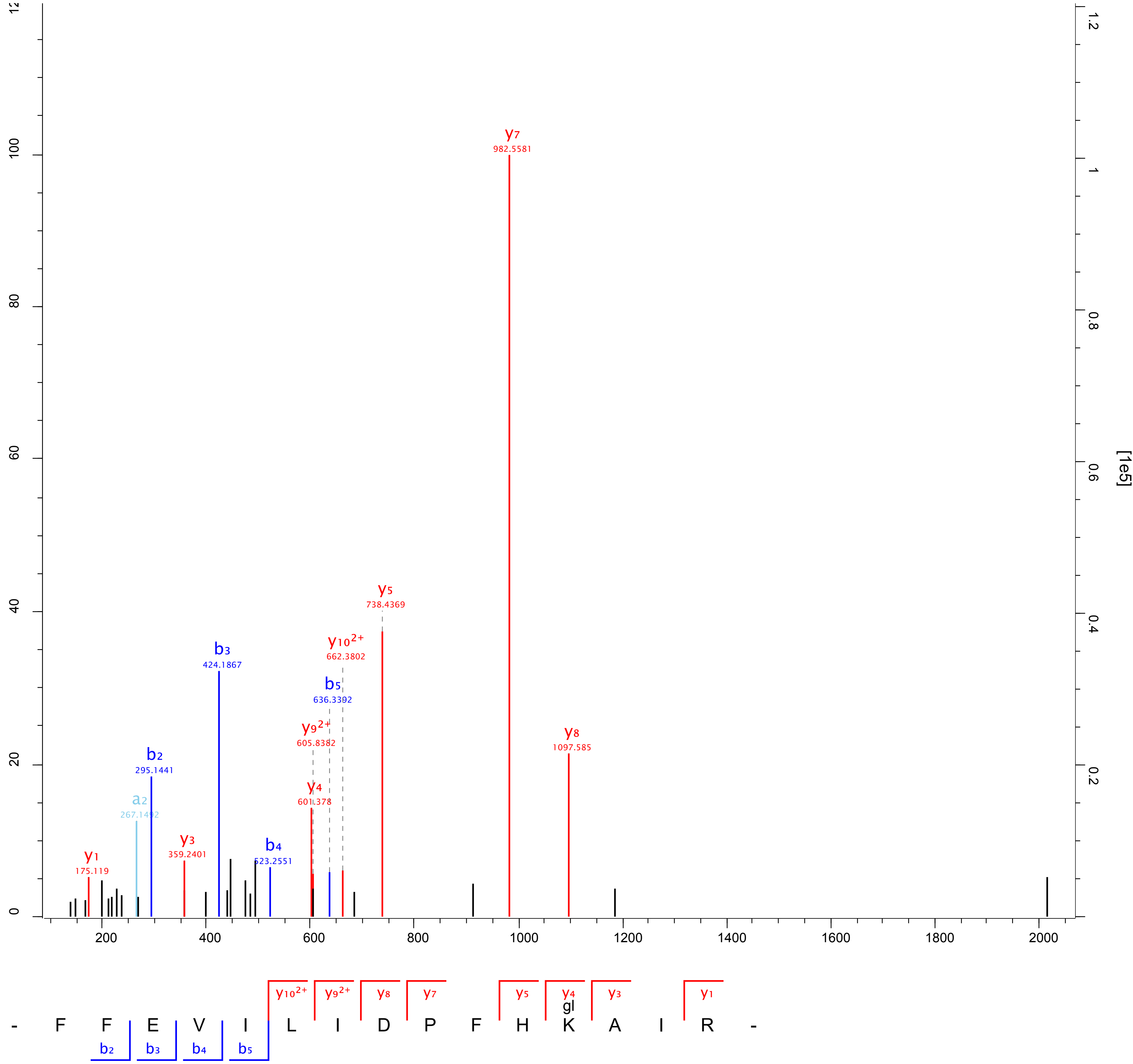

Raw file	Spectrum Evidence ID 115715	Scan	Method	Score	m/z	Gene names
QE_SC_double_US_U2OS12221099_DMSO_BR4_TR3		3936	FTMS; HCD	55.34	473.75	H2BFS;HIST1H2BK;HIST1H2BJ

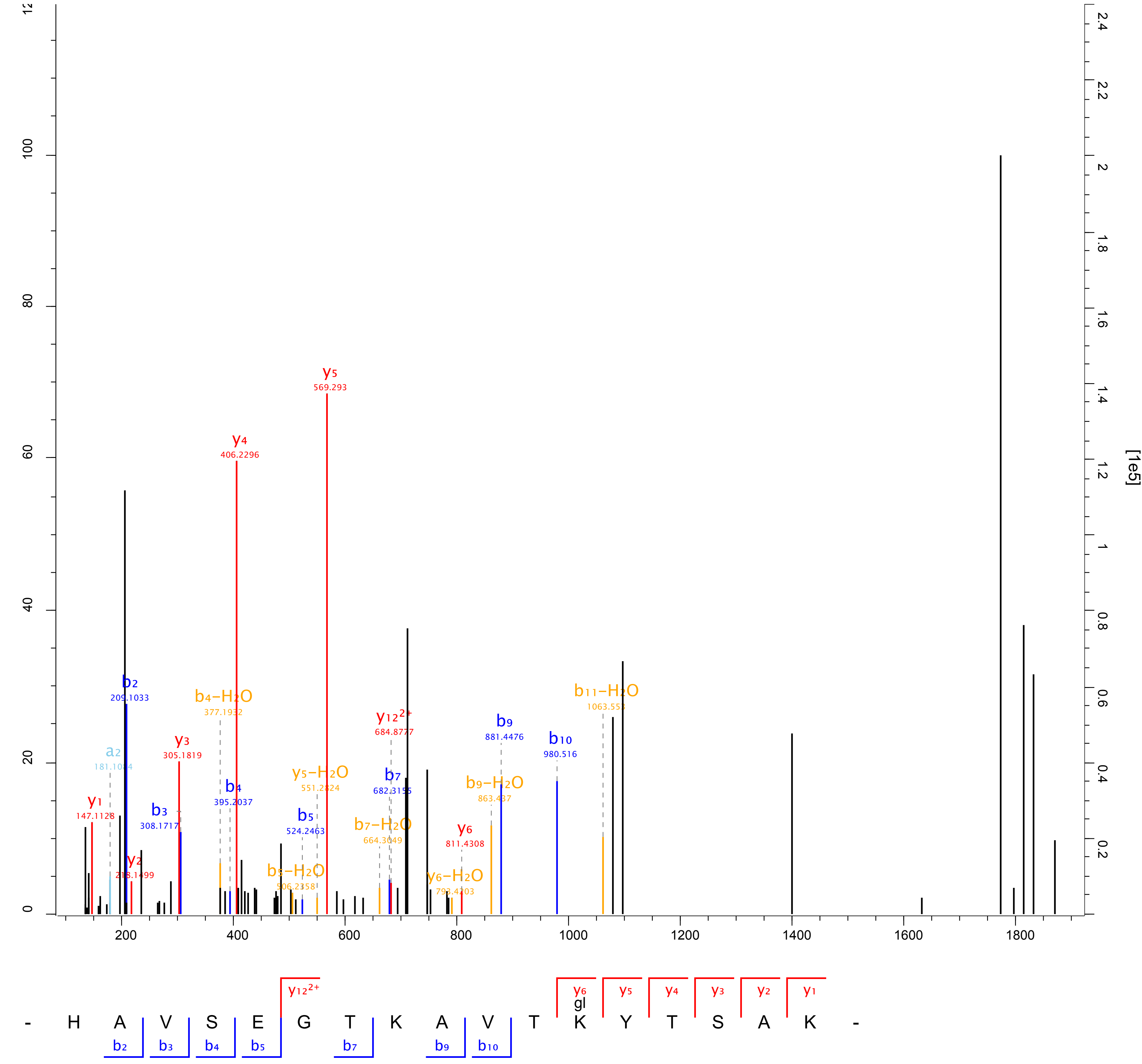

Raw file	Spectrum Evidence ID 129254	Scan	Method	Score	m/z	Gene names
QE_SC_double_SU_U2OS12221099_MG132_BR1_TR1		14295	FTMS; HCD	69.26	658	RNF216

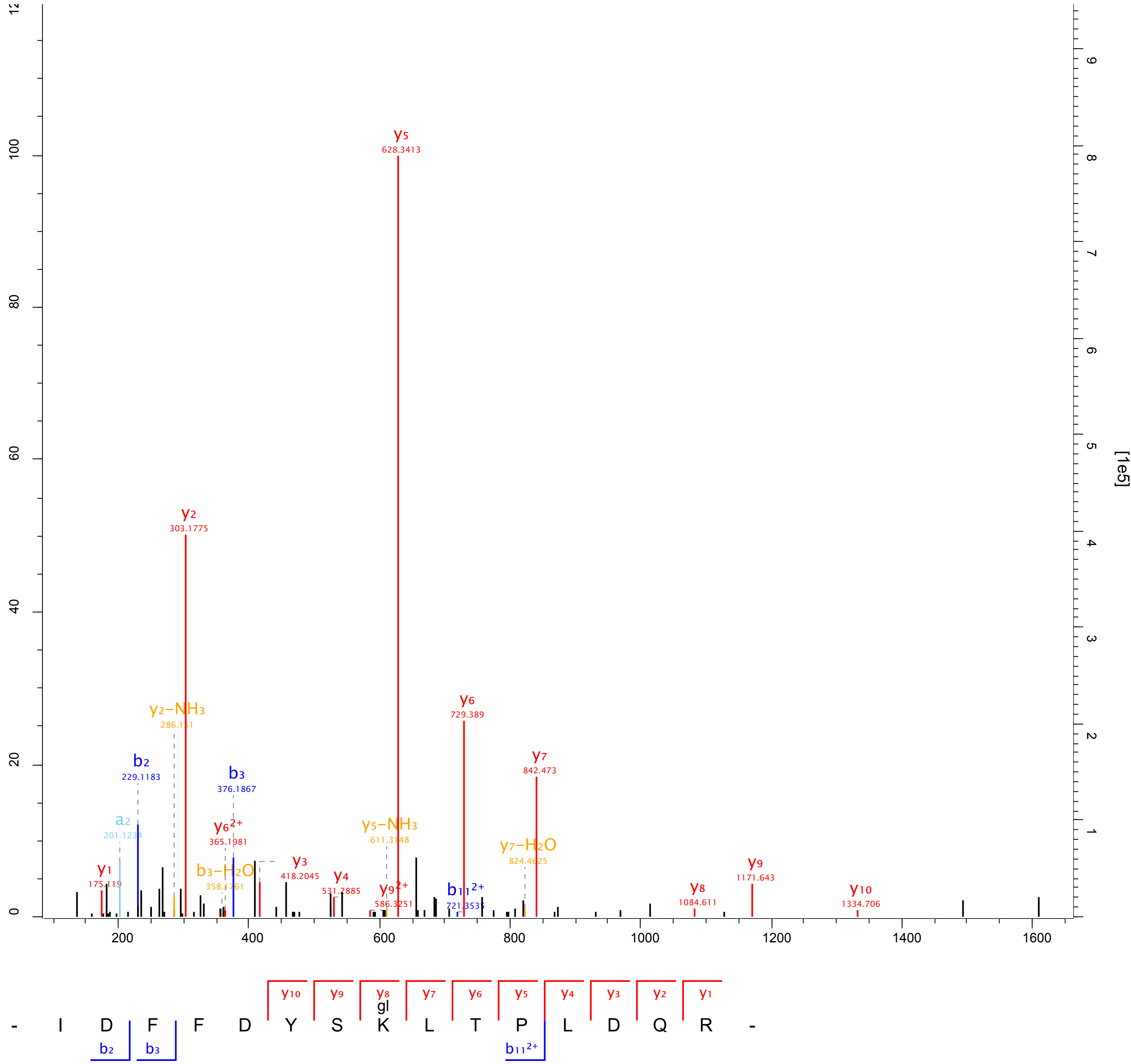

Raw file	Spectrum Evidence ID 136991	Scan	Method	Score	m/z	Gene names
QE_SC_double_SU_U2OS12221099_MG132_BR1_TR1		11663	FTMS; HCD	82.61	757.09	RNF216

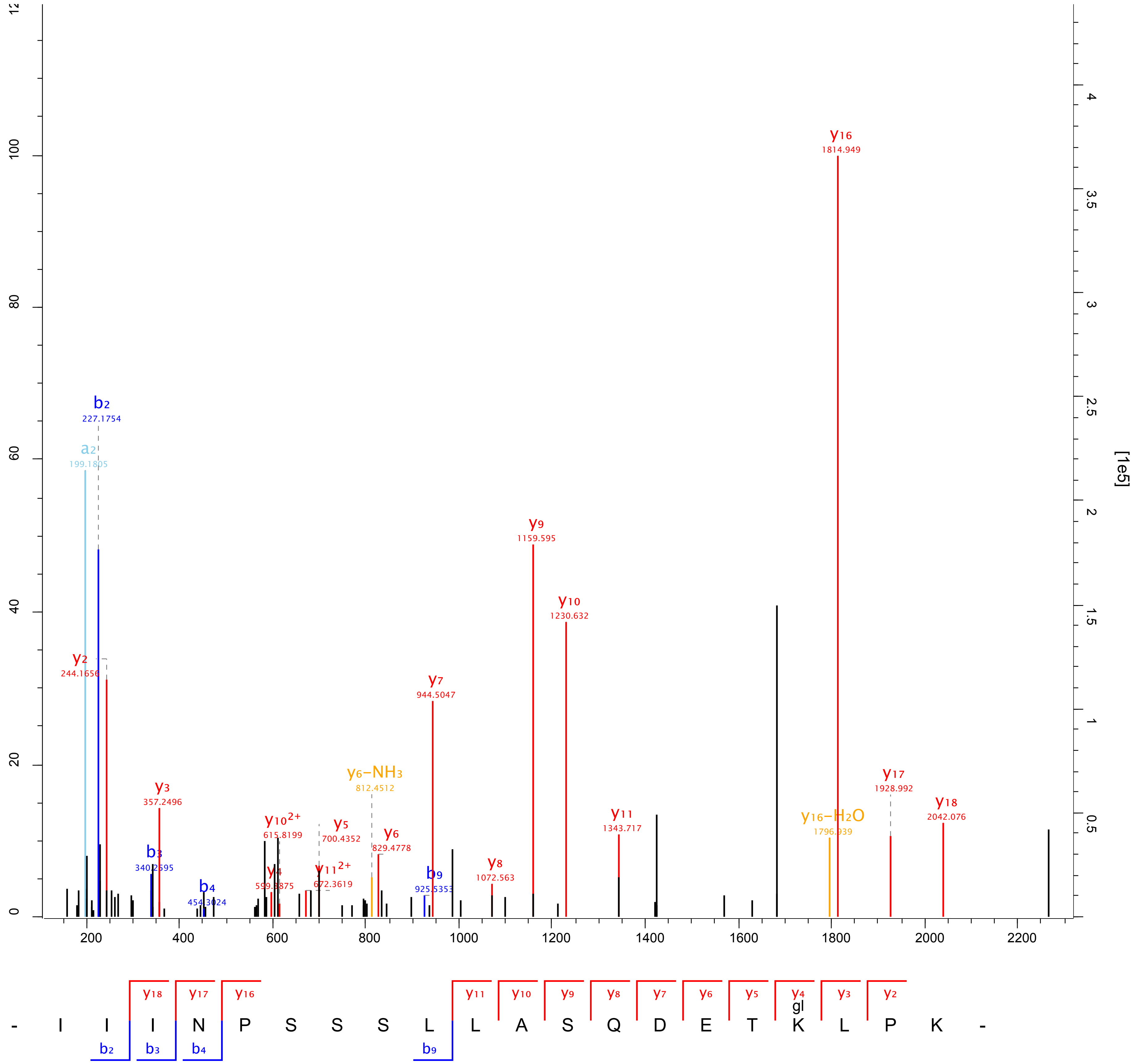

Raw file	Spectrum Evidence ID 142883	Scan	Method	Score	m/z	Gene names
QE_SC_double_US_U2OS12221099_MG132_BR1_TR3		8774	FTMS; HCD	87.57	462.27	HNRNPM

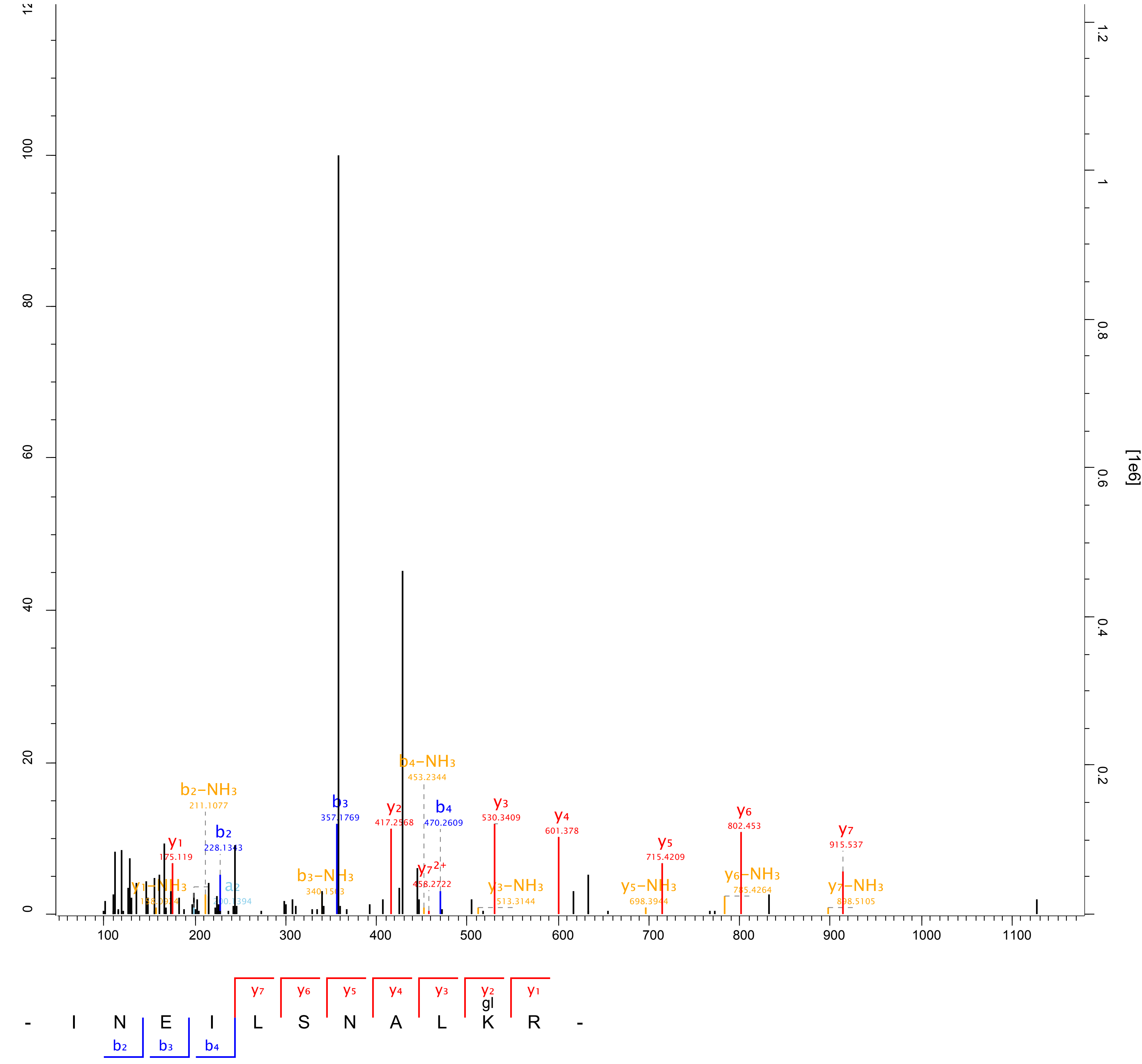

Raw file	Spectrum Evidence ID 144753	Scan	Method	Score	m/z	Gene names
QE_SC_double_US_U2OS12221099_MG132_BR4_TR1		3496	FTMS; HCD	57.11	546.61	UBB;UBC;RPS27A;UBA52

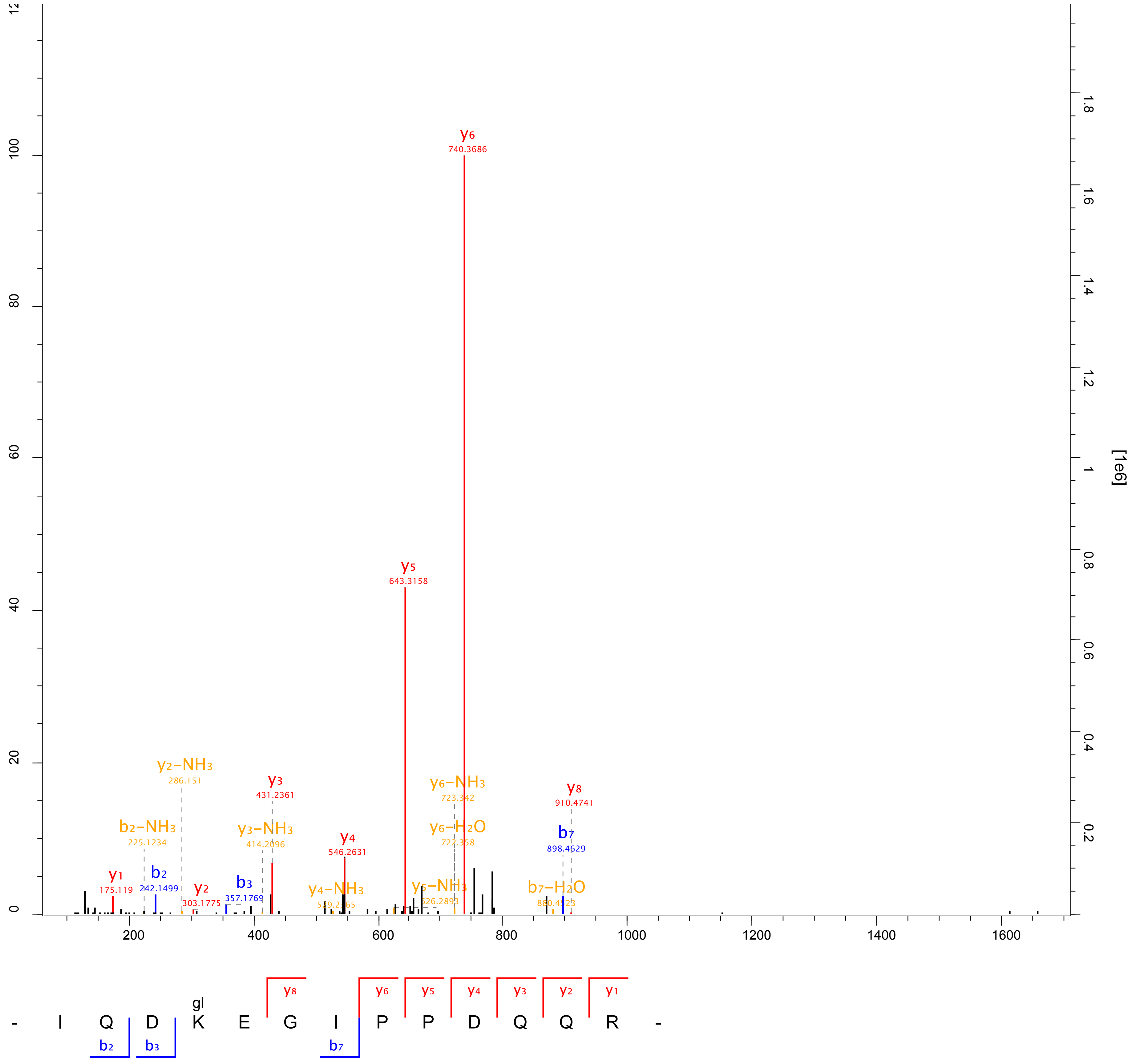

Raw file	Spectrum Evidence ID 153405	Scan	Method	Score	m/z	Gene names
QE_SC_double_SU_U2OS12221099_DMSO_BR3_TR2		4714	FTMS; HCD	73.5	692.88	NIPSNAP1

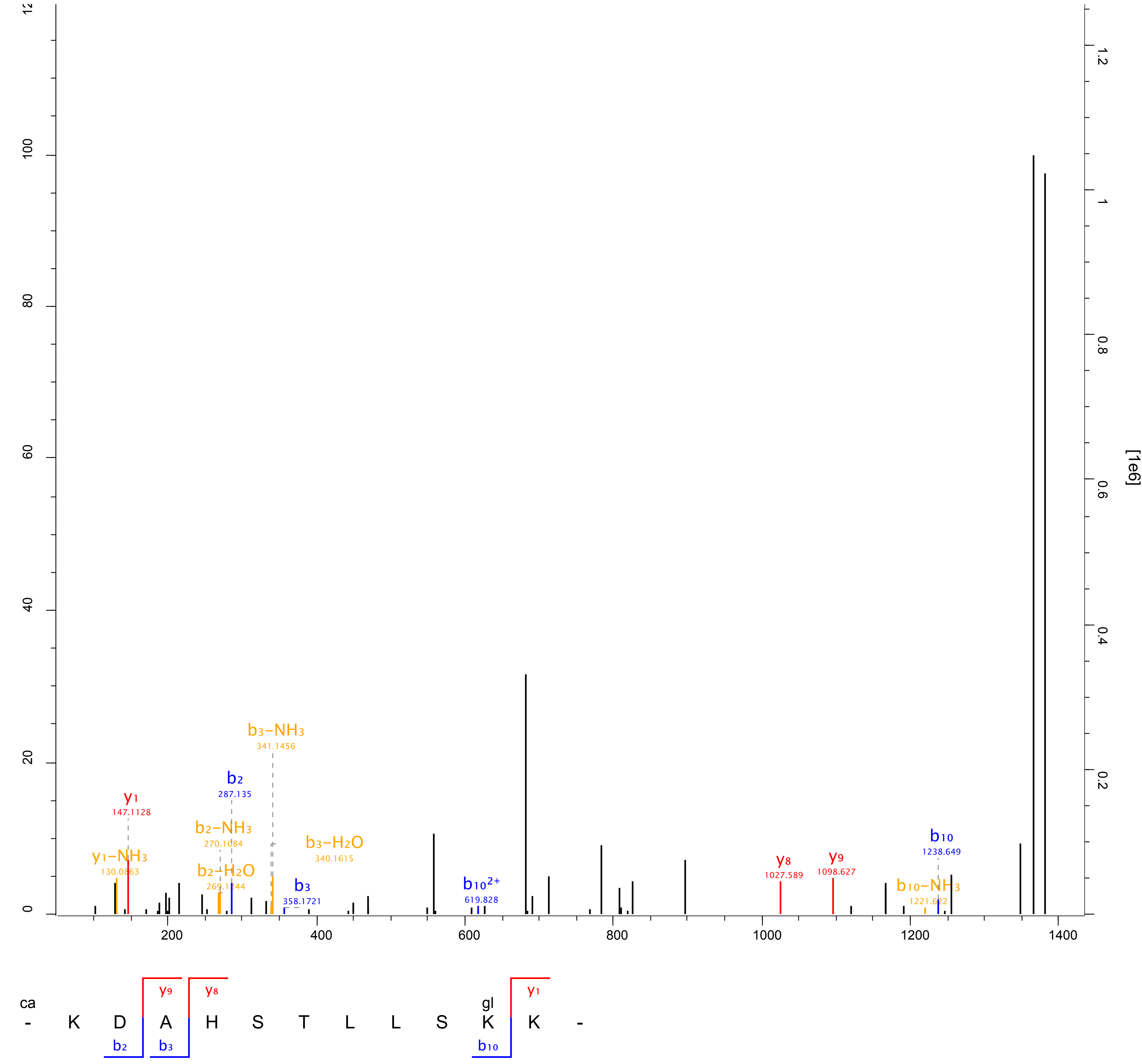

Raw file	Spectrum	Evidence ID 156790	Scan	Method	Score	m/z	Gene names
QE_SC_double_SU_U2OS12221099_MG132_BR3_TR2			5076	FTMS; HCD	100.25	749.88	LIPT1

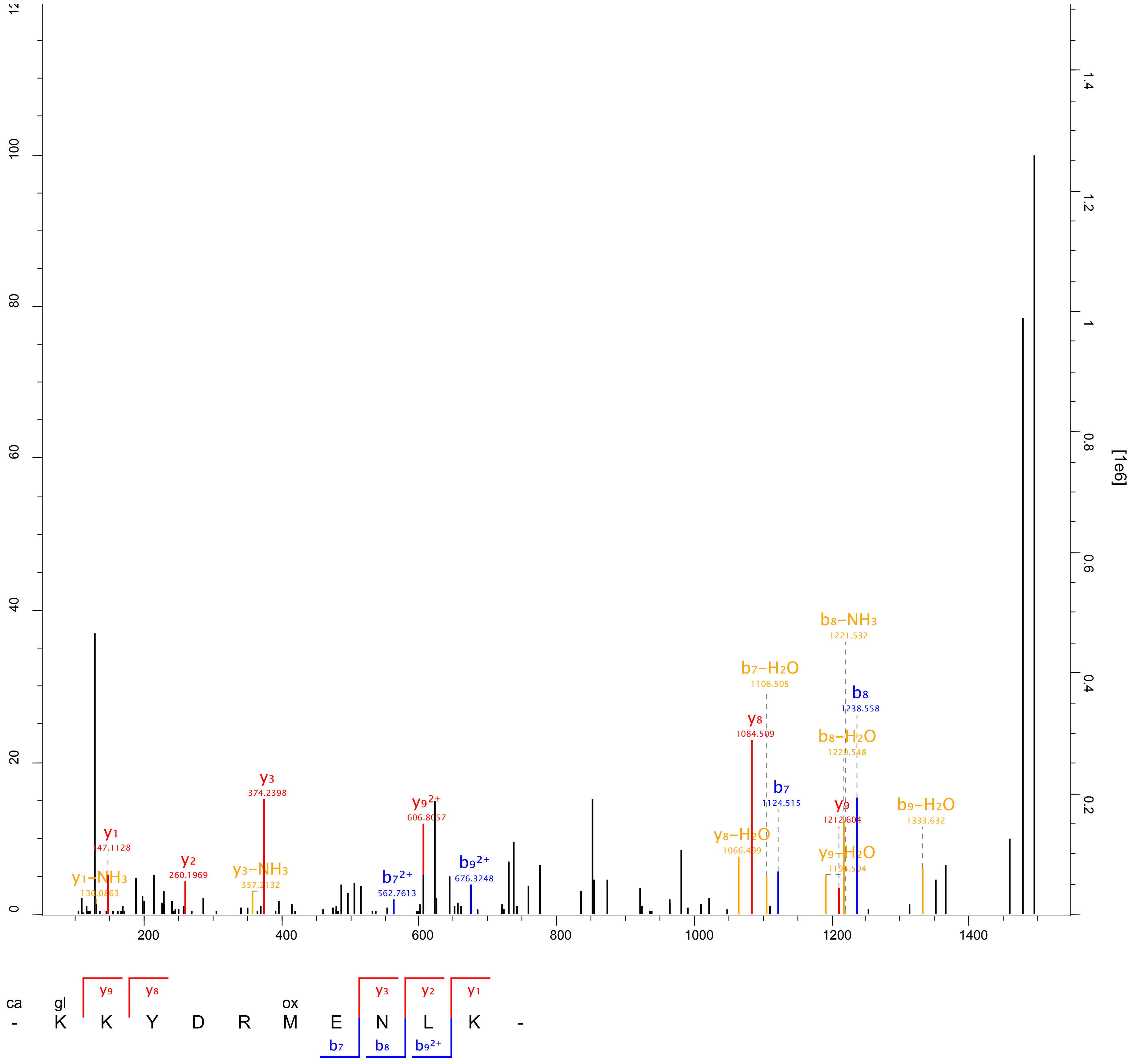

Raw file Spectrum Evidence ID 161808	Scan	Method	Score	m/z	Gene names
QE_SC_double_SU_U2OS12221099_DMSO_BR3_TR3	4389	FTMS; HCD	70.94	499.59	MURC

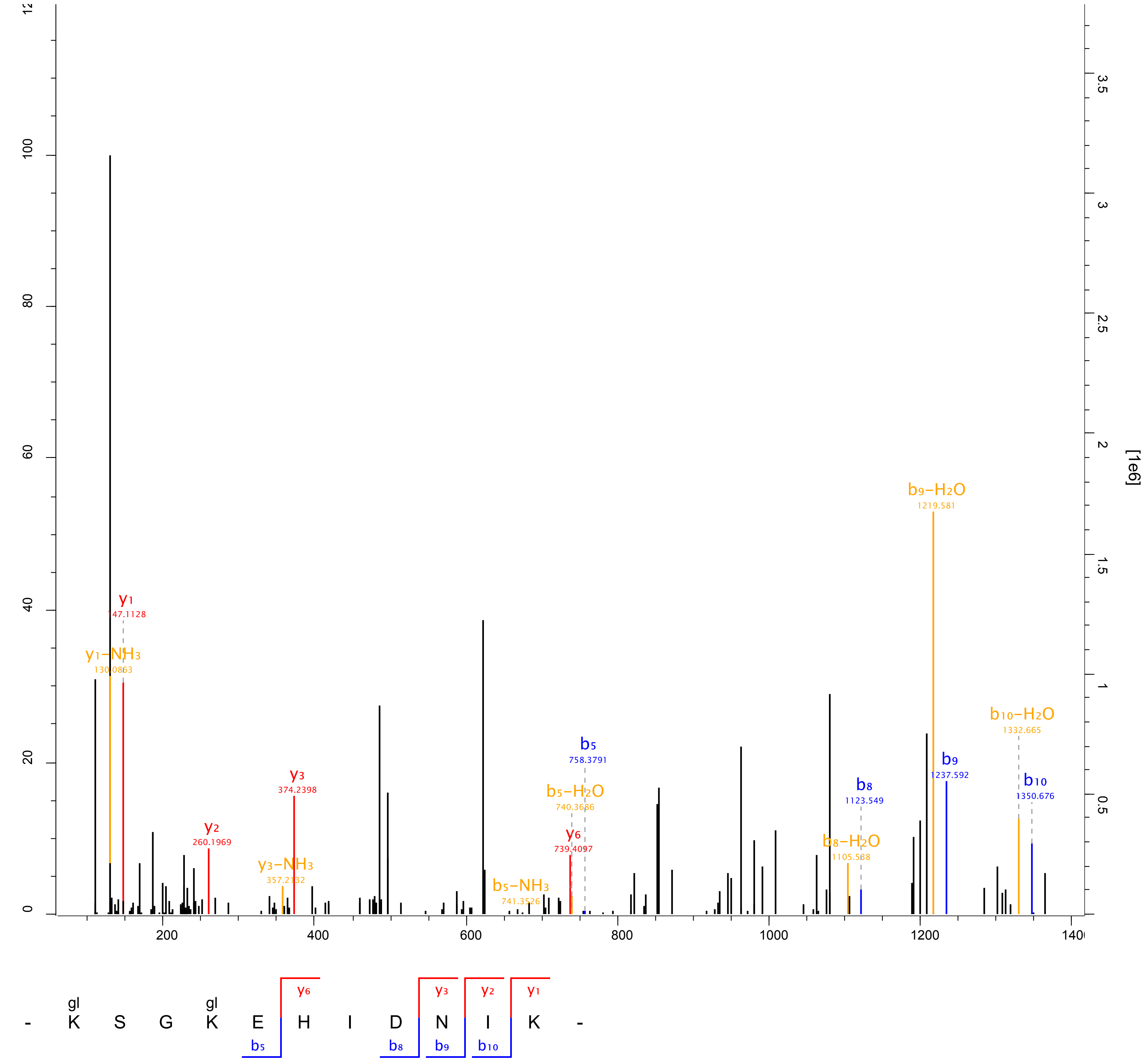

Raw file	Spectrum	Evidence ID	Scan Method	Score	m/z	Gene names
QE_SC_double_US_U2OS12221099_DMSO	932[illegible]	186212	FTMS; HCD	51.0[illegible]	470.2	H2BFS;HIST1H2BK;HIST1H2BI;HIST1H2BN;HIST1H2BL;HIST1H2BH;HIST2H2BF;HIST1H2BC;HIST1H2BD;HIST1H2

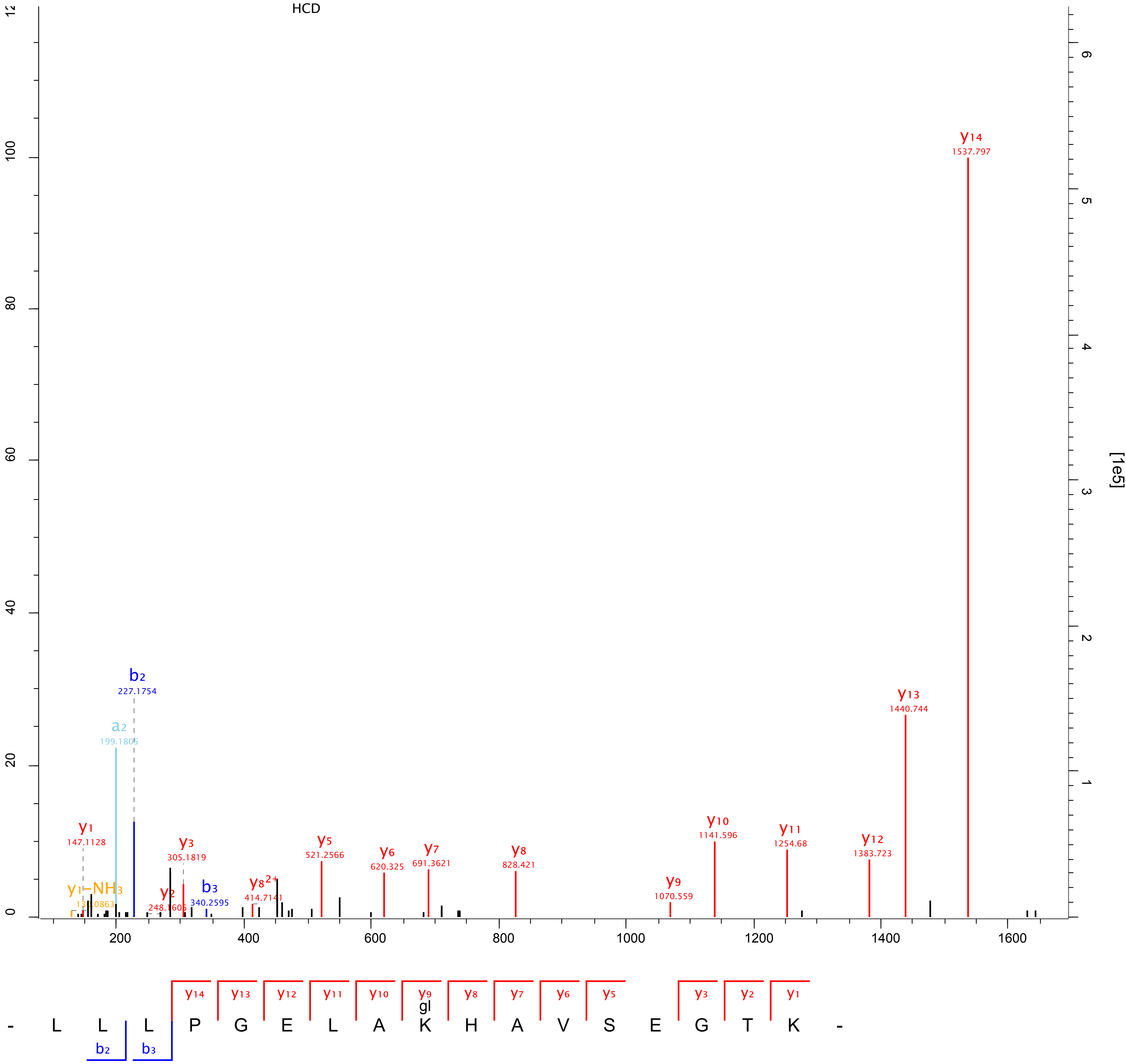

Raw file	Spectrum Evidence ID 186408	Scan	Method	Score	m/z	Gene names
QE_SC_double_US_U2OS12221099_MG132_BR1_TR3		16063	FTMS; HCD	48.71	675.72	DDIT4

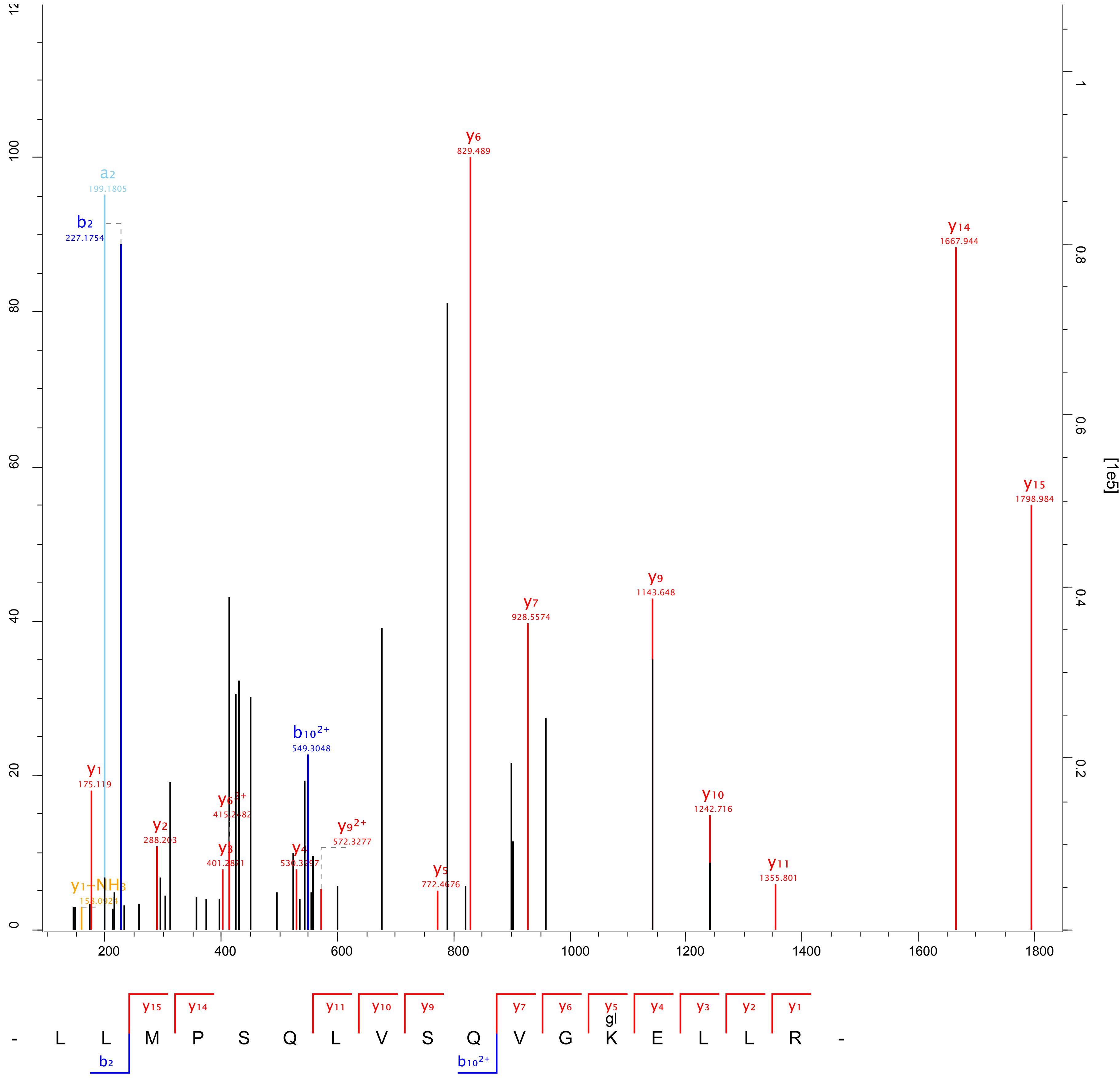

Raw file Spectrum Evidence ID 190691	Scan	Method	Score	m/z	Gene names
QE_SC_double_US_U2OS12221099_MG132_BR1_TR3	11785	FTMS; HCD	56.29	651.01	TRIM43B;TRIM43

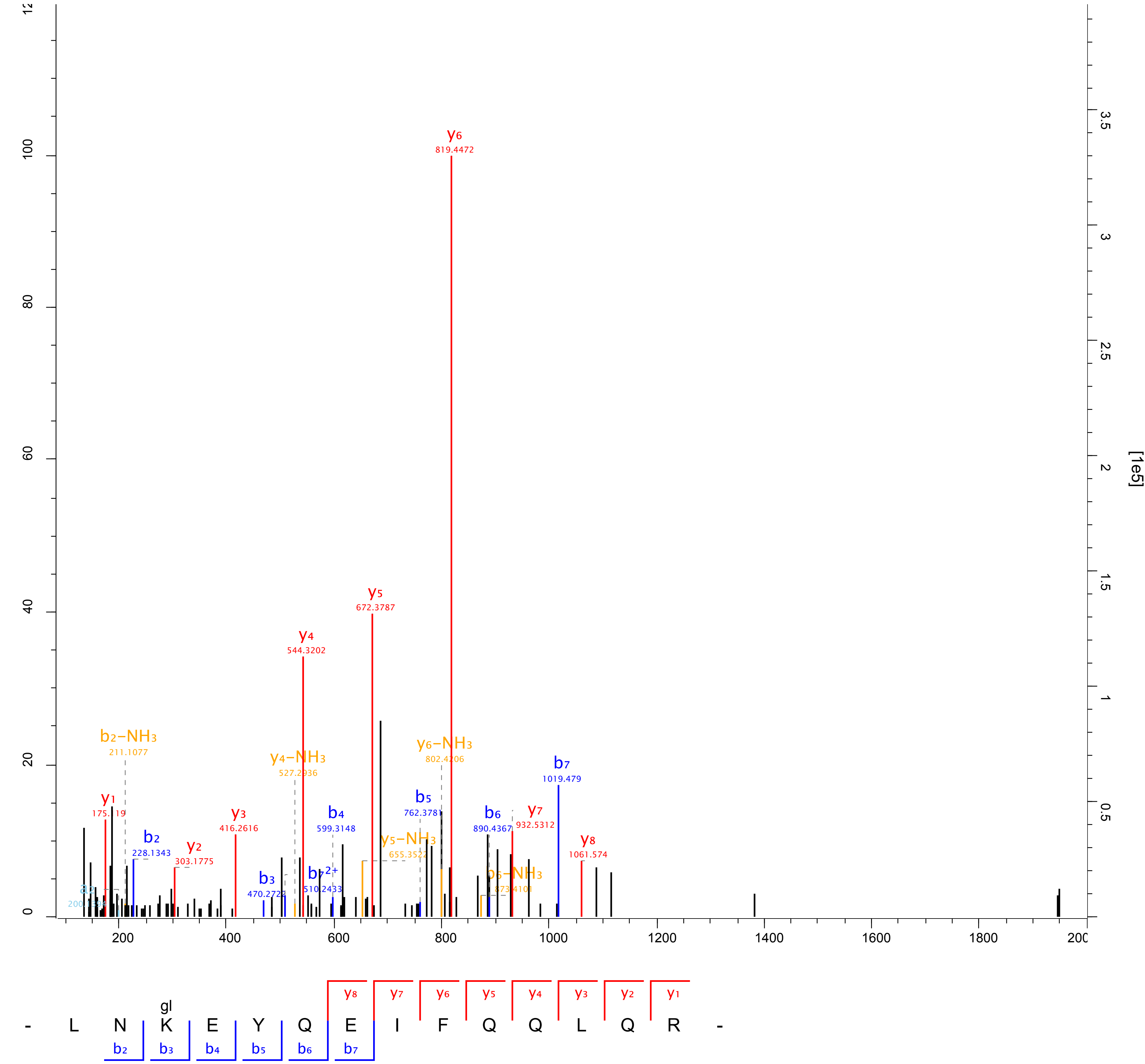

Raw file	Spectrum Evidence ID 214112	Scan	Method	Score	m/z	Gene names
QE_SC_double_US_U2OS12221099_MG132_BR4_TR3		9926	FTMS; HCD	65.18	460.6	UBB;UBC;RPS27A;UBA52

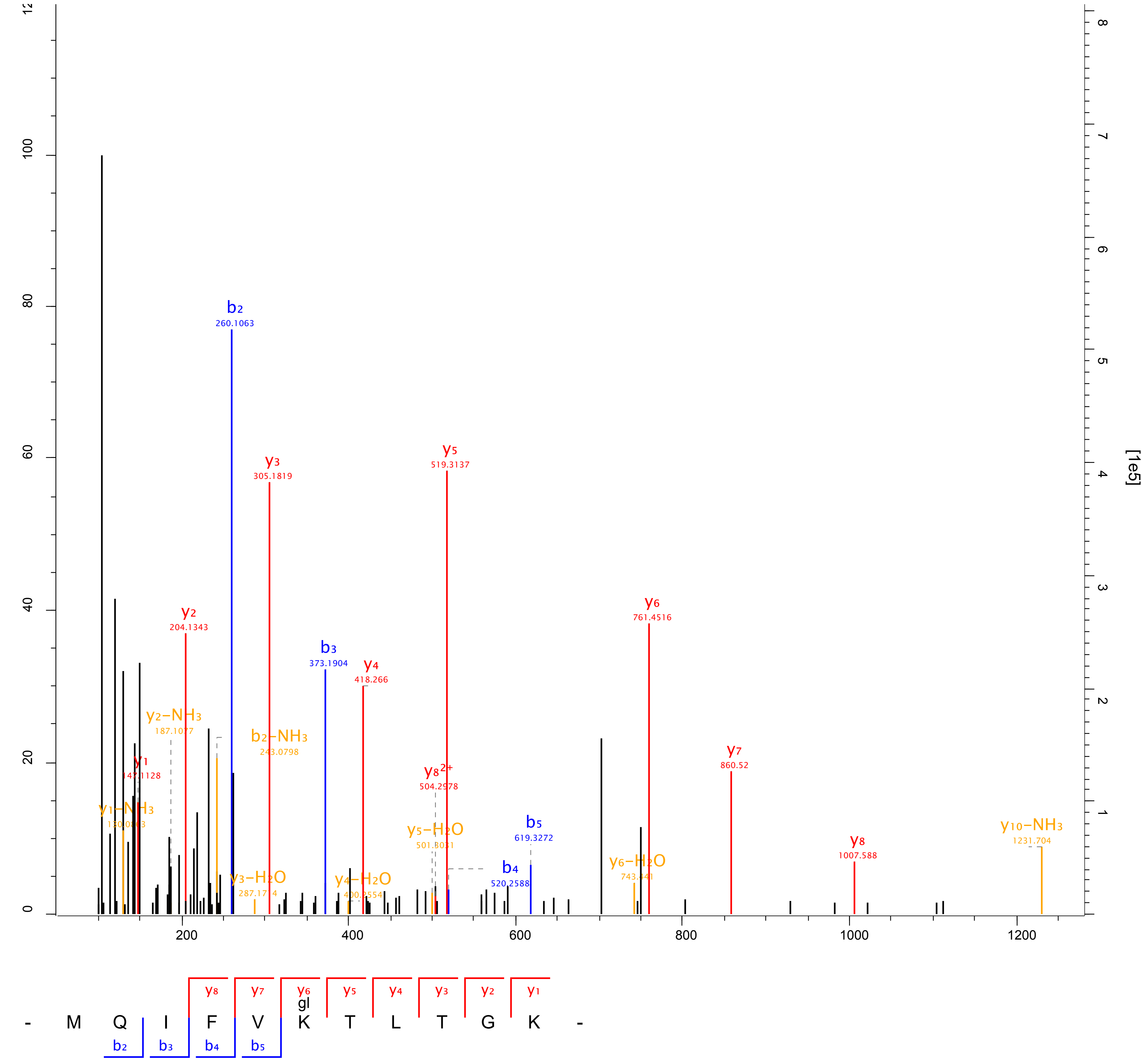

Raw file Spectrum Evidence ID 261072	Scan	Method	Score	m/z	Gene names
QE_SC_double_SU_U2OS12221099_MG132_BR2_TR1	5076	FTMS; HCD	43.59	634.61	SUMO1

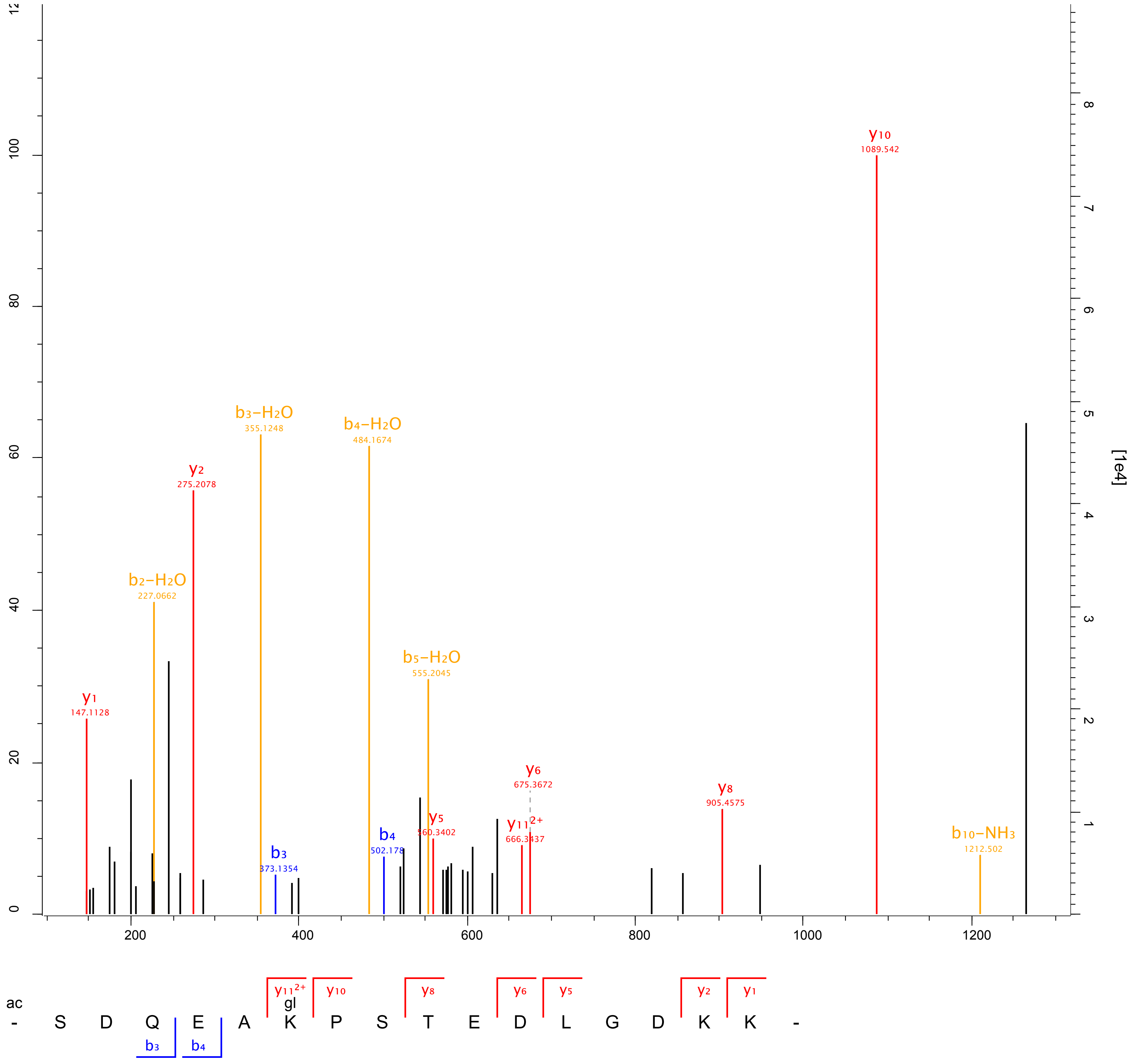

Raw file Spectrum Evidence ID 262213	Scan	Method	Score	m/z	Gene names
QE_SC_double_US_U2OS12221099_MG132_BR1_TR1	2472	FTMS; HCD	63.07	429.56	SUMO3

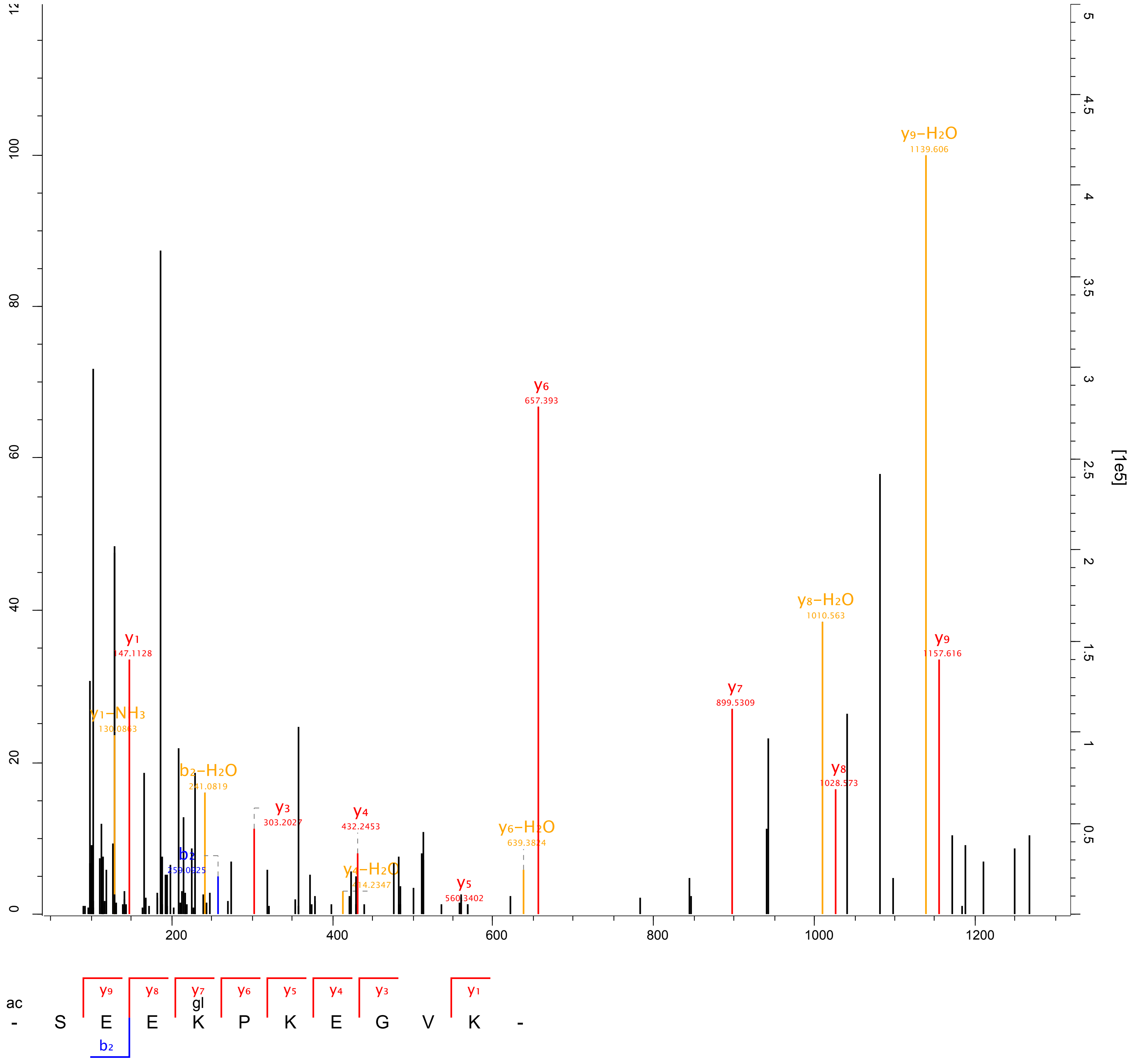

Raw file Spectrum Evidence ID 305379	Scan	Method	Score	m/z	Gene names
QE_SC_double_US_U2OS12221099_MG132_BR4_TR3	9891	FTMS; HCD	163.41	701.37	UBB;UBC;RPS27A;UBA52

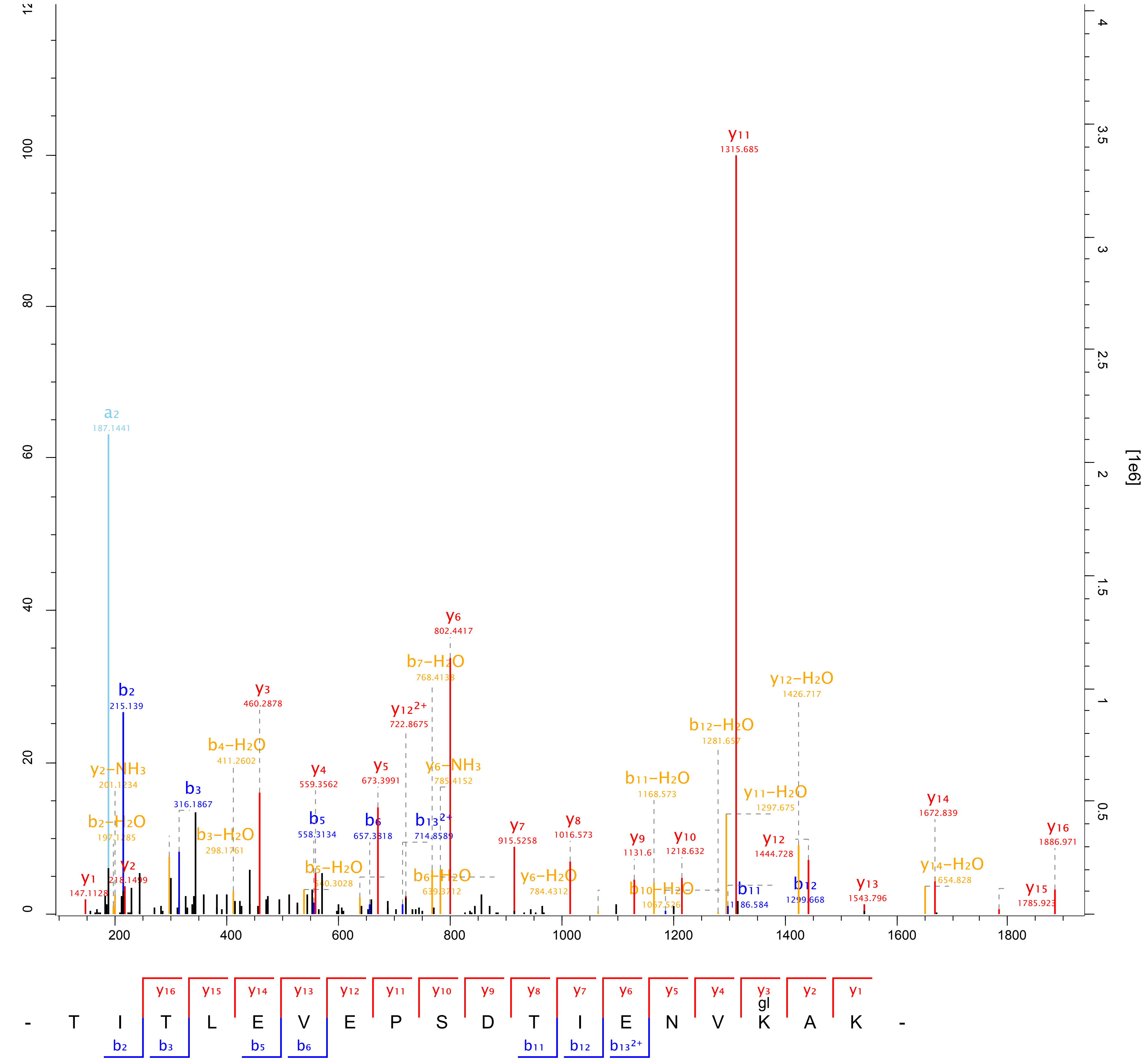

Raw file Spectrum Evidence ID 309193	Scan	Method	Score	m/z	Gene names
QE_SC_double_US_U2OS12221099_MG132_BR4_TR3	10169	FTMS; HCD	57.37	629.35	UBB;UBC;RPS27A;UBA52

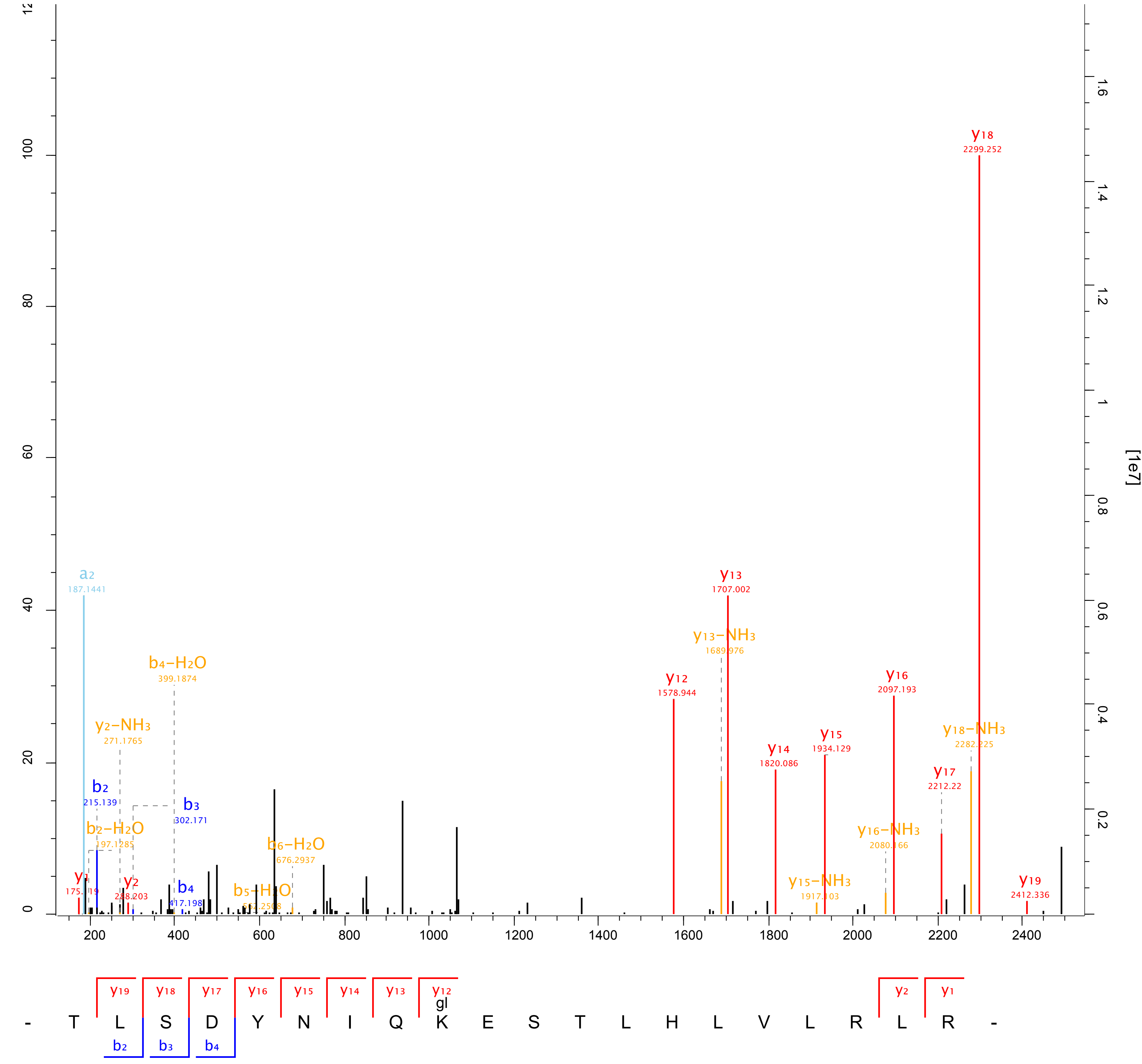

Raw file Spectrum Evidence ID 309895	Scan	Method	Score	m/z	Gene names
QE_SC_double_US_U2OS12221099_MG132_BR4_TR3	9701	FTMS; HCD	198.1	651.36	UBB;UBC;RPS27A;UBA52

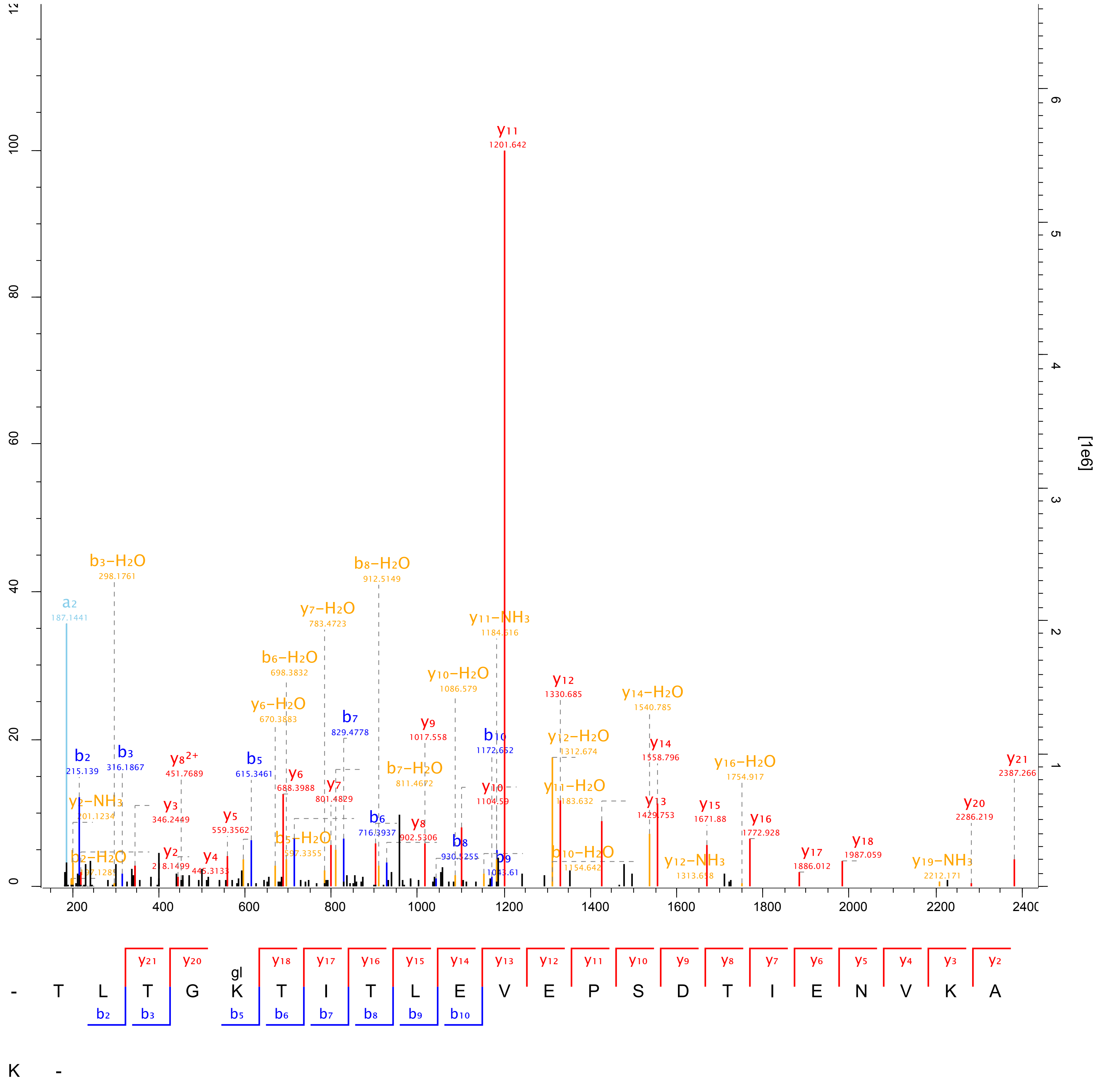

Raw file	Spectrum Evidence ID 322781	Scan	Method	Score	m/z	Gene names
QE_SC_double_US_U2OS12221099_MG132_BR4_TR3		8235	FTMS; HCD	58.59	530.63	SUMO3;SUMO4;SUMO2

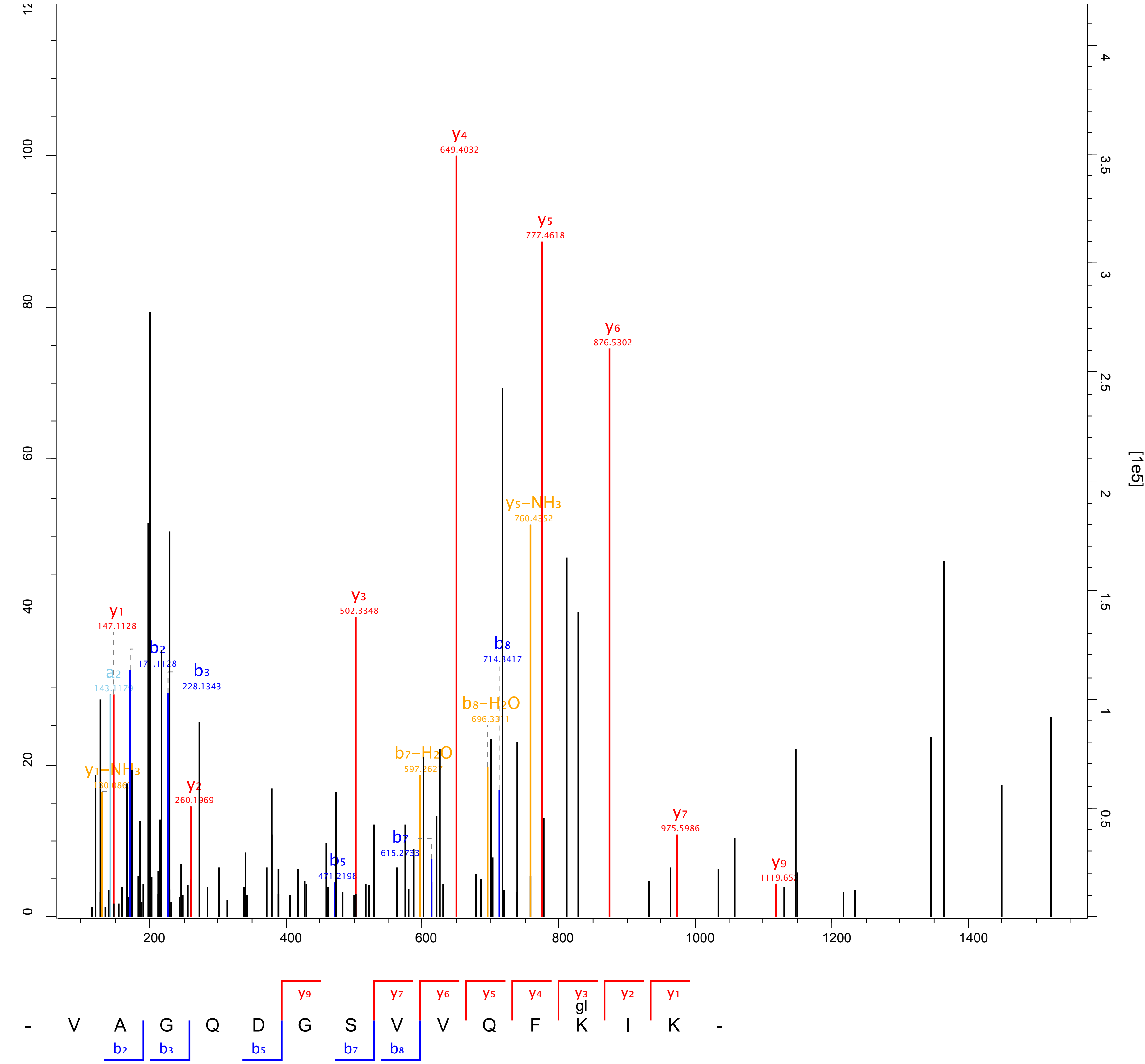

Raw file	Spectrum Evidence ID 343805	Scan	Method	Score	m/z	Gene names
QE_SC_double_US_U2OS12221099_MG132_BR1_TR3		13884	FTMS; HCD	75.56	725.11	HIST1H2AJ;HIST1H2AH;H2AFJ;HIST1H2AD;HIST1H2AG;HIST2H2AC;HIST2H2AA3;HIST1

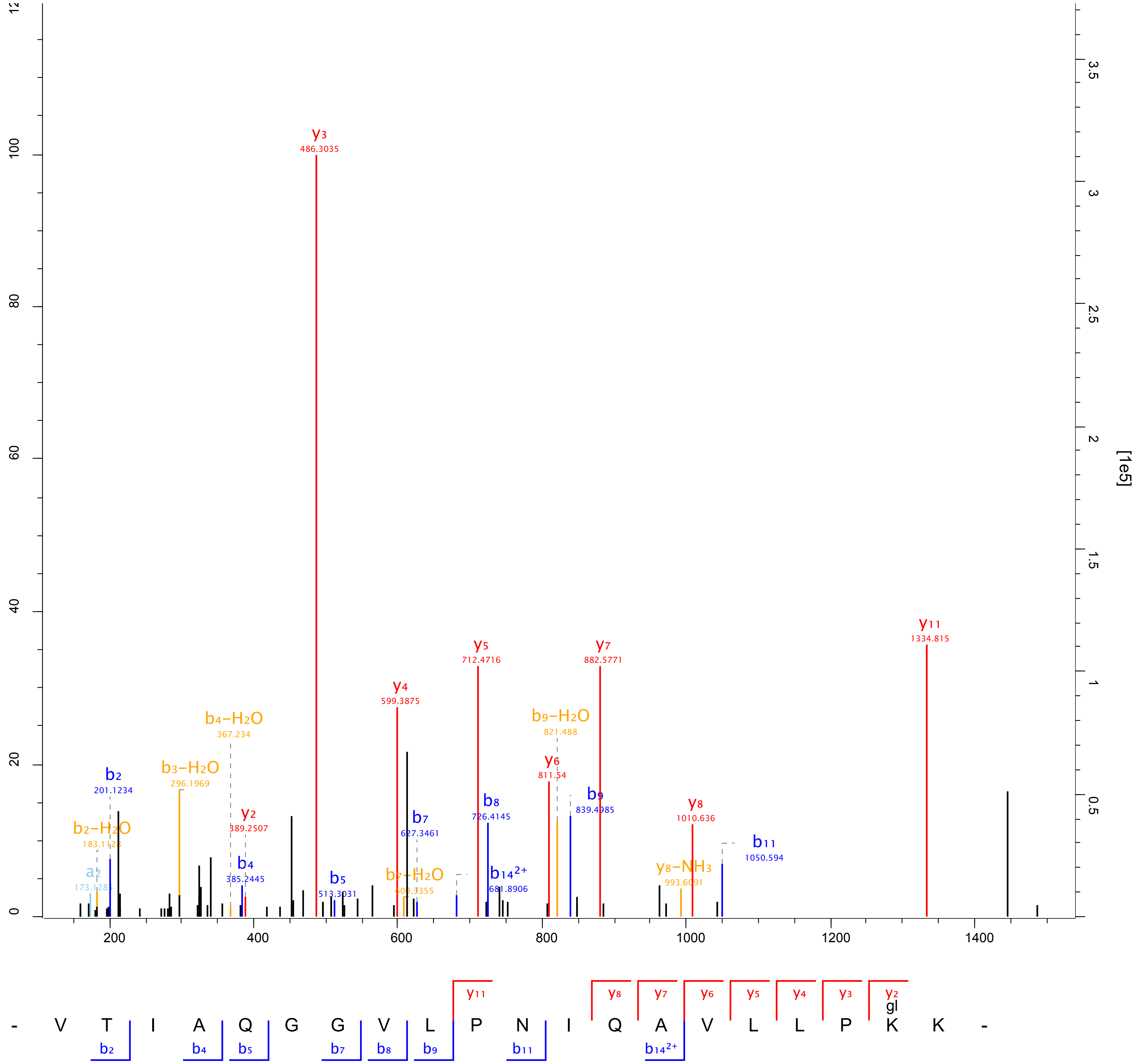

Raw file Spectrum Evidence ID 363652	Scan	Method	Score	m/z	Gene names
QE_SC_double_US_U2OS12221099_DMSO_BR3_TR1	4508	FTMS; HCD	155.08	529.27	RPS27A

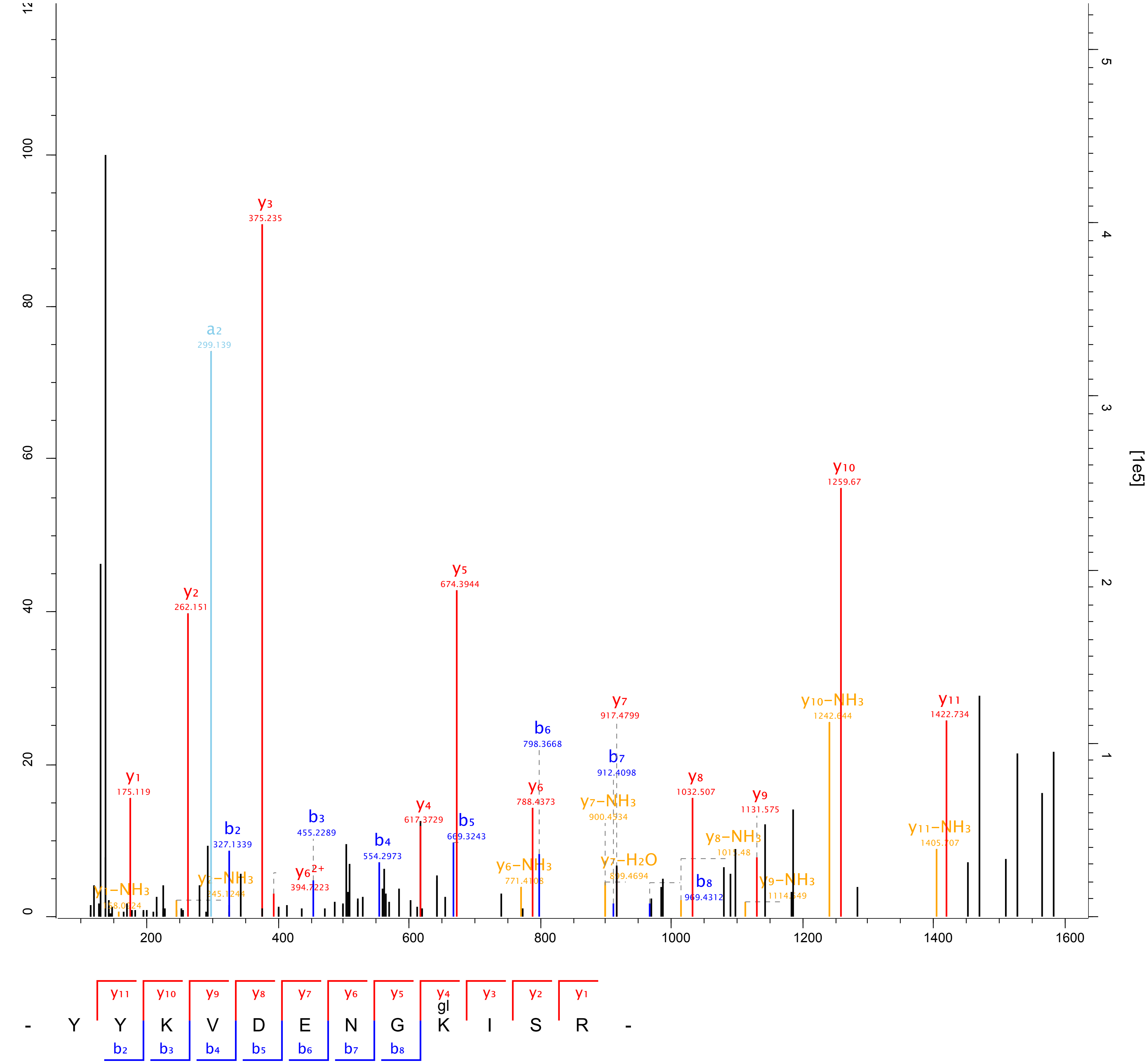

Supplement: Supplemental Data [file supp_TIR117.000152_4742_1_supp_6088_cw7v3b.pdf]
