# Supplementary material for: Converging Small Ubiquitin-like Modifier (SUMO) and Ubiquitin Signaling: Improved Methodology Identifies Co-modified Target Proteins
Source: Mol Cell Proteomics. 2017 Sep 26;16(12):2281–95. doi: 10.1074/mcp.TIR117.000152 (PMC5724187; doi:10.1074/mcp.TIR117.000152)
Supplement: Supplemental Data [file supp_TIR117.000152_4742_1_supp_6084_cw7v3c.pdf]

TITLE

Converging SUMO and ubiquitin signaling: improved methodology identifies co-modified target proteins

AUTHORS

Sabine A.G. Cuijpers[1], Edwin Willemstein[1] and Alfred C.O. Vertegaal[1]

AFFILIATION

[1]Department of Molecular Cell Biology, Leiden University Medical Center, 2300 RC Leiden, the Netherlands

E-mail: vertegaal@lumc.nl

RUNNING TITLE

Converging SUMO and ubiquitin signaling

SUPPLEMENTAL EXPERIMENTAL PROCEDURES

Cell culture and treatments

Human bone osteosarcoma epithelial (U2OS) cells and human embryonic kidney 293 (HEK293T) cells were cultured at 5% CO_2 and 37°C in Dulbecco's modified eagle's medium (DMEM, Gibco) including 10% fetal calf serum (FCS, Gibco), 100 U/ml penicillin and 100 mg/ml streptomycin (P/S, Gibco). The same condition was used to culture U2OS cell lines stably expressing a construct containing His_{10}-tagged SUMO2 or FLAG-tagged SUMO2 followed by an IRES and GFP sequence, which were selected by low GFP expression as described before(19, 20). The mature protein that is referred to as SUMO2 has the following amino acid sequence: MSEEKPKEGVKTENDHINLKVAGQDGSVVQFKIKRHTPLSKLMKAYCERQGLSMRQIR FRFDGQPINETDTPAQLEMEDEDTIDVFQQQTGG.

To create two complementary cell lines exogenously expressing both SUMO and ubiquitin, the U2OS His_{10}-SUMO2 cell line was infected with lentivirus encoding a FLAG-ubiquitin construct and the U2OS FLAG-SUMO2 cell line was infected with lentivirus encoding a His_{10}-ubiquitin construct at an MOI of 3 using 8 µg/ml polybrene. Both novel cell lines were selected using 2.5 µM puromycin (Calbiochem) to obtain stable co-expressing cell lines. As an additional control cell line, U2OS cells were infected with lentivirus encoding a His_{10}-ubiquitin construct and selected with puromycin as described above.

Cells were treated with 10 µM MG132 (Sigma) for 6 hours to inhibit the proteasome. As a control, cells were incubated with 0.1% dimethyl sulphoxide (DMSO, Sigma) for 6 hours.

To obtain RNF4 knockdown, cells were infected with lentivirus encoding either a control shRNA (SHC002) or three independent shRNAs (TRCN0000017054, TRCN0000272668 and TRCN0000284821) directed against RNF4 (Mission shRNA library, Sigma) at an MOI of 3 using polybrene. Three days after infection, cells were lysed.

Cells were synchronized in specific stages of the cell cycle using two independent blocking agents. Thymidine (Sigma) was added at a concentration of 4 mM to block cells at the start of S phase. After 16 hours, cells were washed twice with PBS and released from the block for the indicated amount of time in DMEM with FCS and P/S. Nocodazole (Sigma) was used at a final concentration of 0.1 µg/ml to block cells in prometaphase. After 20 hours cells were either lysed immediately or carefully washed twice with PBS to release them from their cell cycle arrest for the indicated amount of time in DMEM with FCS and P/S. Cells were collected using trypsin treatment and washed with PBS, before each sample was split into three parts and fixed for fluorescence-activated cell sorting (FACS) analysis, lysed to perform a His_{10}-pulldown (PD) or lysed to obtain a total lysate input control.

Lentivirus production

Lentiviruses were produced in HEK293T cells that were plated in 175 cm^2 flasks containing DMEM with FCS and without P/S. Cells were transfected with 7.5 µg pCMV-VSVG, 11.4 µg pMDLg-RRE, 5.4 µg pRSV-REV and 13.7 µg of the indicated vector plasmid using polyethyleneimine (PEI). The medium containing lentivirus was collected 48 and 72 hours after transfection and filtered using a 0.45 µm syringe filter (Pall Life

Sciences). Virus titers were determined using p24 elisa, while the virus was stored at -20°C until use.

Purification of co-modified proteins by His_{10}-pulldown and FLAG-immunoprecipitation

For each sample 200 million cells were lysed in 20 ml His_{10}-pulldown lysis buffer (6 M guanidine-HCl, 100 mM Na_2HPO_4/NaH_2PO_4 (pH 8.0) and 10 mM Tris-HCl (pH 8.0)), stored at -80°C and thawed at RT to continue sample preparation. After sonicating twice for 10 seconds at 30 Watts, samples were equalized using BCA Protein Assay Reagent (Thermo Scientific). Imidazole (pH 8.0, Merck Millipore) and β-mercaptoethanol (Sigma) were added to a final concentration of 50 mM and 5.0 mM, respectively. Samples were mixed for 15 minutes at room temperature, before adding 20 µl of Ni-NTA beads per 1 ml of lysis buffer and incubation overnight at 4°C under continuous rotation.

Subsequently, the beads were washed once with buffer 1, once with buffer 2, once with buffer 3 and twice with buffer 4 including three tube changes. Wash buffer 1 consisted of His_{10}-pulldown lysis buffer supplemented with 10 mM imidazole (pH 8.0), 5 mM β-mercaptoethanol and 0.1% Triton X-100. Wash buffer 2 contained 8 M urea, 100 mM Na_2HPO_4/NaH_2PO_4 (pH 8.0), 10 mM Tris-HCl (pH 8.0), 10 mM imidazole (pH 8.0), 5 mM β-mercaptoethanol and 0.1% Triton X-100. Wash buffer 3 consisted of 8 M urea, 100 mM Na_2HPO_4/NaH_2PO_4 (pH 6.3), 10 mM Tris-HCl (pH 6.3), 10 mM imidazole (pH 7.0) and 5 mM β-mercaptoethanol. Wash buffer 4 contained 8 M urea, 100 mM Na_2HPO_4/NaH_2PO_4 (pH 6.3), 10 mM Tris-HCl (pH 6.3) and 5 mM β-mercaptoethanol. Samples were eluted three times at RT with 400 µl elution buffer (7 M urea, 100 mM Na_2HPO_4/NaH_2PO_4, 10 mM Tris-HCl (pH 7.0), 500 mM imidazole (pH 7.0) and 70 mM chloroacetamide (CAA)).

The combined elution was passed through pre-washed 0.45 µm filter columns (Millipore) to remove any residual beads and subsequently concentrated on pre-washed 100 kDa cut off filters (SartoriusStedim). Sample volumes were equalized to 25.0 µl, before taking 10% of the samples as pulldown controls for immunoblot analysis. The remaining part of the sample was slowly and step-wise diluted on ice in 50 mM Na_2HPO_4/NaH_2PO_4 (pH 7.0), 10 mM Tris-HCl (pH 7.0), 100 mM NaCl and 70 mM CAA to a final volume of 1 ml. After centrifugation for 45 minutes at 13200 rpm and 4°C, the supernatant was transferred to a new tube and 100 µl of anti-FLAG-M2 beads (Sigma) was added per sample. NuPAGE LDS Sample Buffer (LDS, Life technologies) was added to solubilize any potential proteins in the pellet as a control for the renaturation step. After incubation of the supernatant with the beads for 90 minutes at 4°C, samples were washed twice with buffer 5, twice with buffer 6 and three times with buffer 7 including three tube changes. Buffer 5 consisted of 50 mM Tris-HCl (pH 7.5), 150 mM NaCl and 70 mM CAA. Buffer 6 contained 50 mM Tris-HCl (pH 7.5) and 150 mM NaCl, while buffer 7 consisted of 50 mM ammonium bicarbonate (ABC, Sigma). As a control for immunoblot analysis, 10% of the beads was eluted in LDS. The remaining beads were resuspended in 100 µl buffer 7 supplemented with 0.25 µg trypsin (Promega) per sample and incubated overnight at 37°C.

Samples were passed through pre-washed 0.45 µm filter columns to remove the beads, before trifluoroacetic acid (TFA, Sigma) was added to a final concentration of 2% to acidify the samples. Stage tips containing C18 (Sigma) were activated by passing HPLC-grade methanol (Sigma), washed with 80% acetonitrile (ACN, Sigma) in 0.1% formic acid (FA, Sigma) and equilibrated with 0.1% FA. Subsequently, the samples were loaded on these stage tips. Upon washing twice with 0.1% FA, the stage tips were dried

completely and eluted twice with 80% ACN. The samples were vacuum dried using a SpeedVac RC10.10 (Jouan), redissolved in 0.1% FA and transferred to autoloader vials before measurement by mass spectrometry.

Electrophoresis, immunoblotting and antibodies

Samples to determine total protein levels were lysed in total lysate input buffer (1% SDS, 1% NP-40, 50 mM Tris (pH 7.5) and 150 mM NaCl) and equalized using BCA Protein Assay Reagent before immunoblot analysis. Upon addition of dithiothreitol (DTT, Sigma) and LDS, each sample for immunoblot was incubated at 70°C for 10 minutes. Proteins were separated on Novex 4-12% Bis-Tris Plus gradient gels (Life Technologies) in MOPS buffer for 45 minutes at 165 Volt. Subsequently, proteins were transferred onto Hybond nitrocellulose membranes (GE Healthcare) in cold transfer buffer at 25 V for 3 hours. Membranes were stained with Ponceau S (Sigma) to confirm equal loading and blocked in PBS containing 0.05% Tween-20 (Merck) and 8% milk powder (blocking solution) for 1 hour at RT. Membranes were incubated with primary antibodies diluted in blocking solution at 4°C overnight, including mouse monoclonal anti-polyHistidine Clone HIS-1 (Sigma), mouse monoclonal anti-SUMO2/3 (Abcam), mouse monoclonal anti-FLAG M2 (Sigma), mouse monoclonal anti-ubiquitin (Santa-Cruz Biotechnology), rabbit monoclonal anti-KIF23 (Epitomics), rabbit polyclonal anti-MIS18BP1 (Bethyl) and rabbit polyclonal anti-RNF4(22). Membranes were washed three times at RT for 10 minutes in PBS with 0.05% Tween-20 (PBS/T), followed by 1 hour incubation at 4°C with secondary antibodies donkey anti-rabbit or goat anti-mouse (Pierce) diluted in blocking solution. After washing the membranes three times for 10 minutes in PBS/T at 4°C,

Pierce ECL 2 immunoblotting substrate (Life Technologies) was used to visualize the signal on RX Medical films (Fuji).

Fluorescence-activated cell sorting analysis

Cells were harvested as described before and resuspended in PBS. Subsequently, cells were fixed by addition of ice-cold ethanol to a final concentration of 70% and incubation at 4°C for at least overnight. Prior to flow cytometry analysis, cells were centrifuged at 1200 rpm for 2 minutes and washed in PBS containing 2% FCS. After another round of centrifugation, cells were resuspended in PBS with 2% FCS, 25 µg/ml propidium iodide (Sigma) and 100 µg/ml RNAse A (Sigma). After staining for 30 minutes at 37°C, cellular DNA content was measured by flow cytometry with the BD LSRII system and BD FACS DIVA software (BD Bioscience Clontech).

SUPPLEMENTAL FIGURE LEGENDS

Supplemental Fig. S1. **Limited abundance of mixed SUMO-ubiquitin chains.** *A.* Cartoon depicting potential co-modified entities purified from cells expressing His_{10}-SUMO2 and the effect of $SENP2_{CD}$ or $USP2_{CD}$ treatment on each option. *B.* His_{10}-SUMO2 pulldown samples were treated with or without $SENP2_{CD}$ and/or $USP2_{CD}$. Immunoblotting was carried out using antibodies against SUMO2/3 and ubiquitin. *C.* U2OS cells expressing His_{10}-SUMO2 were treated with MG132 for 6 hours to inhibit the proteasome before samples were lysed. His_{10}-pulldown was performed and the elutions were treated with or without $SENP2_{CD}$ and/or $USP2_{CD}$. Samples were analyzed by immunoblotting with antibodies against SUMO2/3 and ubiquitin.

Supplemental Fig. S2. **Verification of improved co-purification method by immunoblotting.** *A.* U2OS cells with or without expression of His_{10}-SUMO2 and FLAG-ubiquitin were treated with DMSO or MG132 to inhibit the proteasome. Samples were taken before the His_{10}-pulldown (input), after the His_{10}-pulldown (PD) and after the FLAG-IP (PD+IP) and analyzed by immunoblotting with an antibody against polyHistidine. An equal percentage of the sample was loaded for the PD and PD+IP samples to enable comparison. *B.* The same samples as described under A were immunoblotted using an antibody against FLAG. *C.* To analyze SUMOylated protein renaturation during the dilution between purifications, equal amounts of starting material were loaded of samples after the His_{10}-pulldown (PD), after the dilution and centrifugation (pellet), and after the FLAG-IP (PD+IP). Analysis was performed by immunoblotting using an antibody against polyHistidine. *D.* U2OS cells with or without

expression of His_{10}-ubiquitin and FLAG-SUMO2 were treated with DMSO or MG132. Samples were taken before the His_{10}-pulldown (input), after the His_{10}-pulldown (PD) and after the FLAG-IP (PD+IP) and analyzed by immunoblotting using an antibody against polyHistidine. An equal percentage of the sample was loaded for the PD and PD+IP samples to enable comparison. *E.* The same samples as described under D were immunoblotted using an antibody against FLAG. *F.* To analyze ubiquitylated protein renaturation during the dilution between purifications, equal amounts of starting material were loaded of samples after the His_{10}-pulldown (PD), after the dilution and centrifugation (pellet), and after the FLAG-IP (PD+IP). Analysis was performed by immunoblotting using an antibody against polyHistidine.

Supplemental Fig. S3. **Additional analysis reveals specific enrichment for DNA modification processes amongst co-modified targets under control conditions.** *A.* Samples from similar conditions of both approaches were pooled together, resulting in two groups treated with DMSO and two groups treated with MG132. Volcano plots show p value (as –Log10(p)) and difference (as Log2FC) for each protein, including significantly enriched co-modified targets in color with a q value below 0.03. *B.* Gene ontology analysis was performed for the co-modified proteins identified after DMSO as well as MG132 treatment. Eight biological processes were identified to be significantly enriched specifically under control conditions and not after inhibition of the proteasome, of which their Benjamini Hochberg corrected p values (as -Log10(FDR) are shown. *C.* Similar to B, but showing the enrichment factor for the DMSO specific enriched biological processes.

Supplemental Fig. S4. **Validation of additional control cell line by immunoblotting.** *A.* Parental U2OS cells, U2OS cells expressing His_{10}-ubiquitin and U2OS cells expressing His_{10}-ubiquitin and FLAG-SUMO2 were lysed and samples were analyzed by immunoblotting using antibodies against ubiquitin, polyHistidine, SUMO2/3 and FLAG. *B.* Parental U2OS cells, U2OS cells expressing His_{10}-SUMO2 and U2OS cells expressing His_{10}-SUMO2 and FLAG-ubiquitin were lysed and expression levels were analyzed by immunoblotting using antibodies against SUMO2/3, polyHistidine, ubiquitin and FLAG.

Supplemental Fig. S5. **Additional verification of MIS18BP1 and KIF23 as co-modified targets upon inhibition of the proteasome.** *A.* Parental U2OS cells, U2OS cells expressing His_{10}-SUMO2 and U2OS cells expressing His_{10}-SUMO2 and FLAG-ubiquitin were treated with DMSO or MG132 to inhibit the proteasome. Samples were taken before the His_{10}-pulldown (input), after the His_{10}-pulldown (PD) and after the FLAG-IP (PD+IP). Samples were analyzed by immunoblotting with antibodies against MIS18BP1, KIF23, polyHistidine and FLAG. An equal percentage of the sample was loaded for the PD and PD+IP samples to enable comparison. Asterisk represents an a-specific band. *B.* A membrane with input samples from figure S5A was stained with Ponceau S to confirm equal loading. *C.* A membrane with input samples from figure 5 was stained with Ponceau S to confirm equal loading.

Supplemental Fig. S6. **Verification of cell cycle synchronization by flow cytometry.** *A.* U2OS cells expressing His_{10}-SUMO2 were blocked using thymidine or nocodazole and released for the indicated time to reach specific stages of the cell cycle. After

fixation in ethanol, cells were stained with propidium iodide and DNA content was measured by flow cytometry to verify cell cycle synchronization. Graphs show DNA content on the x-axis and cell count on the y-axis. *B.* U2OS cells expressing His_{10}-ubiquitin were blocked using thymidine or nocodazole and released for the indicated amount of time to reach specific stages of the cell cycle. After fixation in ethanol, cells were stained with propidium iodide and DNA content was measured by flow cytometry to verify cell cycle synchronization. Graphs show DNA content on the x-axis and cell count on the y-axis.

SUPPLEMENTAL TABLE LEGENDS

Supplemental Table S1. **All identified peptides.** A list of all peptides identified by MaxQuant on the RAW Peptides sheet. The second sheet explains which sample names belongs to each exact sample measured.

Supplemental Table S2. **All identified protein groups.** A list of all protein groups for which peptides were identified by MaxQuant on the RAW ProteinGroups sheet. The second sheet explains which sample names belongs to each exact sample measured.

Supplemental Table S3. **All identified diGly (K) sites.** A list of all peptides identified by MaxQuant to be modified by a diGly motif on the RAW GlyGly (K) Sites sheet. The second sheet explains which sample names belongs to each exact sample measured.

Supplemental Table S4. **Co-modified targets under control conditions and upon inhibition of the proteasome identified by overlapping both approaches.** Proteins with a q value below 0.03 for both approaches upon MG132 treatment can be found on the co-modified targets upon MG132 sheet, while those identified under DMSO conditions are shown on the co-modified targets upon DMSO sheet. For both lists, additional columns are included summarizing both approaches into one q value (-log10(x*y)) and one difference value ((x+y)/2) used for shape size and color in the visualized networks. The third sheet explains which sample names belongs to each exact sample measured.

Supplemental Table S5. **Co-modified targets under control conditions and upon inhibition of the proteasome identified by pooling both approaches.** Proteins with a q value below 0.03 amongst the pooled samples upon MG132 treatment can be found on the Co-modified upon MG132 pooled sheet, while those identified under DMSO conditions are shown on the Co-modified targets upon DMSO pooled sheet. The third sheet explains which sample names belongs to each exact sample measured.

Supplemental Table S6. **Enrichment analysis of gene ontology based biological processes.** For each biological process the enrichment factor, FDR and -Log10(FDR) obtained from Fisher Exact testing amongst the co-modified targets upon MG132 are shown on the GOBP enrichment targets MG132 sheet, while those obtained from testing amongst the co-modified targets upon DMSO are shown on the GOBP enrichment targets DMSO sheet. On the third sheet all biological processes tested amongst the co-modified targets obtained from the pooled approach analysis are shown for the DMSO and MG132 conditions. Rows marked in orange represent the biological processes that were significantly enriched amongst the co-modified targets upon DMSO (with a FDR value below 0.03), while not showing a significant enrichment amongst the targets upon inhibition of the proteasome.

Supplemental Table S7. **Identification of the co-modified proteins belonging to each selected biological process.** For each co-modified target upon MG132 treatment, their gene ontology annotated biological processes were studied and proteins annotated to the selected processes were marker by a plus sign.

Supplemental Table S8. **DiGly sites enriched amongst co-modified target proteins.** A list of peptides modified by a diGly motif enriched in the samples purified for proteins modified by both SUMO and ubiquitin. For each peptide, the location of the diGly motif within the protein is shown as well as the best localization spectrum evidence ID, which corresponds to the spectra shown in PDF S1.

Supplemental Table S9. **Co-modified targets and their published sites.** A list of the proteins identified to be significantly co-modified by SUMO and ubiquitin, marked with a plus sign if one or more SUMOylation or ubiquitylation sites are published.

SUPPLEMENTAL PDF LEGENDS

Supplemental PDF S1. **Spectra of peptides with diGly (K) sites enriched amongst co-modified target proteins.** A list of the best localization spectrum for each peptide identified to be modified by a diGly motif and enriched in the samples from cell lines exogenously expressing both SUMO2 and ubiquitin compared to the parental control samples.

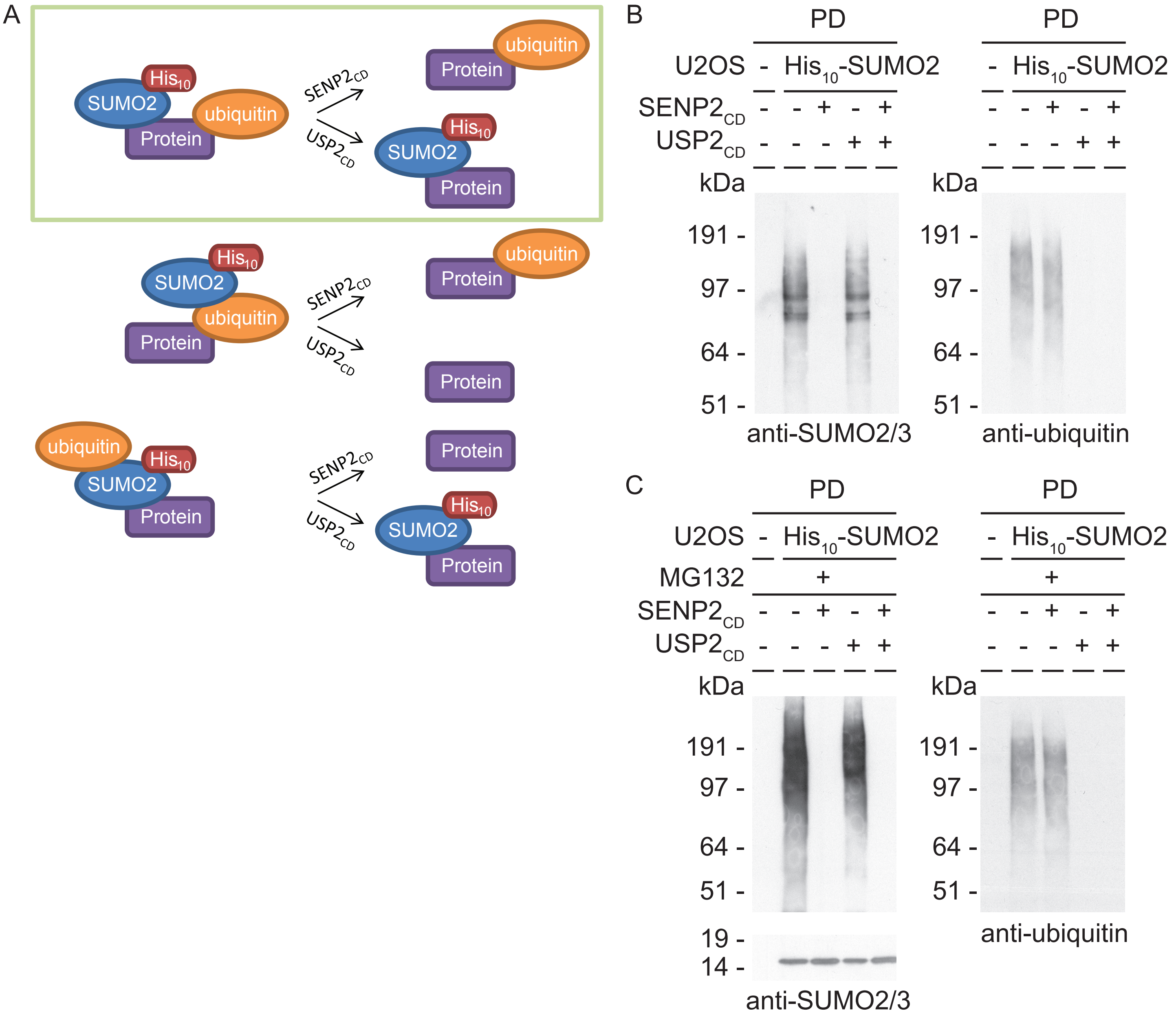
A
His10
SUMO2
ubiquitin
Protein
SENP2CD
USP2CD
B
PD
U2OS
His10-SUMO2
SENP2CD
USP2CD
kDa
191
97
64
51
anti-SUMO2/3
anti-ubiquitin
C
MG132
19
14

Figure S1

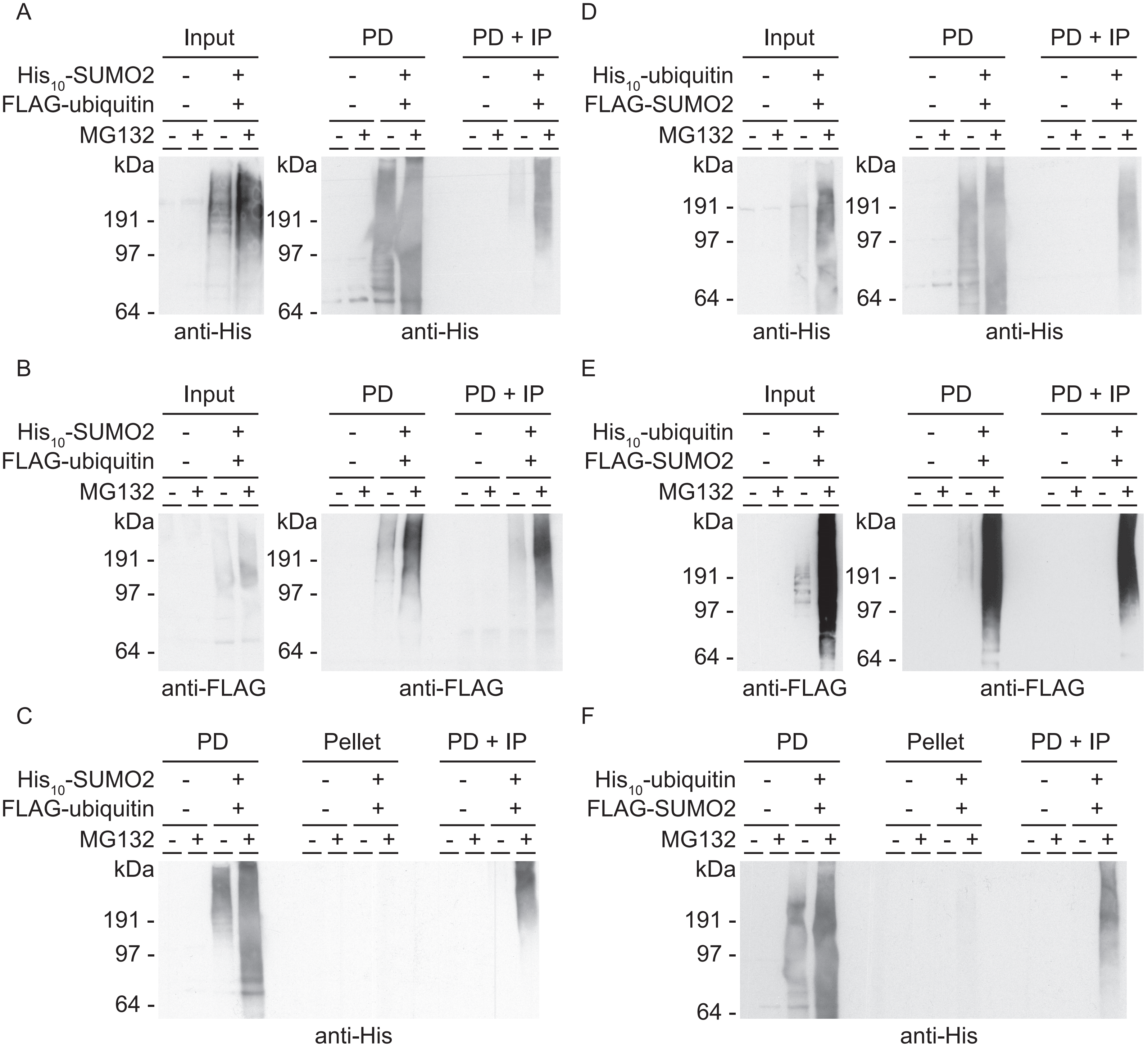
A
Input
PD
PD + IP
His10-SUMO2
FLAG-ubiquitin
MG132
kDa
191 -
97 -
64 -
anti-His
B
Input
PD
PD + IP
His10-SUMO2
FLAG-ubiquitin
MG132
kDa
191 -
97 -
64 -
anti-FLAG
C
PD
Pellet
PD + IP
His10-SUMO2
FLAG-ubiquitin
MG132
kDa
191 -
97 -
64 -
anti-His
D
Input
PD
PD + IP
His10-ubiquitin
FLAG-SUMO2
MG132
kDa
191 -
97 -
64 -
anti-His
E
Input
PD
PD + IP
His10-ubiquitin
FLAG-SUMO2
MG132
kDa
191 -
97 -
64 -
anti-FLAG
F
PD
Pellet
PD + IP
His10-ubiquitin
FLAG-SUMO2
MG132
kDa
191 -
97 -
64 -
anti-His

Figure S2

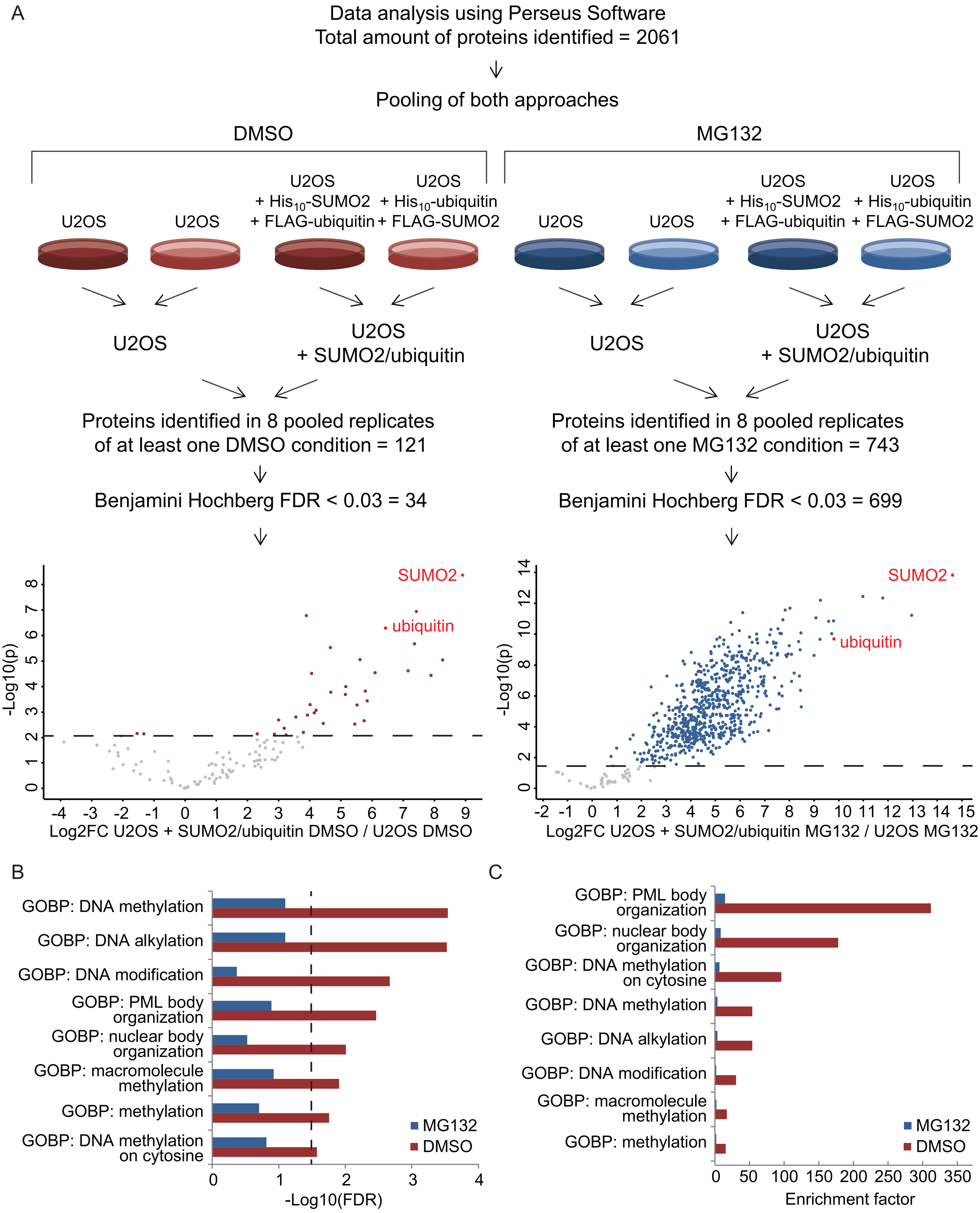

A
Data analysis using Perseus Software
Total amount of proteins identified = 2061
Pooling of both approaches
DMSO
MG132
U2OS
U2OS
U2OS + His10-SUMO2 + FLAG-ubiquitin
U2OS + His10-ubiquitin + FLAG-SUMO2
U2OS
U2OS + SUMO2/ubiquitin
Proteins identified in 8 pooled replicates of at least one DMSO condition = 121
Benjamini Hochberg FDR < 0.03 = 34
Proteins identified in 8 pooled replicates of at least one MG132 condition = 743
Benjamini Hochberg FDR < 0.03 = 699
SUMO2
ubiquitin
-Log10(p)
Log2FC U2OS + SUMO2/ubiquitin DMSO / U2OS DMSO
Log2FC U2OS + SUMO2/ubiquitin MG132 / U2OS MG132
B
GOBP: DNA methylation
GOBP: DNA alkylation
GOBP: DNA modification
GOBP: PML body organization
GOBP: nuclear body organization
GOBP: macromolecule methylation
GOBP: methylation
GOBP: DNA methylation on cytosine
MG132
DMSO
-Log10(FDR)
C
GOBP: PML body organization
GOBP: nuclear body organization
GOBP: DNA methylation on cytosine
GOBP: DNA methylation
GOBP: DNA alkylation
GOBP: DNA modification
GOBP: macromolecule methylation
GOBP: methylation
MG132
DMSO
Enrichment factor

Figure S3

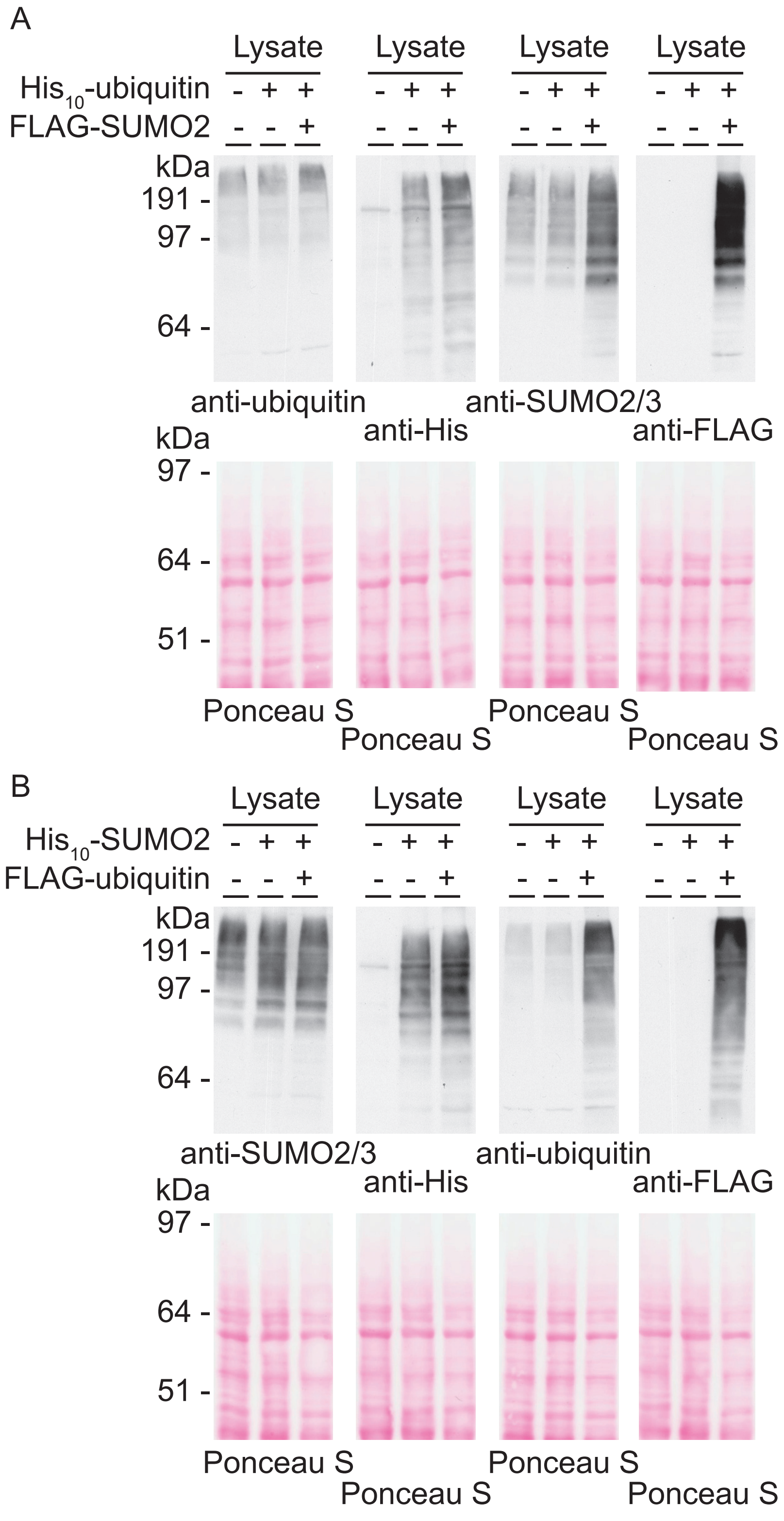
A
Lysate
Lysate
Lysate
Lysate
His10-ubiquitin - + +
FLAG-SUMO2 - - +
kDa
191 -
97 -
64 -
anti-ubiquitin
anti-His
anti-SUMO2/3
anti-FLAG
kDa
97 -
64 -
51 -
Ponceau S
Ponceau S
Ponceau S
Ponceau S
B
Lysate
Lysate
Lysate
Lysate
His10-SUMO2 - + +
FLAG-ubiquitin - - +
kDa
191 -
97 -
64 -
anti-SUMO2/3
anti-His
anti-ubiquitin
anti-FLAG
kDa
97 -
64 -
51 -
Ponceau S
Ponceau S
Ponceau S
Ponceau S

Figure S4

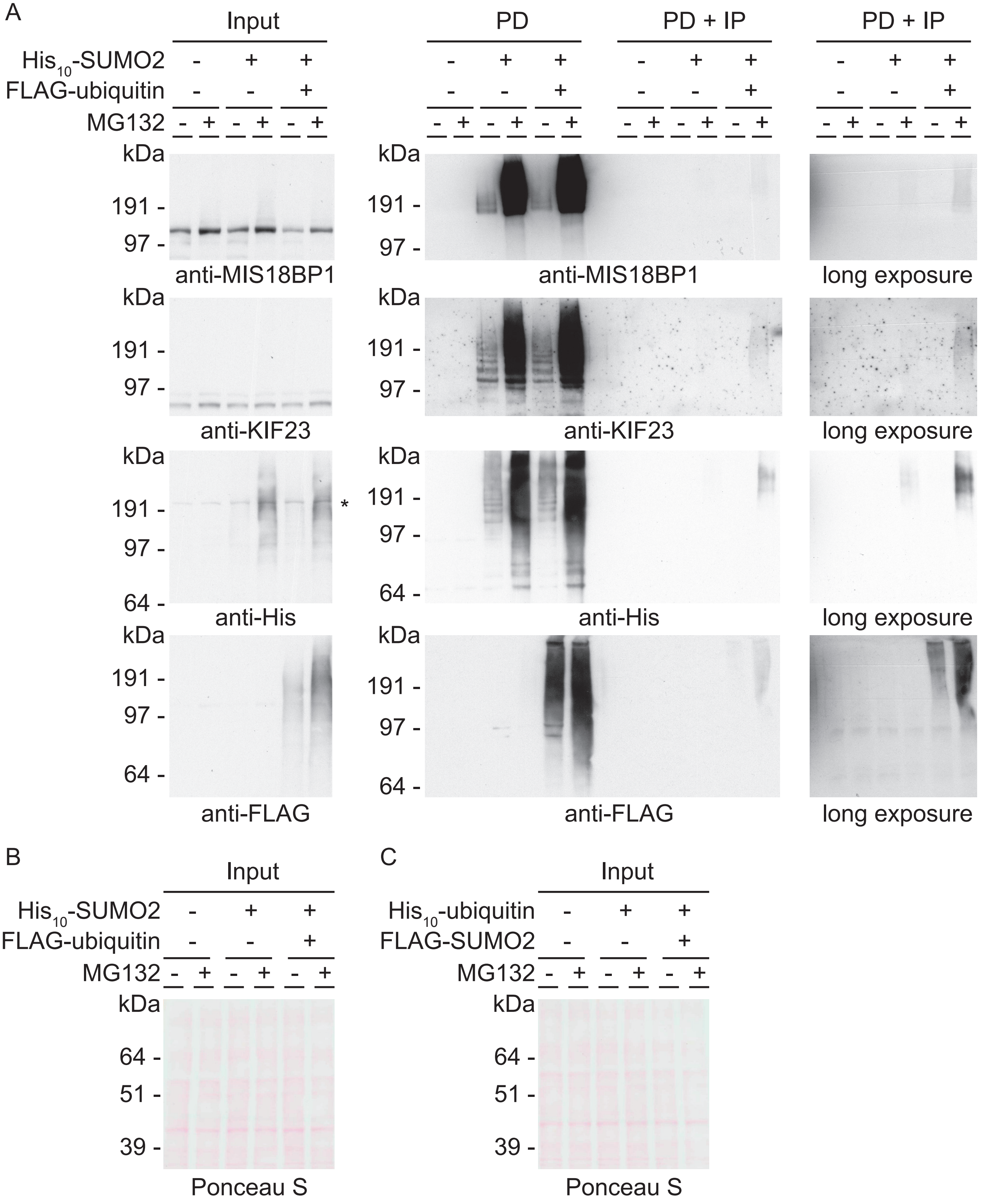
A
Input
PD
PD + IP
PD + IP
His10-SUMO2
FLAG-ubiquitin
MG132
kDa
191 -
97 -
64 -
anti-MIS18BP1
anti-KIF23
anti-His
anti-FLAG
long exposure
*
B
Input
His10-SUMO2
FLAG-ubiquitin
MG132
kDa
64 -
51 -
39 -
Ponceau S
C
Input
His10-ubiquitin
FLAG-SUMO2
MG132
kDa
64 -
51 -
39 -
Ponceau S

Figure S5

Figure S6

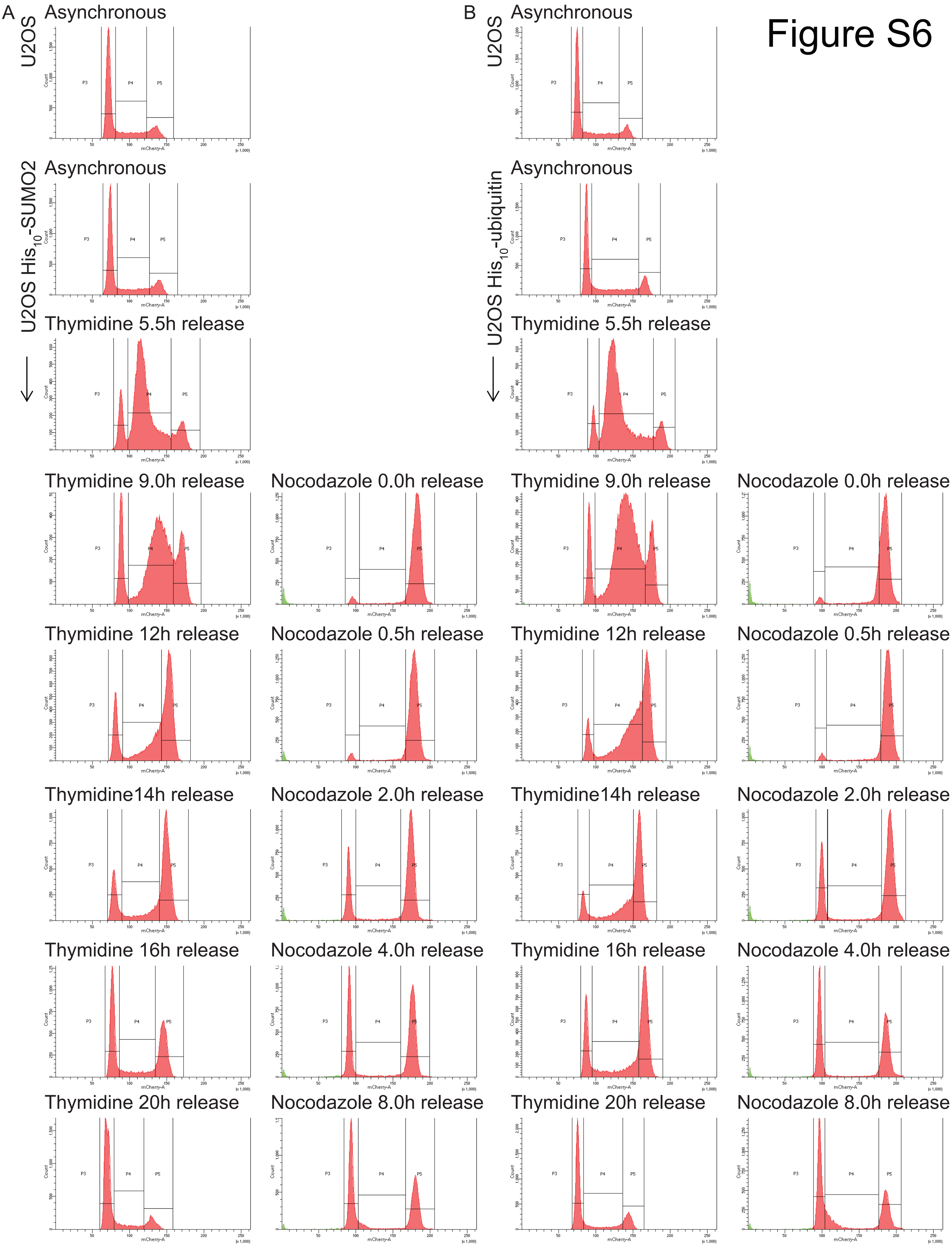
A
B
U2OS
Asynchronous
U2OS His$_{10}$-SUMO2
U2OS His$_{10}$-ubiquitin
Thymidine 5.5h release
Thymidine 9.0h release
Thymidine 12h release
Thymidine14h release
Thymidine 16h release
Thymidine 20h release
Nocodazole 0.0h release
Nocodazole 0.5h release
Nocodazole 2.0h release
Nocodazole 4.0h release
Nocodazole 8.0h release
P3
P4
P5
Count
mCherry-A
(x 1,000)
